# Supplementary material for: Design and Synthesis of Novel Amino and Acetamidoaurones with Antimicrobial Activities
Source: Antibiotics (Basel). 2024 Mar 26;13(4):300. doi: 10.3390/antibiotics13040300 (PMC11047580; doi:10.3390/antibiotics13040300)
Supplement: Supplementary file 1 [file antibiotics-13-00300-s001.zip › antibiotics-2908642-supplementary.pdf]

Design and Synthesis of novel amino and acetamidoaurones with antimicrobial activities.

Attilio Di Maio [1,#], Hamza Olleik [2,#], Elise Courvoisier-Dezord [2], Sophie Guillier [3], Fabienne Ripoll [3], Romain Haudecoeur [4], Jean-Michel Bolla [3], Magali Casanova [5], Jean-François Cavalier [5], Stéphane Canaan [5], Valérie Pique [1], Yolande Charmasson [2], Elias Baydoun [6], Akram Hijazi [7], Josette Perrier [2], Marc Maresca [2,*], Maxime Robin [1,*]

NMR Spectra of compounds 4-34

N-[(2*Z*)-2-(2-methoxybenzylidene)-3-oxo-2,3-dihydro-1-benzofuran-5-yl]acetamide

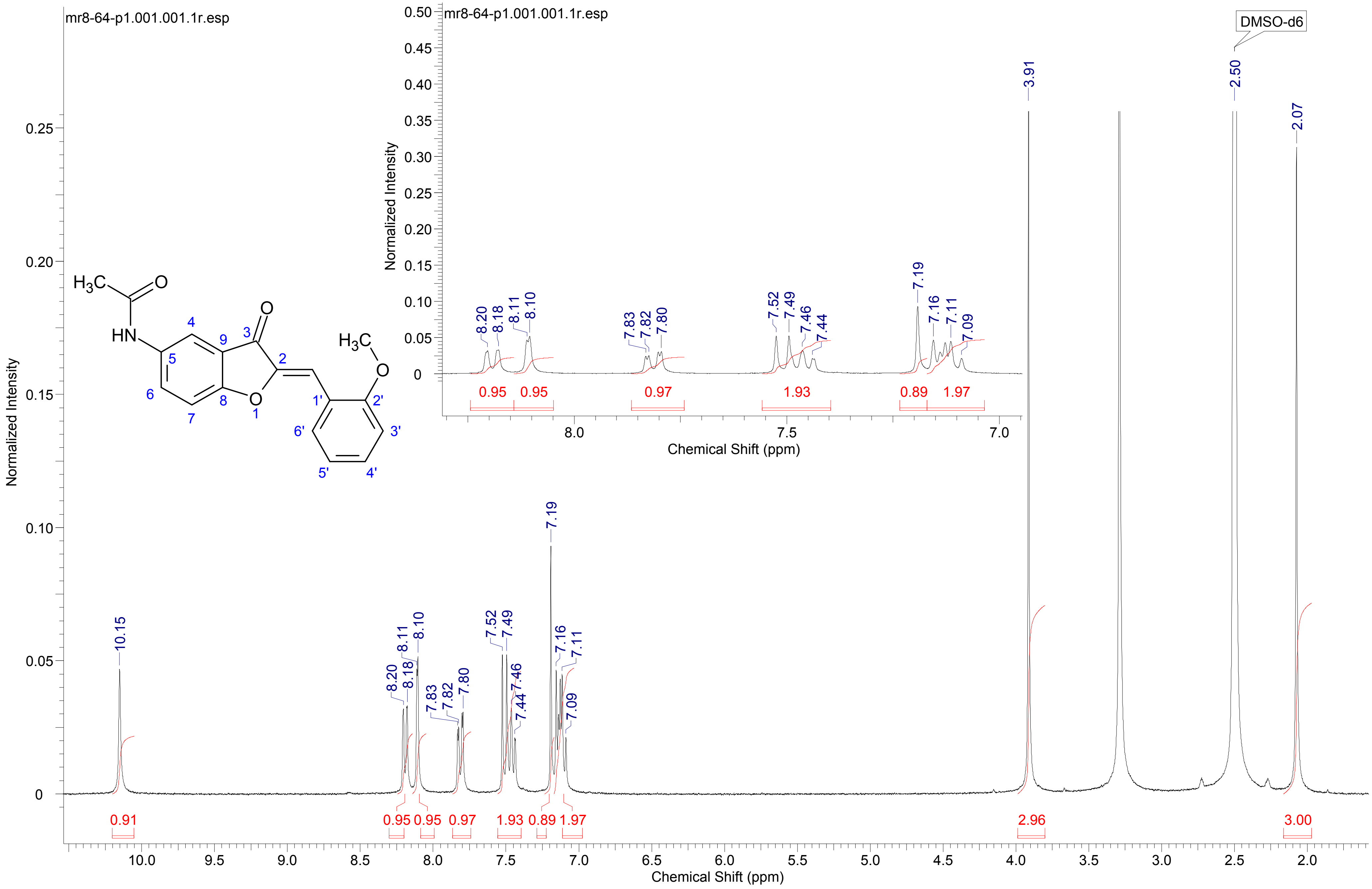

C:\Users\max\Documents\RMN\MR8-2017\mr8-64-p1\mr8-64-p1.001.001.1r.esp

MR983.001.001.1r.esp

183.53	C-4
168.41	CO
161.21	C-8
158.32	C-2'
146.75	C-2
135.53	C-5
131.99	C-4'
131.11	C-6'
128.71	C-6
120.89	C-1'
120.68	C-5'
120.08	C-9
113.31	C-3'
113.15	C-7
111.57	C-2
105.47	C-Au
55.84	OCH3
23.83	CH3

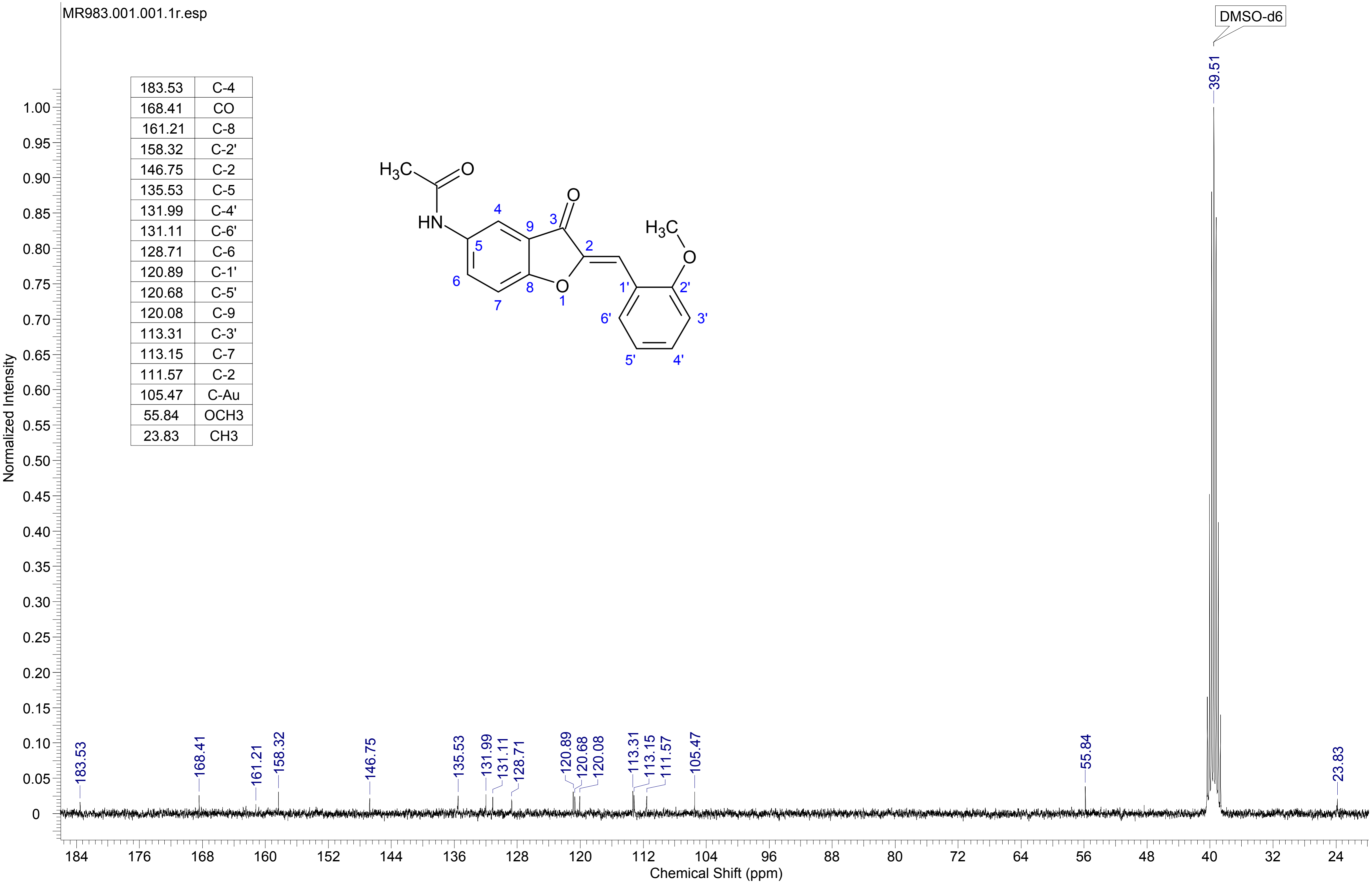

C:\Users\max\Documents\RMN\MR8-2017\MR983\MR983.001.001.1r.esp

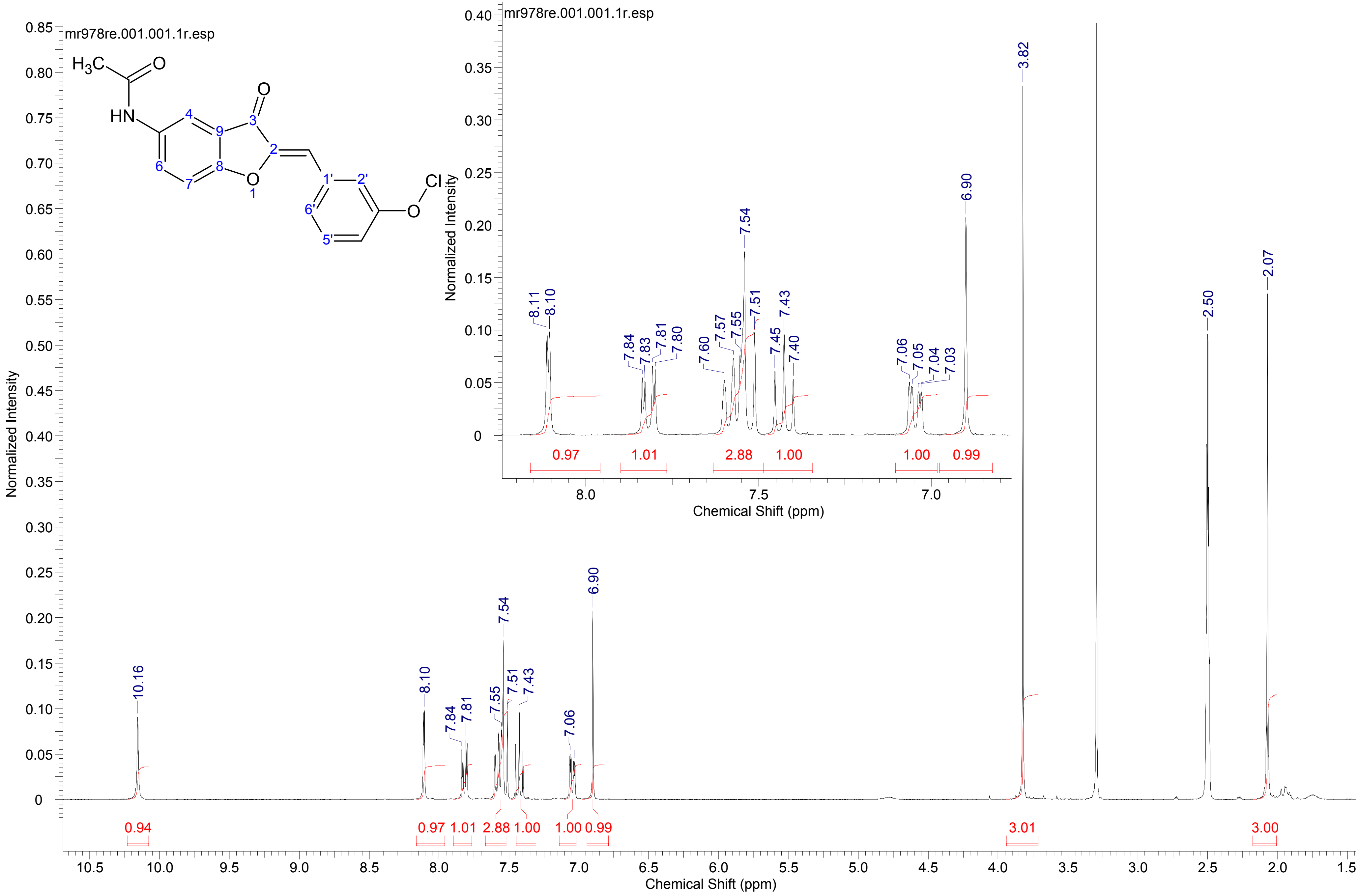

C:\Documents and Settings\RMN\2018\mr978re\mr978re.001.001.1r.esp

MR978REC.001.001.1r.esp

183.68	C-3
168.42	CO
161.33	C-8
159.42	C-3'
146.88	C-2
135.58	C-5
133.08	C-6'
130	C-2'
128.82	C-6
123.74	C-1'
120.59	C-9
116.57	C-6'
115.76	C-4'
113.35	C-7
113.15	C-4
112.07	C-Au
55.15	OCH3
23.83	CH3

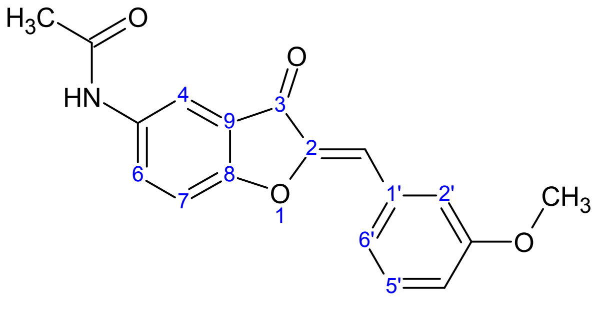

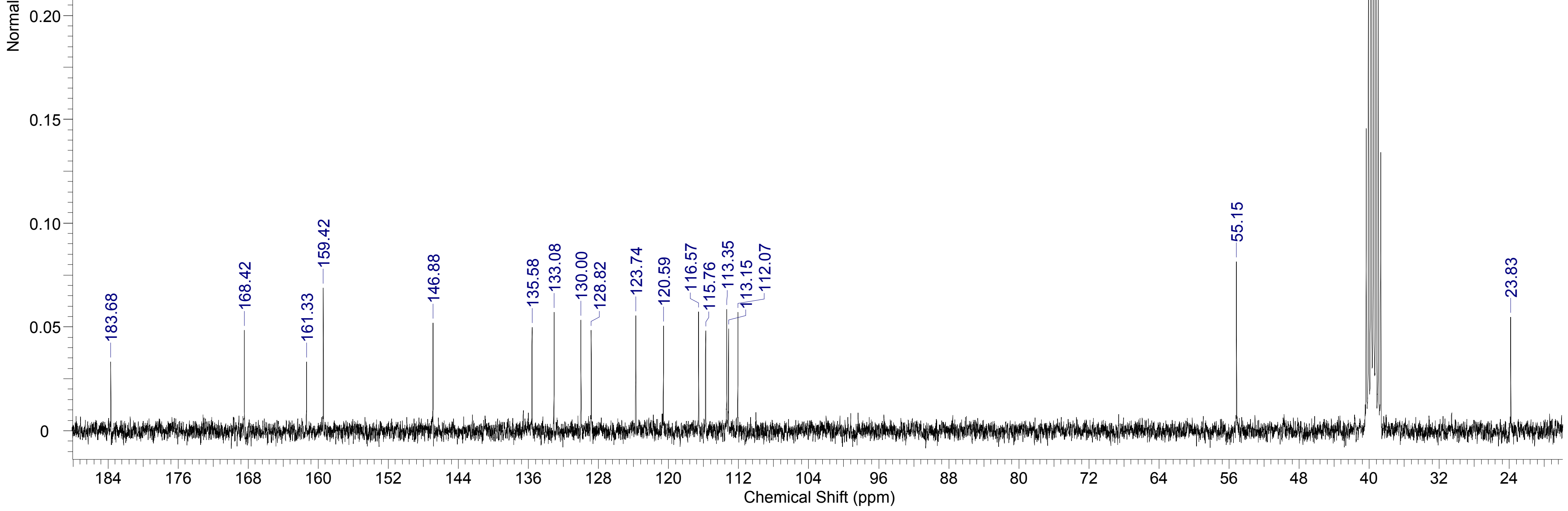

C:\Documents and Settings\RMN\2018\MR978REC\MR978REC.001.001.1r.esp

N-[(2*Z*)-2-(4-methoxybenzylidene)-3-oxo-2,3-dihydro-1-benzofuran-5-yl]acetamide

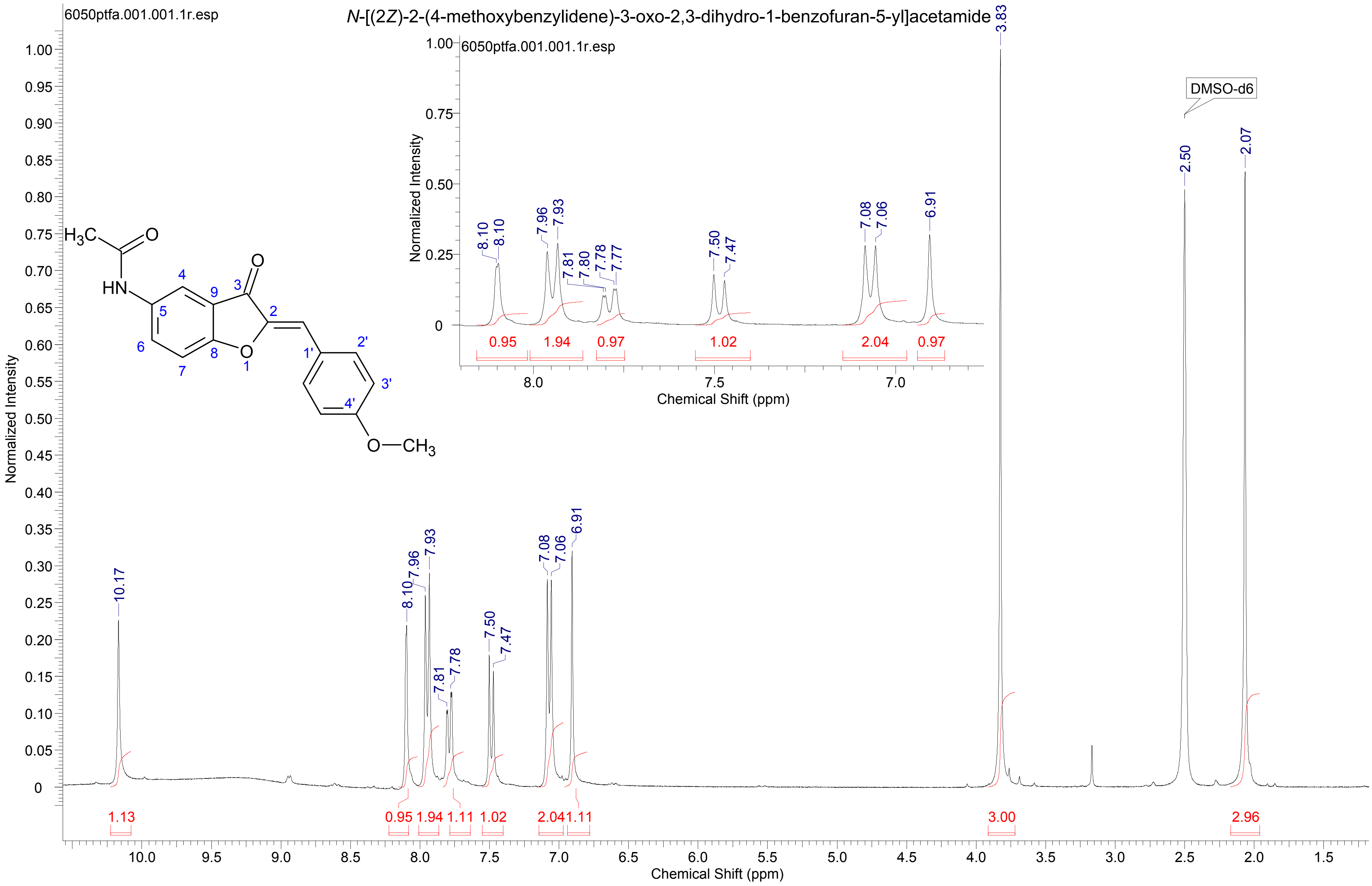

C:\Users\max\Documents\RMN\MR6\6050ptfa\6050ptfa.001.001.1r.esp

MR761C.001.001.1r.esp

N-[(2*Z*)-2-(4-methoxybenzylidene)-3-oxo-2,3-dihydro-1-benzofuran-5-yl]acetamide

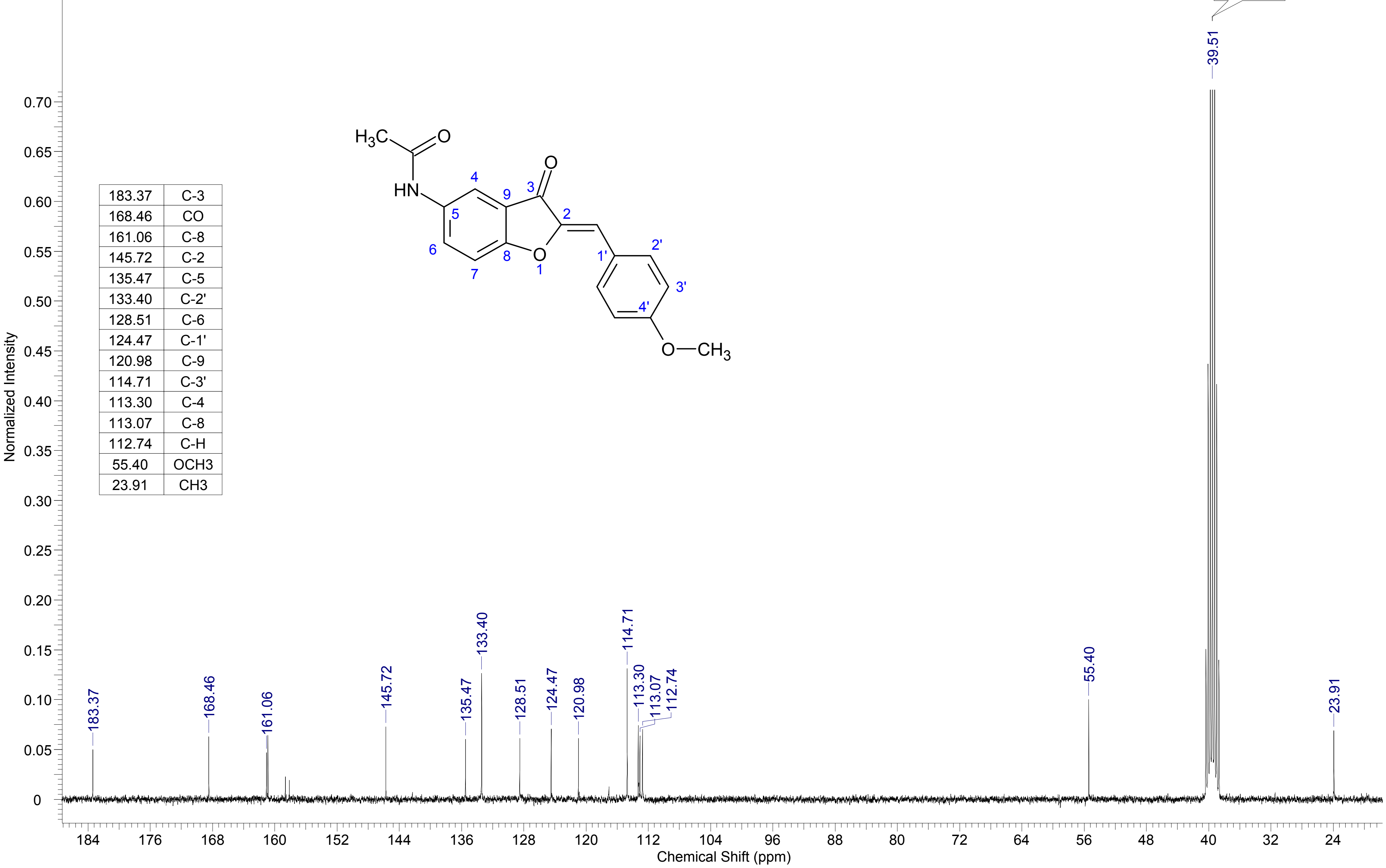

183.37	C-3
168.46	CO
161.06	C-8
145.72	C-2
135.47	C-5
133.40	C-2'
128.51	C-6
124.47	C-1'
120.98	C-9
114.71	C-3'
113.30	C-4
113.07	C-8
112.74	C-H
55.40	OCH3
23.91	CH3

C:\Users\max\Desktop\RMNredac\RMNfile\MR761C\MR761C.001.001.1r.esp

(2Z)-5-amino-2-(2-methoxybenzylidene)-1-benzofuran-3(2H)-one

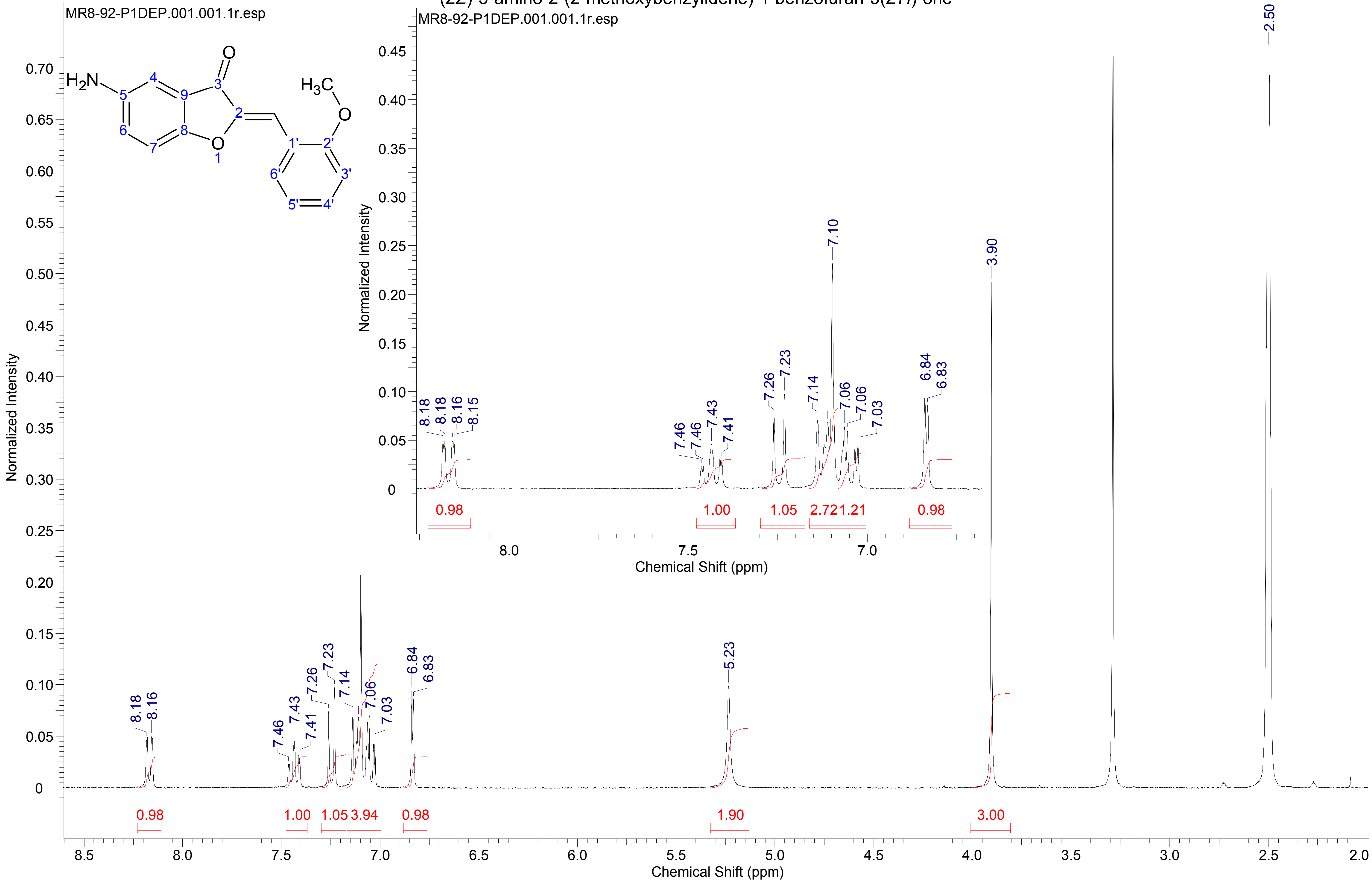

C:\Documents and Settings\RMN\2018\MR8-92-P1DEP\MR8-92-P1DEP.001.001.1r.esp

MR1012.001.001.1r.esp

184.09	C-3
158.13	C-2'
158	C-8
147.05	C-2
145.56	C-5
131.55	C-6'
130.98	C-4'
124.55	C-6
121.01	C-9
120.84	C-5'
120.41	C-1'
113.14	C-7
111.47	C-Au
105.44	C-3'
104.18	C-4
55.79	OCH3

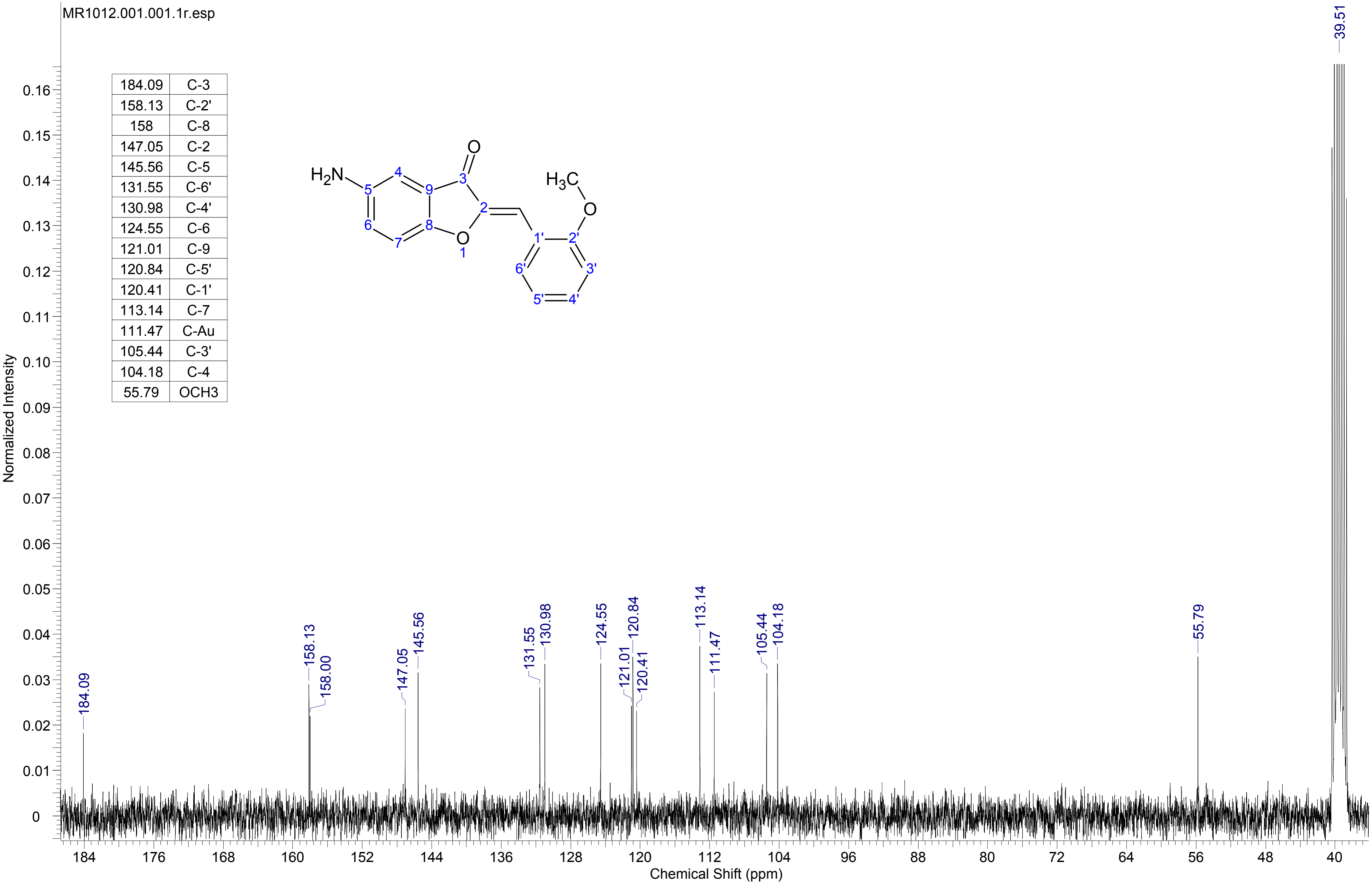

C:\Documents and Settings\RMN\2018\MR1012\MR1012.001.001.1r.esp

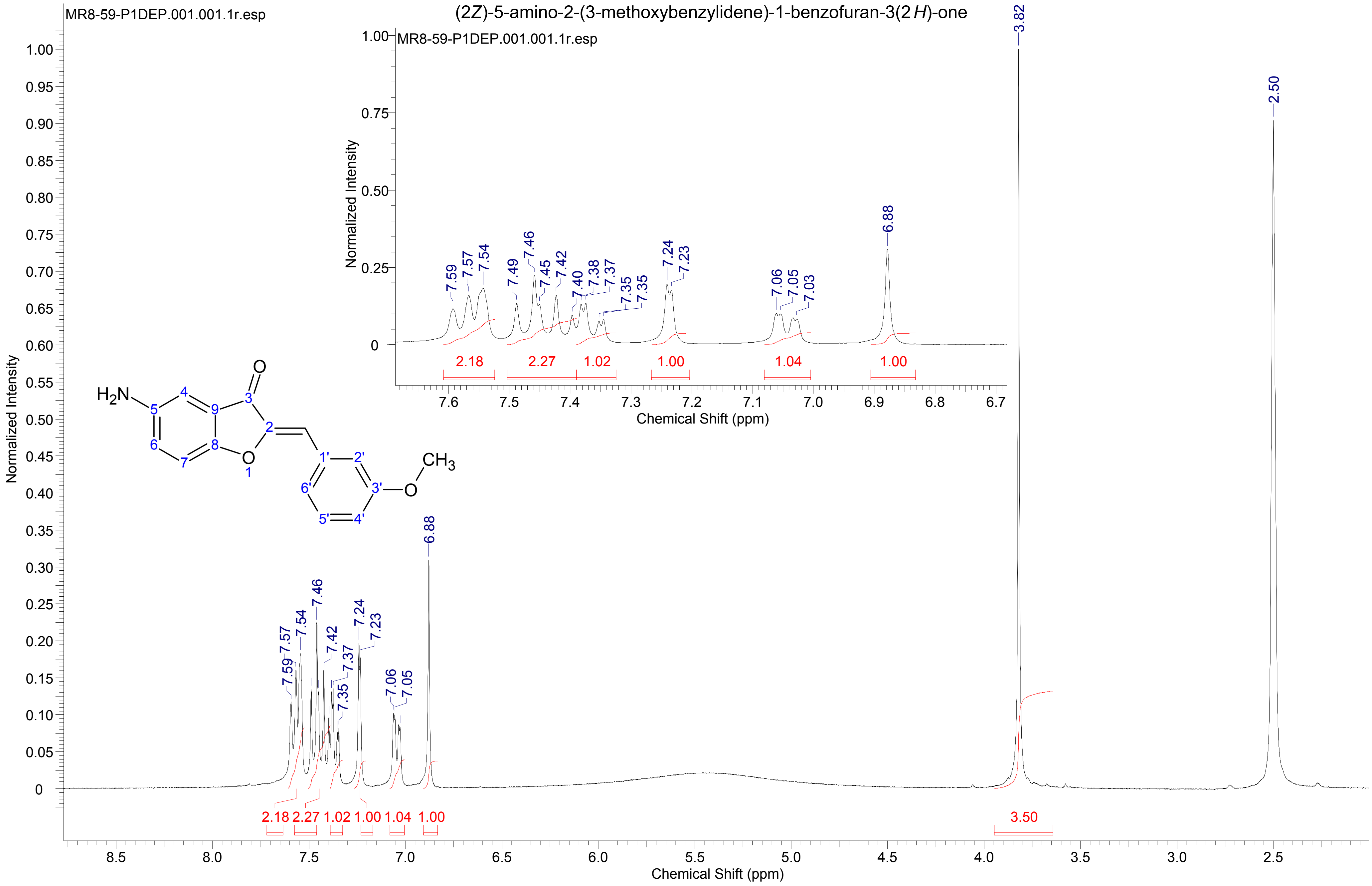

C:\Documents and Settings\RMN\2017\MR8-59-P1DEP\MR8-59-P1DEP.001.001.1r.esp

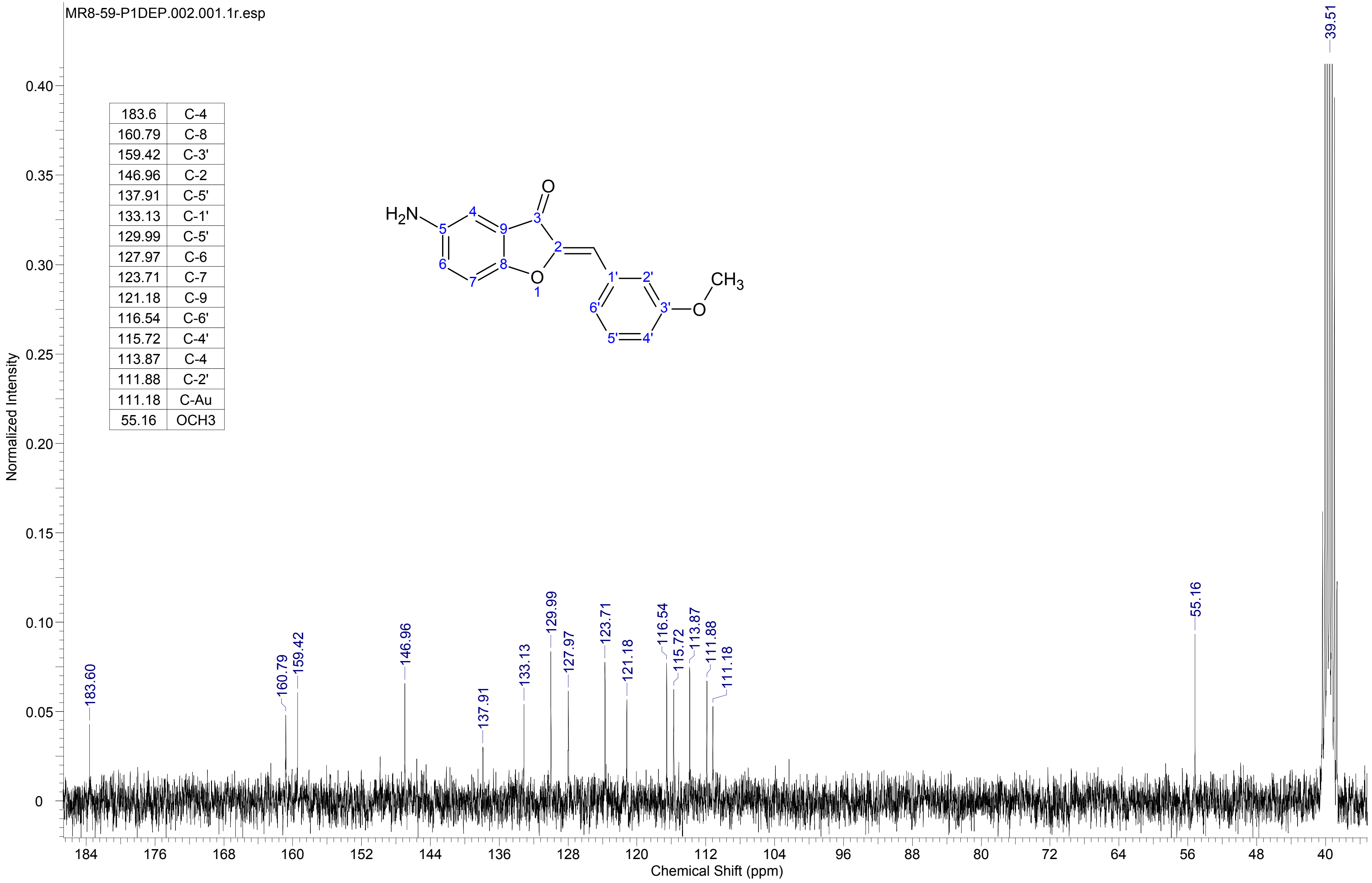

183.6	C-4
160.79	C-8
159.42	C-3'
146.96	C-2
137.91	C-5'
133.13	C-1'
129.99	C-5'
127.97	C-6
123.71	C-7
121.18	C-9
116.54	C-6'
115.72	C-4'
113.87	C-4
111.88	C-2'
111.18	C-Au
55.16	OCH3

C:\Documents and Settings\RMN\2017\MR8-59-P1DEP\MR8-59-P1DEP.002.001.1r.esp

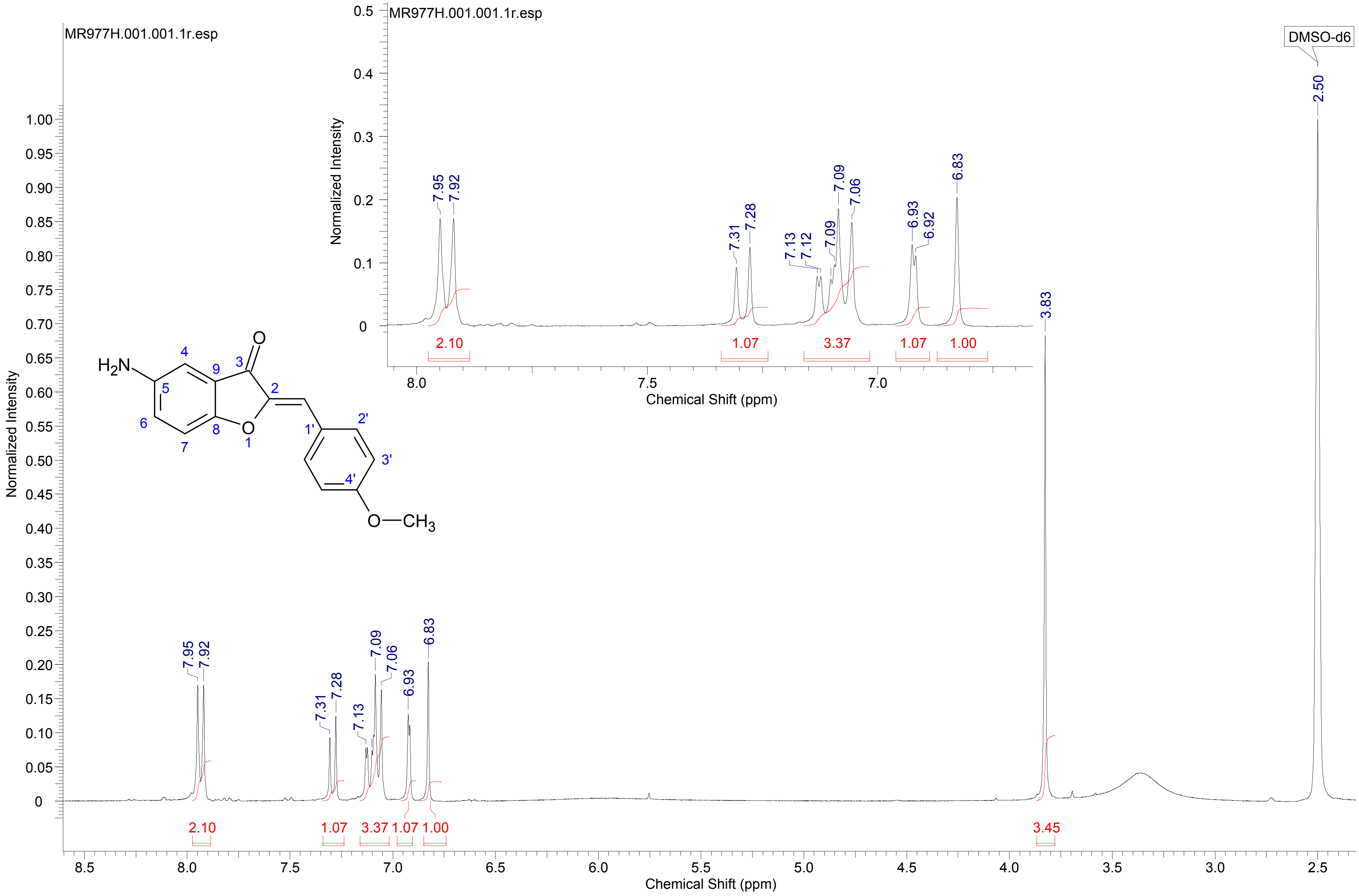

C:\Users\max\Documents\RMN\MR8-2017\MR977H\MR977H.001.001.1r.esp

MR977H.002.001.1r.esp

183.77	C-4
160.61	C-4'
158.51	C-8
145.94	C-5
143.60	C-2
133.16	C-2'
125.22	C-1'
124.69	C-6
121.35	C-9
114.65	C-3'
113.30	C-4
111.68	C-au
106.77	C-7
55.37	OCH3

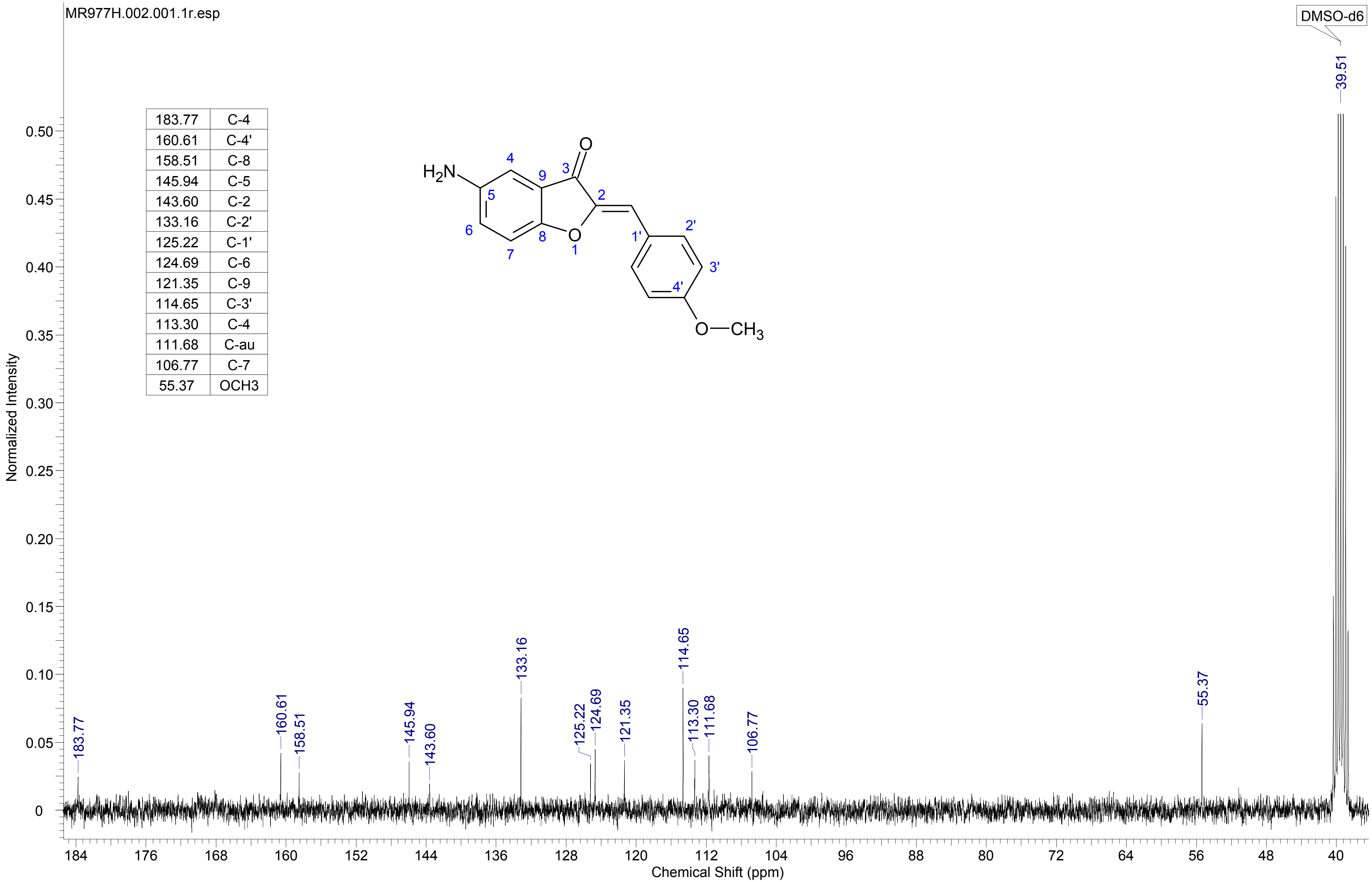

C:\Users\max\Documents\RMN\MR8-2017\MR977H\MR977H.002.001.1r.esp

N-{(2*Z*)-2-[3-(benzyloxy)benzylidene]-3-oxo-2,3-dihydro-1-benzofuran-5-yl}acetamide

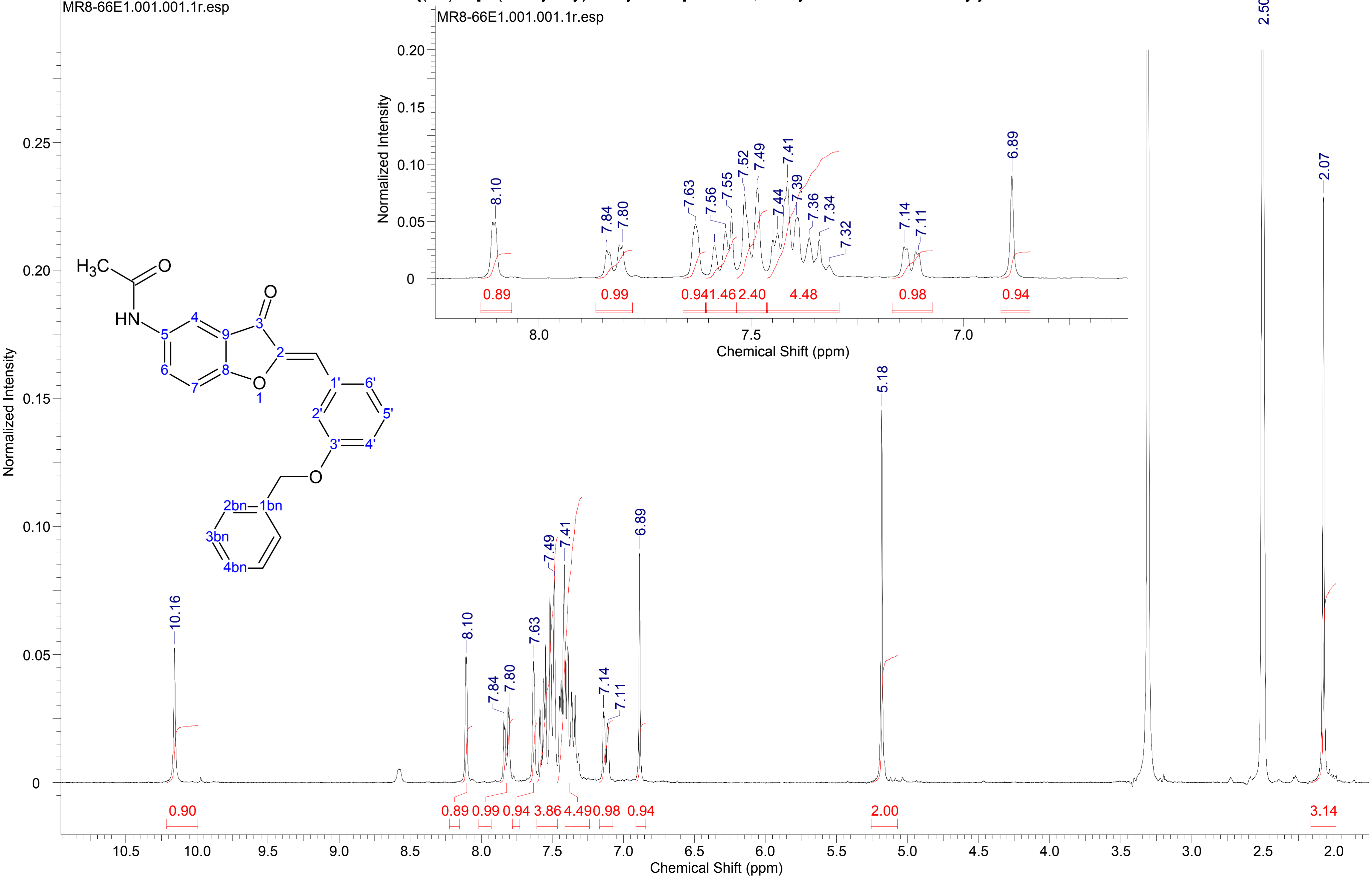

C:\Documents and Settings\RMN\2017\MR8-66E1\MR8-66E1.001.001.1r.esp

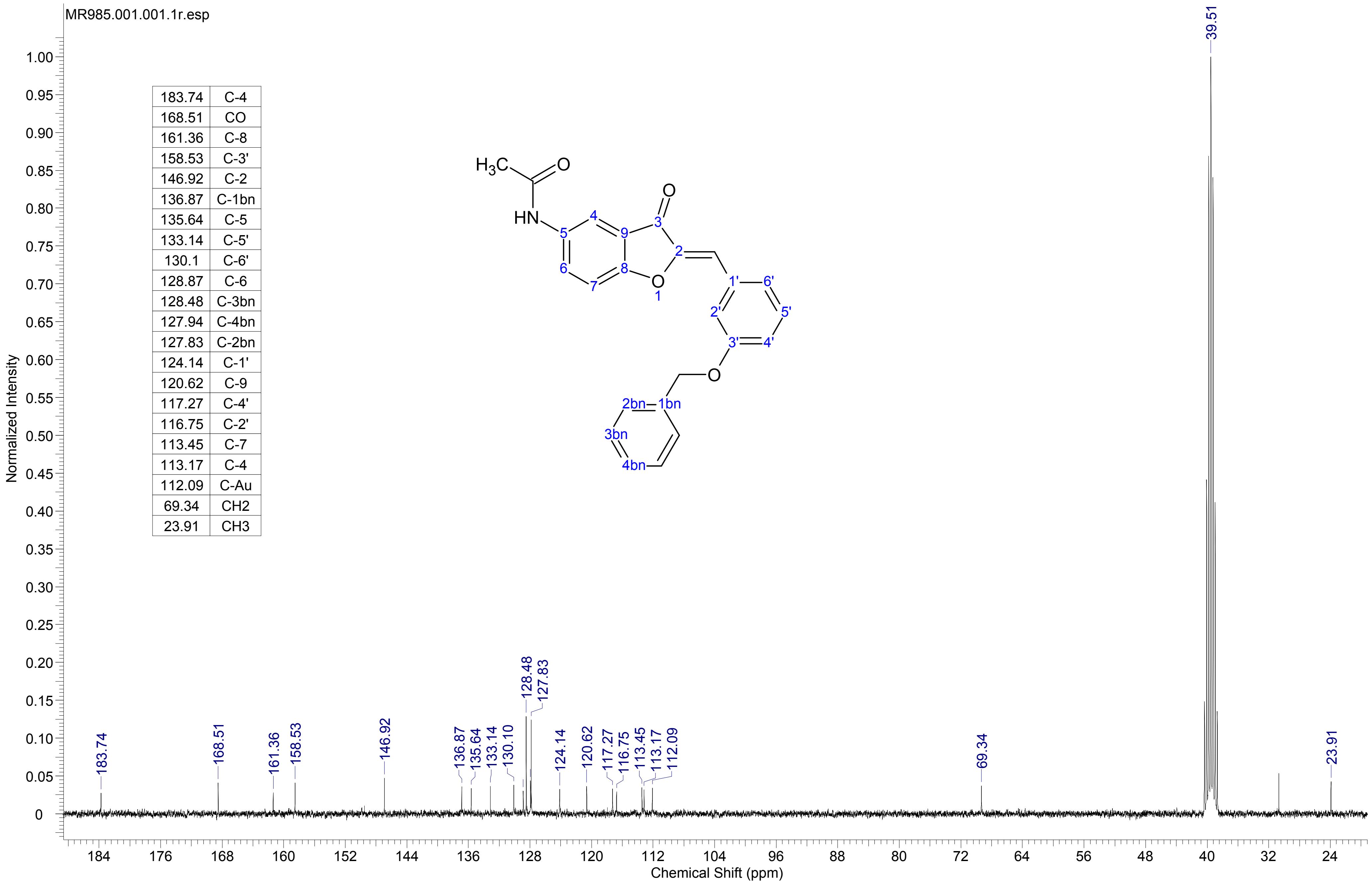

183.74	C-4
168.51	CO
161.36	C-8
158.53	C-3'
146.92	C-2
136.87	C-1bn
135.64	C-5
133.14	C-5'
130.1	C-6'
128.87	C-6
128.48	C-3bn
127.94	C-4bn
127.83	C-2bn
124.14	C-1'
120.62	C-9
117.27	C-4'
116.75	C-2'
113.45	C-7
113.17	C-4
112.09	C-Au
69.34	CH2
23.91	CH3

N-{(2Z)-2-[4-(benzyloxy)benzylidene]-3-oxo-2,3-dihydro-1-benzofuran-5-yl}acetamide

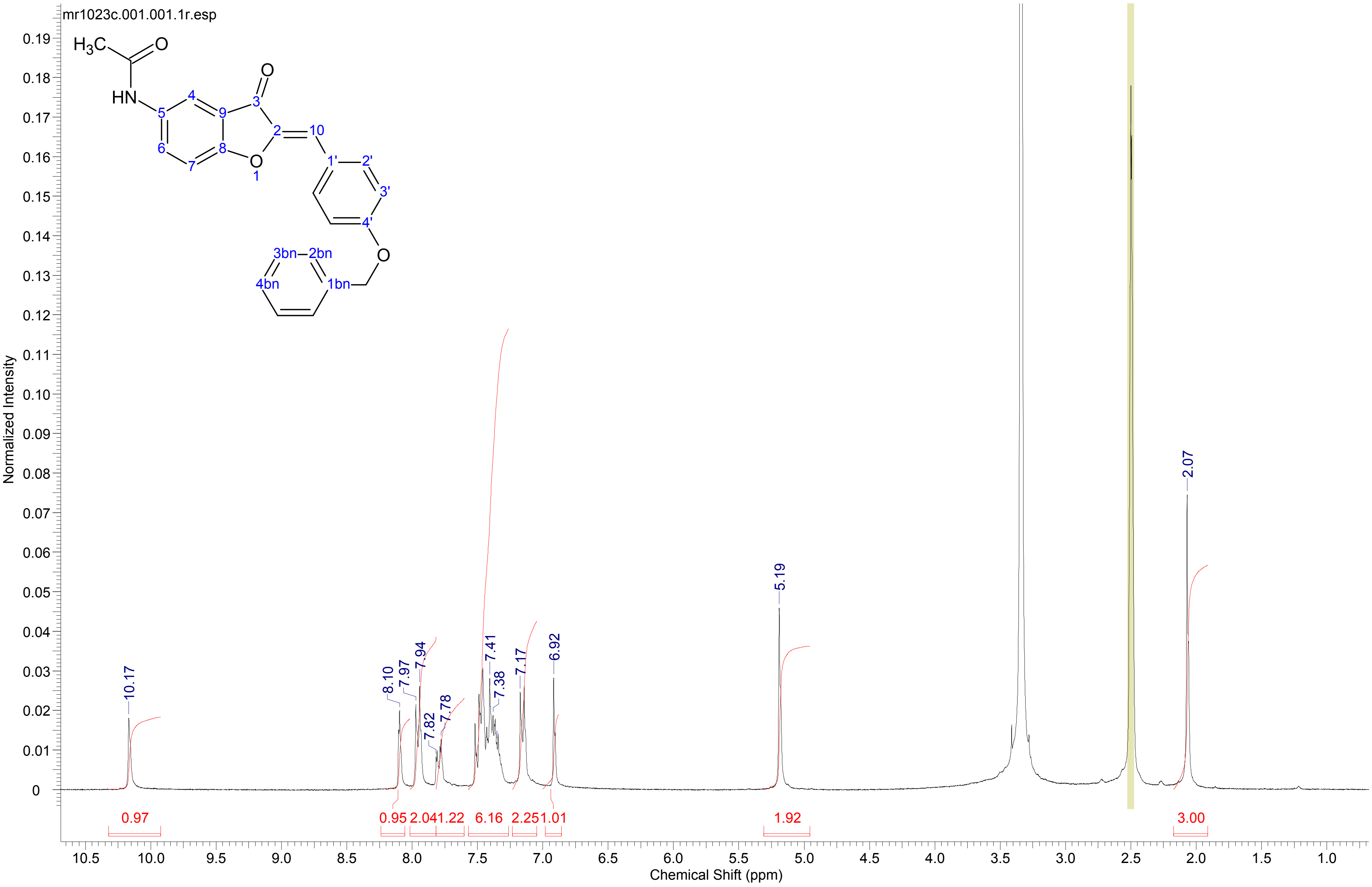

C:\Documents and Settings\RMN\MR9\mr1023c\mr1023c.001.001.1r.esp

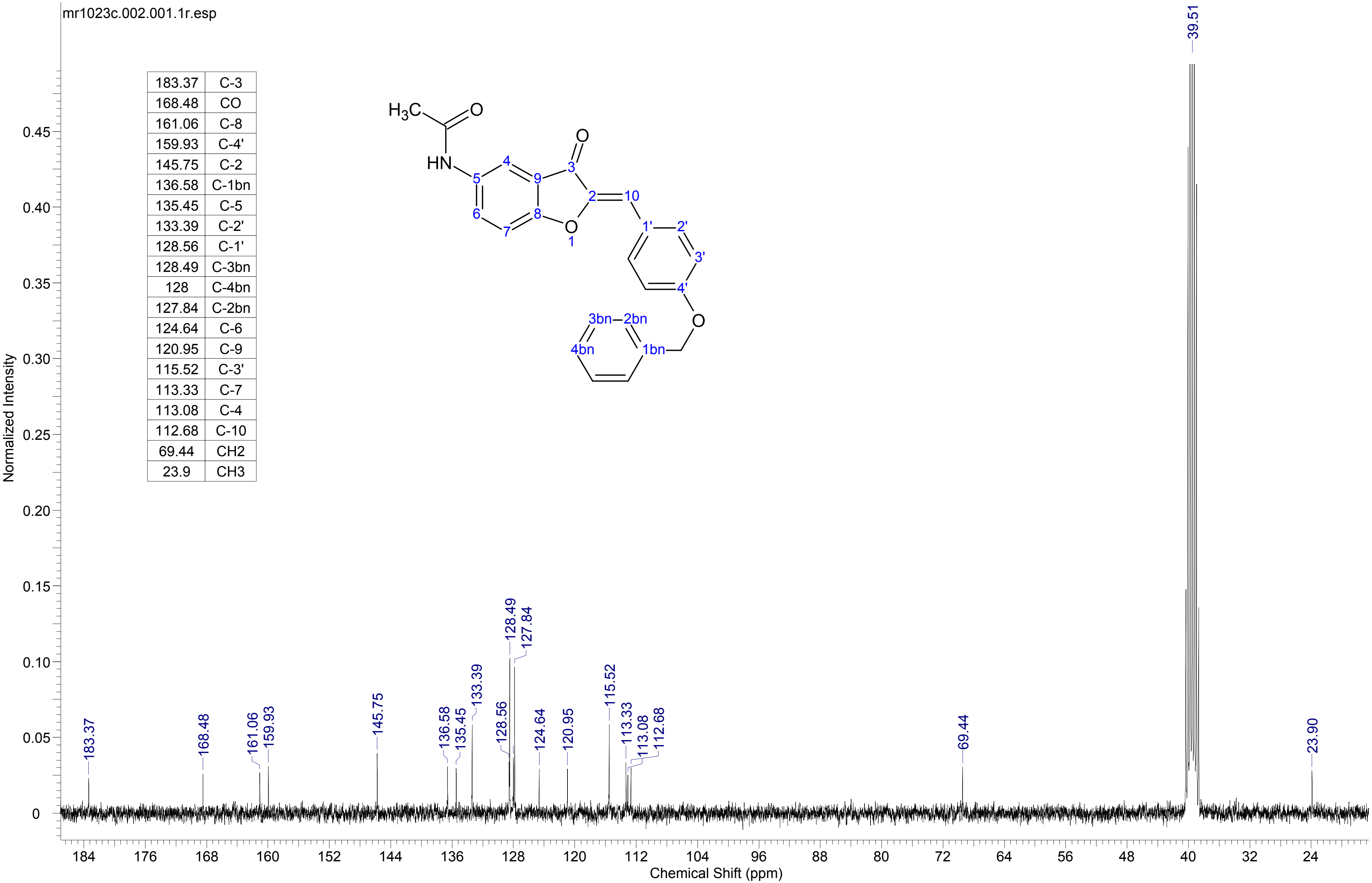

183.37	C-3
168.48	CO
161.06	C-8
159.93	C-4'
145.75	C-2
136.58	C-1bn
135.45	C-5
133.39	C-2'
128.56	C-1'
128.49	C-3bn
128	C-4bn
127.84	C-2bn
124.64	C-6
120.95	C-9
115.52	C-3'
113.33	C-7
113.08	C-4
112.68	C-10
69.44	CH2
23.9	CH3

C:\Documents and Settings\RMN\MR9\mr1023c\mr1023c.002.001.1r.esp

(2Z)-5-amino-2-[3-(benzyloxy)benzylidene]-1-benzofuran-3(2H)-one

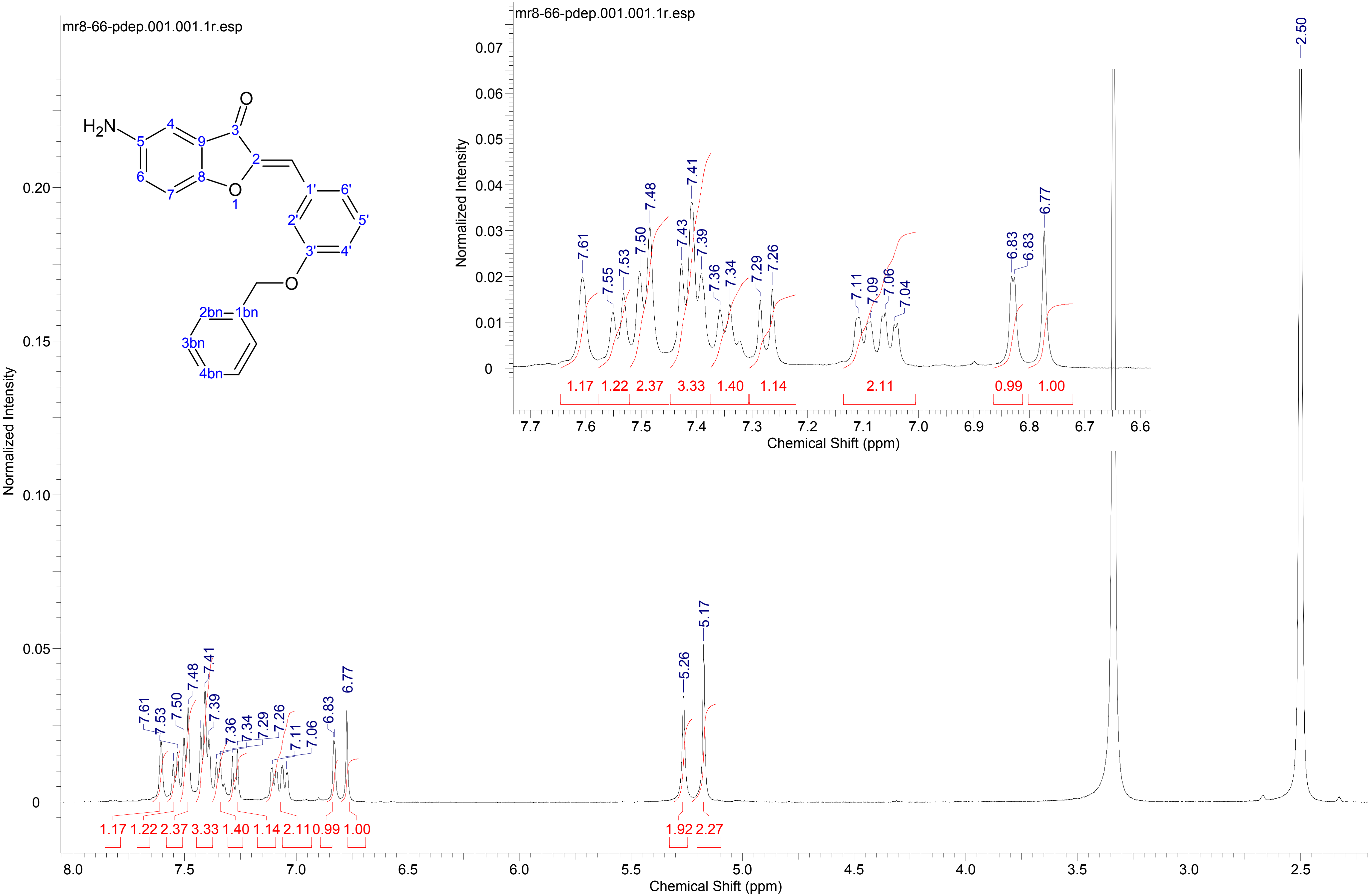

C:\Documents and Settings\RMN\2017\mr8-66-pdep\mr8-66-pdep.001.001.1r.esp

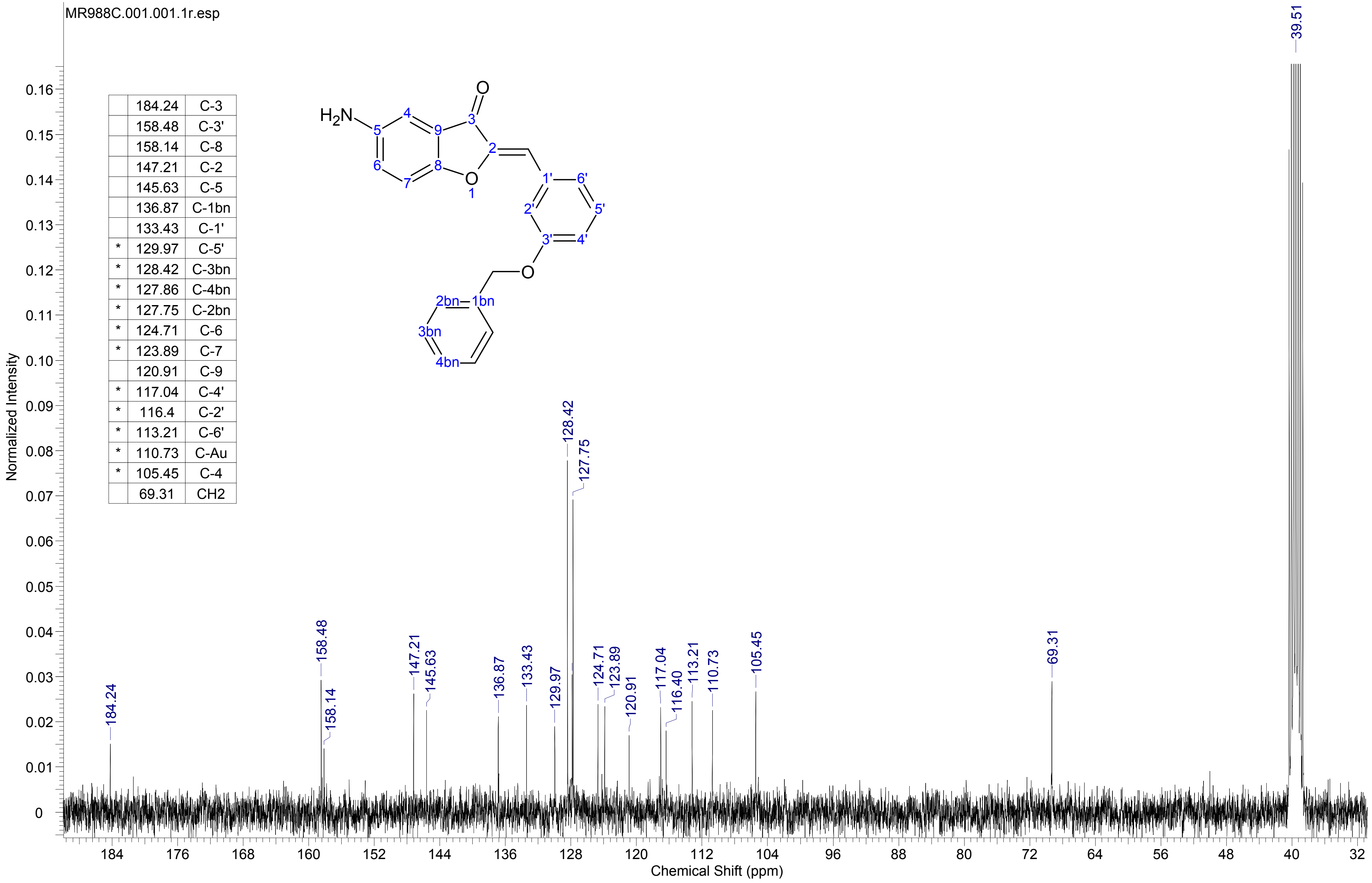

	184.24	C-3
	158.48	C-3'
	158.14	C-8
	147.21	C-2
	145.63	C-5
	136.87	C-1bn
	133.43	C-1'
*	129.97	C-5'
*	128.42	C-3bn
*	127.86	C-4bn
*	127.75	C-2bn
*	124.71	C-6
*	123.89	C-7
	120.91	C-9
*	117.04	C-4'
*	116.4	C-2'
*	113.21	C-6'
*	110.73	C-Au
*	105.45	C-4
	69.31	CH2

C:\Documents and Settings\RMN\2017\MR988C\MR988C.001.001.1r.esp

AD-4-112-2.001.001.1r.esp

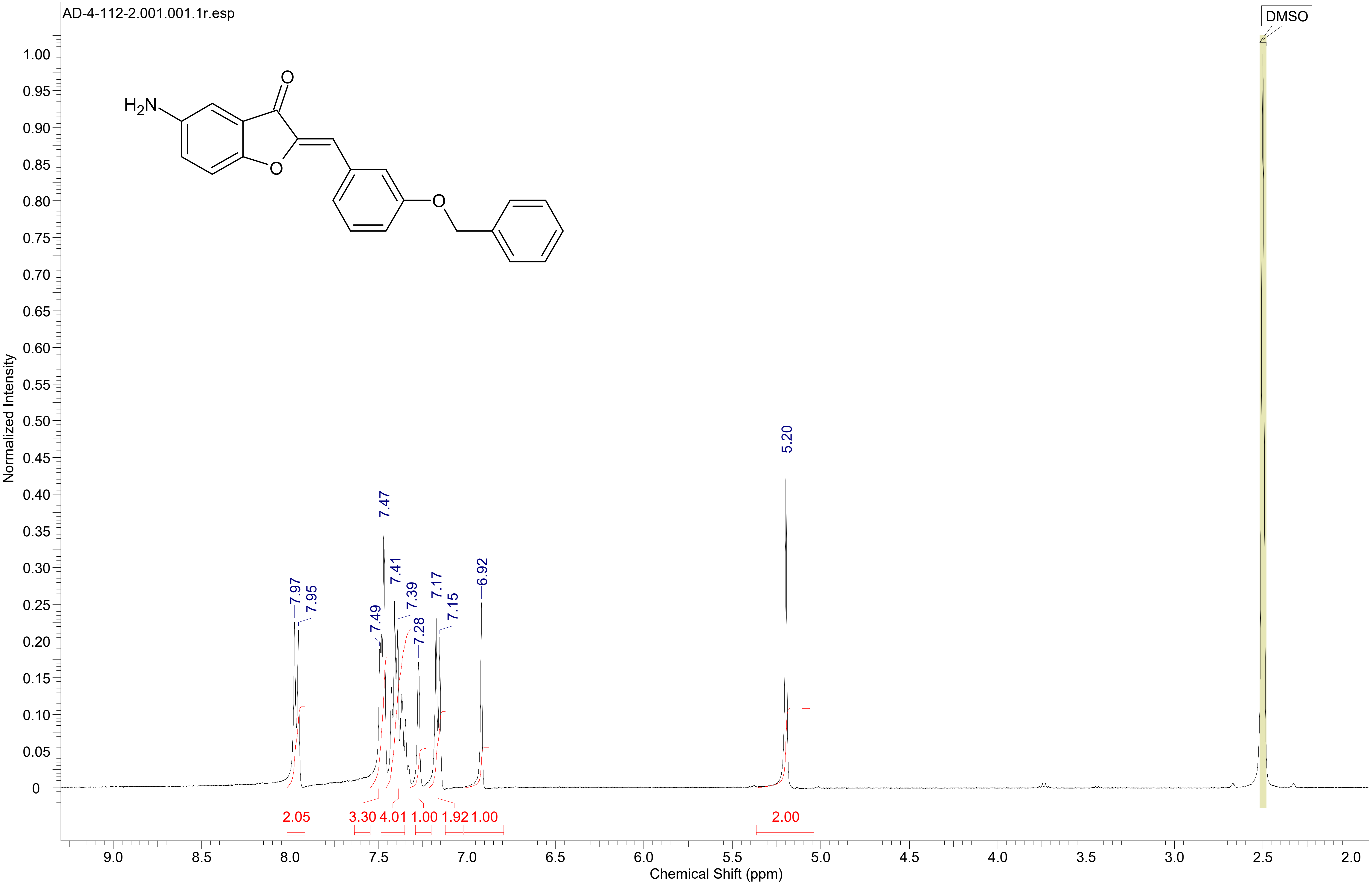

C:\Users\33674\Desktop\pubAuroneARediger\spectres\AD-4-112-2\AD-4-112-2.001.001.1r.esp

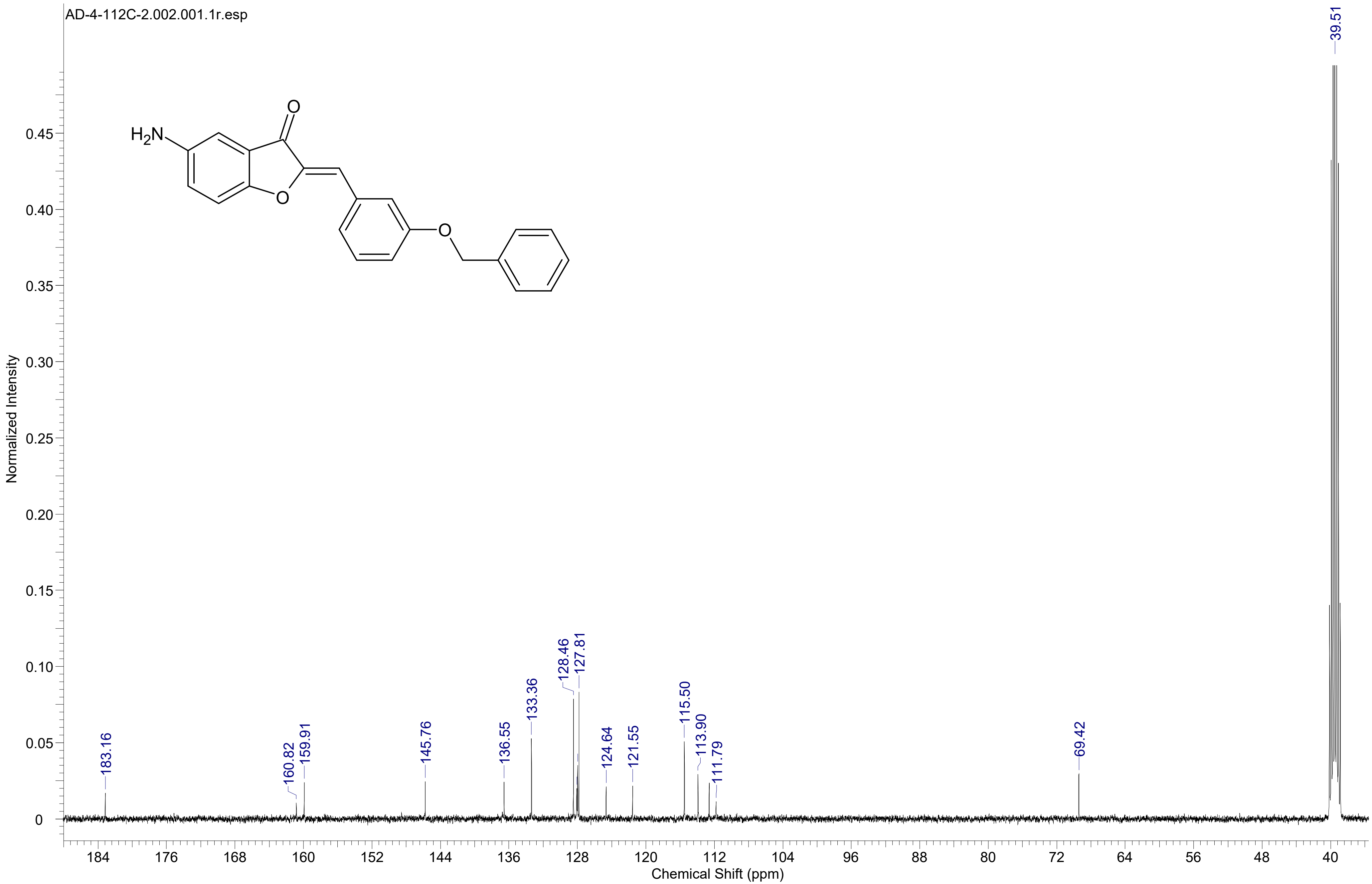

C:\Users\33674\Desktop\pubAuroneARediger\spectres\AD-4-112C-2\AD-4-112C-2.002.001.1r.esp

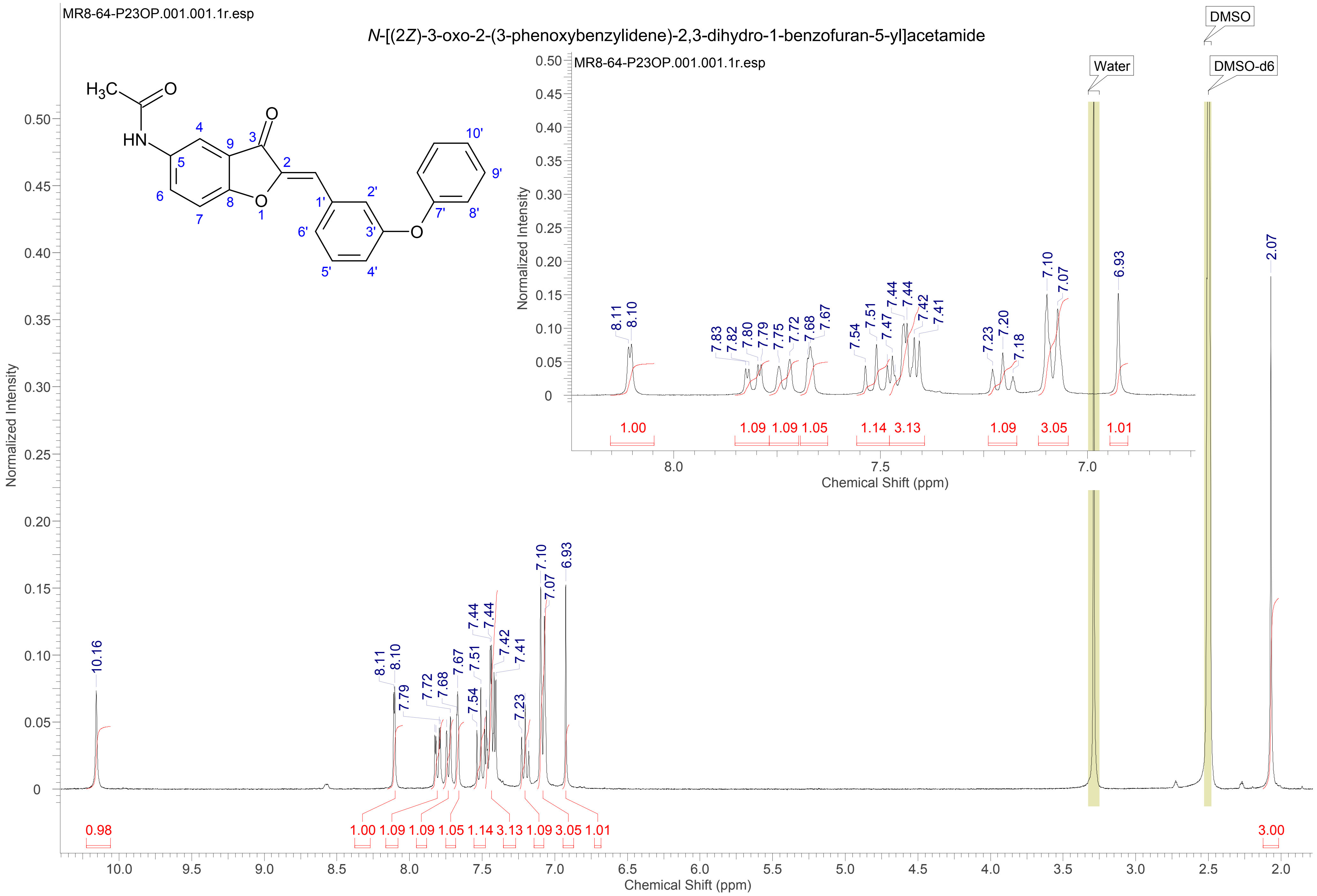

MR8-64-P23OP.001.001.1r.esp
N-[(2Z)-3-oxo-2-(3-phenoxybenzylidene)-2,3-dihydro-1-benzofuran-5-yl]acetamide
DMSO
Water
DMSO-d6
10.16
8.11
8.10
7.79
7.72
7.68
7.67
7.54
7.51
7.44
7.44
7.42
7.41
7.23
7.10
7.07
6.93
2.07
0.98
1.00
1.09
1.09
1.05
1.14
3.13
1.09
3.05
1.01
3.00
Normalized Intensity
Chemical Shift (ppm)

MR757C.001.001.1r.esp

183.72	C-3
168.52	CO
161.30	C-8
157.14	C-7'
156.19	C-3'
147.11	C-2
135.68	C-5
133.78	C-1'
130.60	C-6
130.17	C-9'
128.92	C-5'
126.54	C-9
123.88	C-4
120.62	C-6'
120.57	C-10'
119.97	C-4'
118.99	C-8'
113.30	C-2'
113.21	C-7
111.41	C-H
23.91	CH3

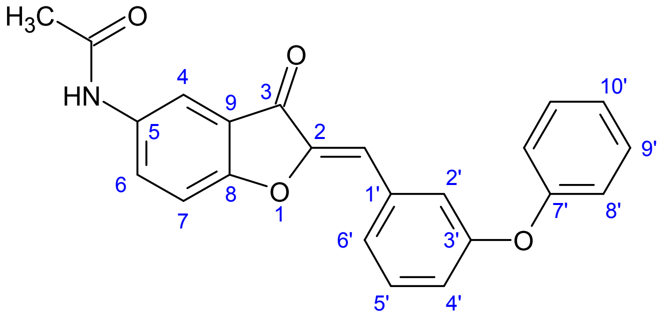

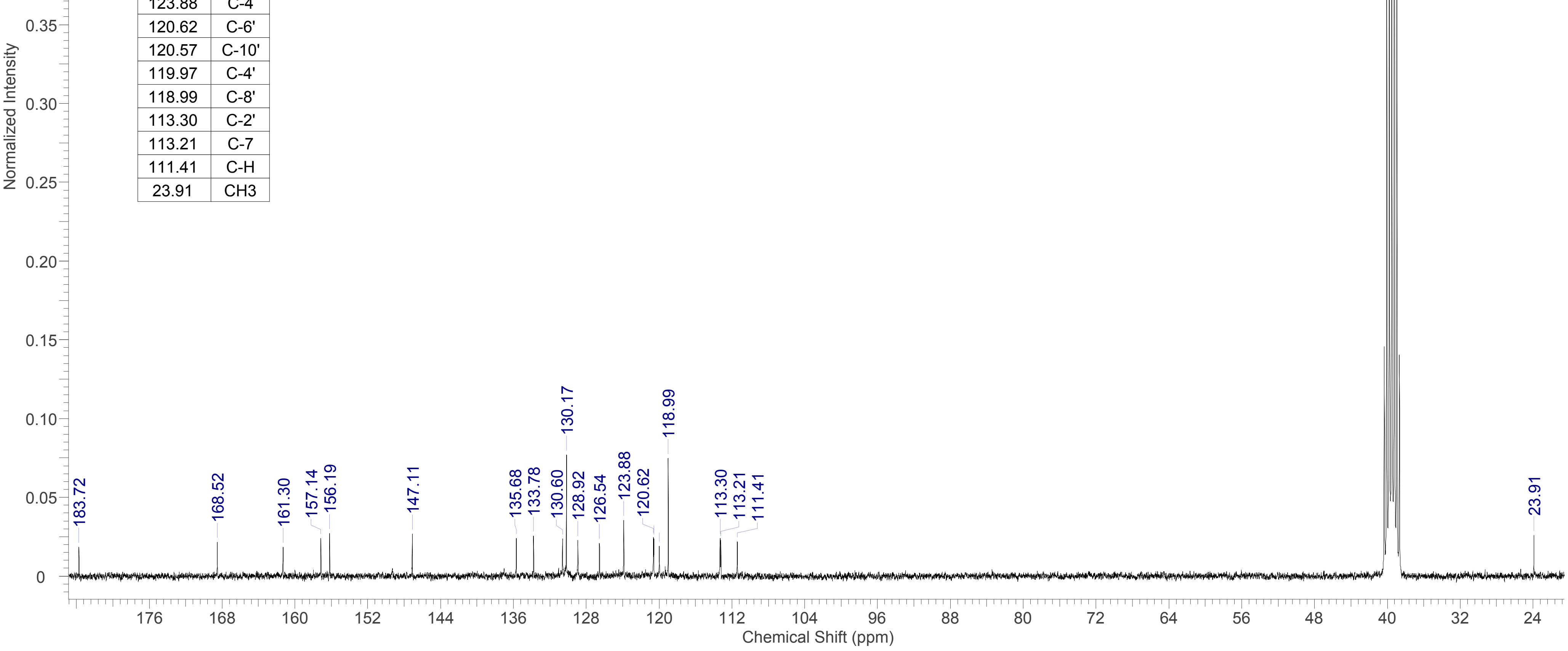

C:\Users\max\Desktop\RMNredac\RMNfile\MR757C\MR757C.001.001.1r.esp

N-[(2*Z*)-3-oxo-2-(4-phenoxybenzylidene)-2,3-dihydro-1-benzofuran-5-yl]acetamide

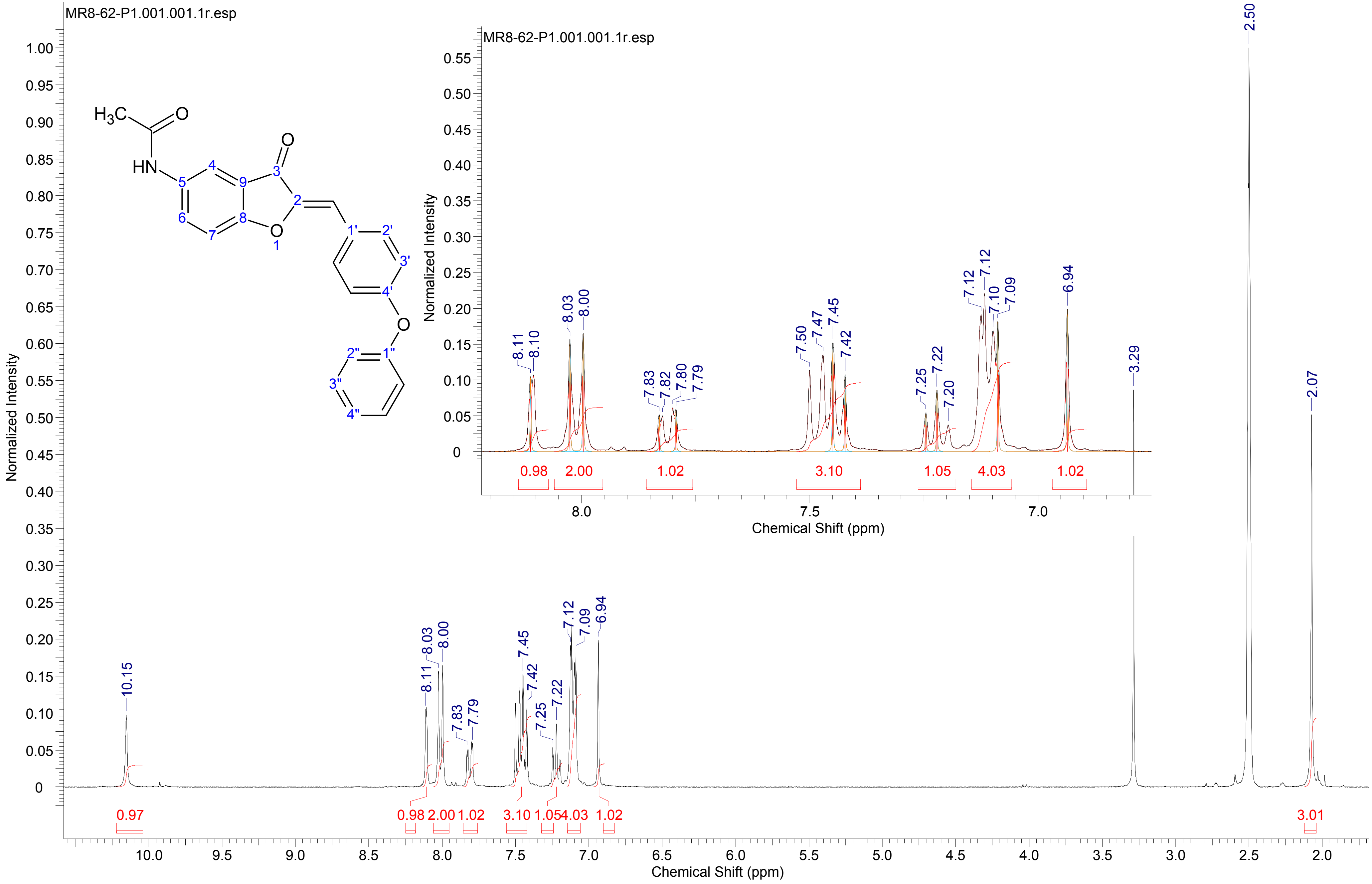

C:\Documents and Settings\RMN\2017\MR8-62-P1\MR8-62-P1.001.001.1r.esp

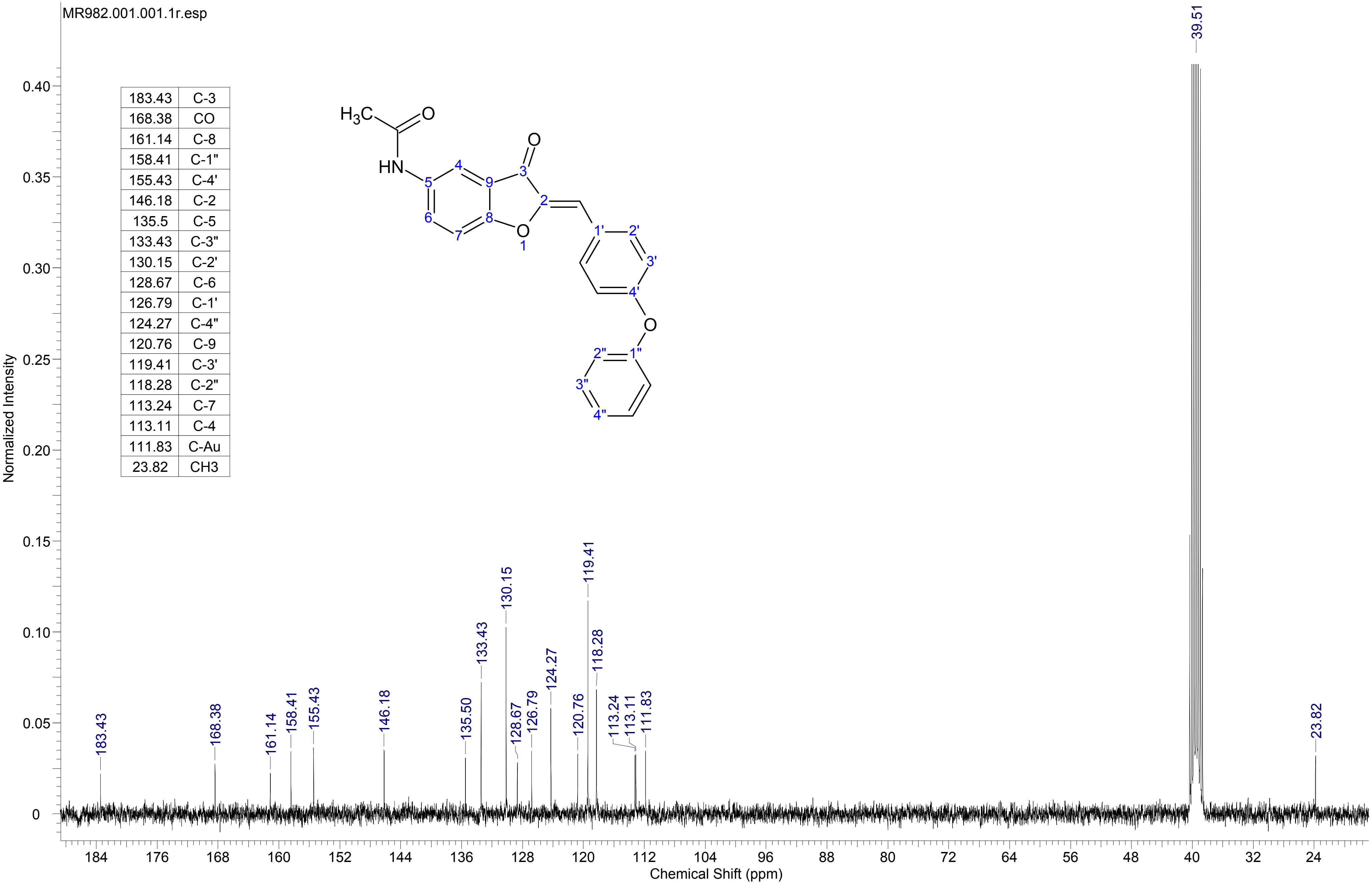

183.43	C-3
168.38	CO
161.14	C-8
158.41	C-1"
155.43	C-4'
146.18	C-2
135.5	C-5
133.43	C-3"
130.15	C-2'
128.67	C-6
126.79	C-1'
124.27	C-4"
120.76	C-9
119.41	C-3'
118.28	C-2"
113.24	C-7
113.11	C-4
111.83	C-Au
23.82	CH3

C:\Documents and Settings\RMN\2017\MR982\MR982.001.001.1r.esp

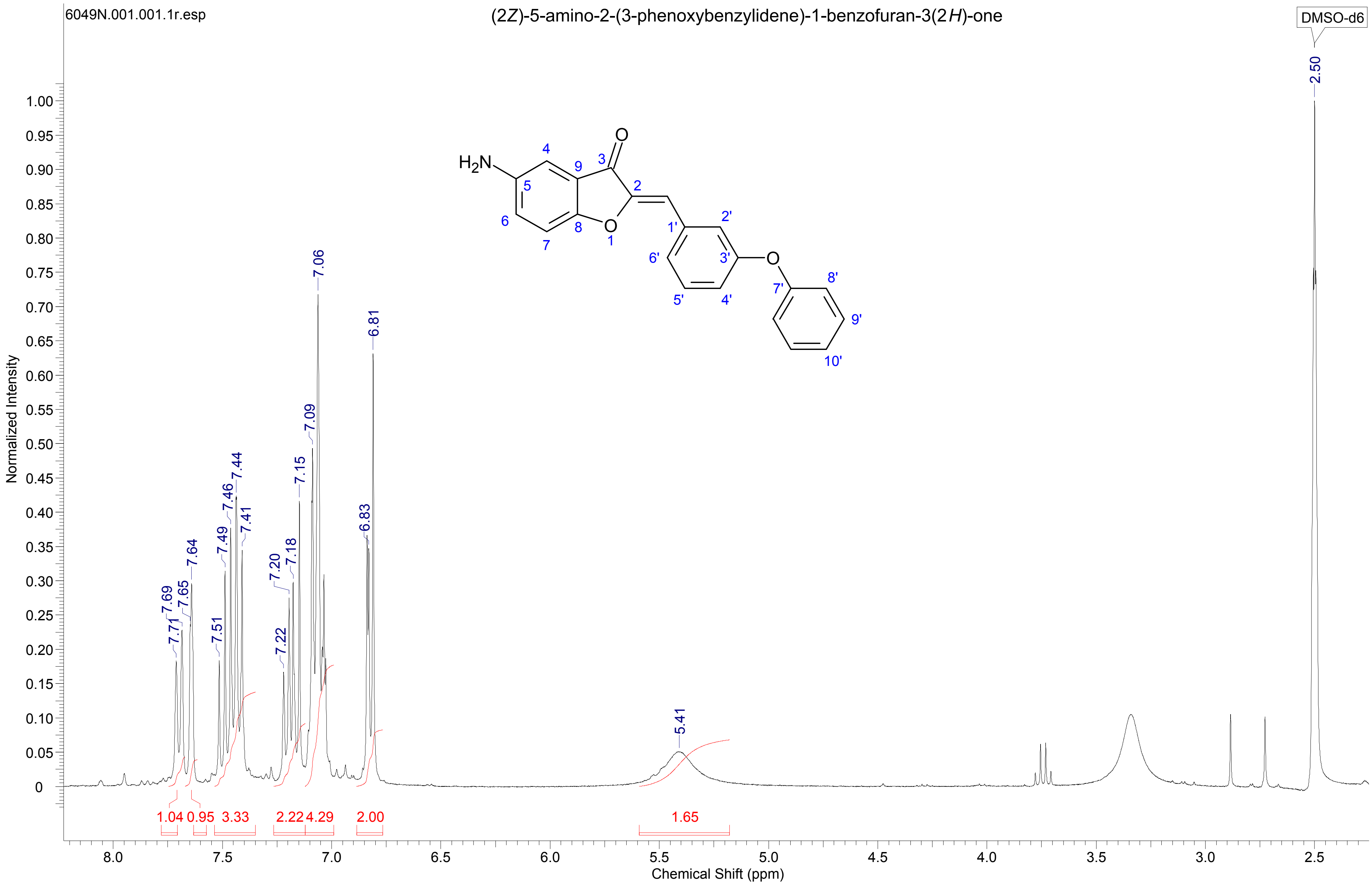

C:\Users\max\Documents\RMN\MR6\6049N\6049N.001.001.1r.esp

184.24	C-3
158.13	C-7'
157.10	C-3'
156.21	C-8
147.41	C-2
145.56	C-5
134.09	C-1'
130.52	C-5'
130.15	C-9'
126.34	C-6
124.82	C-9
123.81	C-6'
120.88	C-10'
120.43	C-4'
119.65	C-4
118.95	C-8'
113.13	C-8
110.13	C-7
105.61	C-H

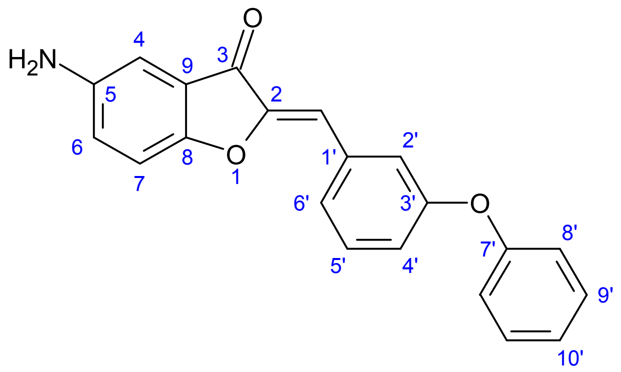

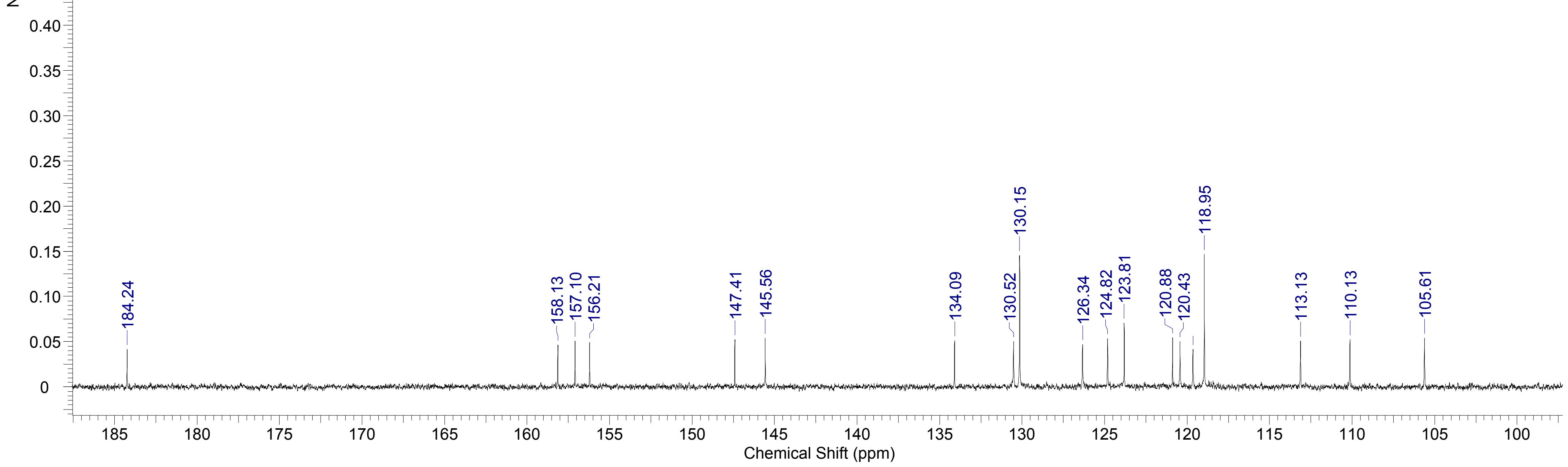

C:\Users\max\Desktop\RMNredac\RMNfile\mr759c\mr759c.001.001.1r.esp

N-[(2Z)-3-oxo-2-(4-phenoxybenzylidene)-2,3-dihydro-1-benzofuran-5-yl]acetamide

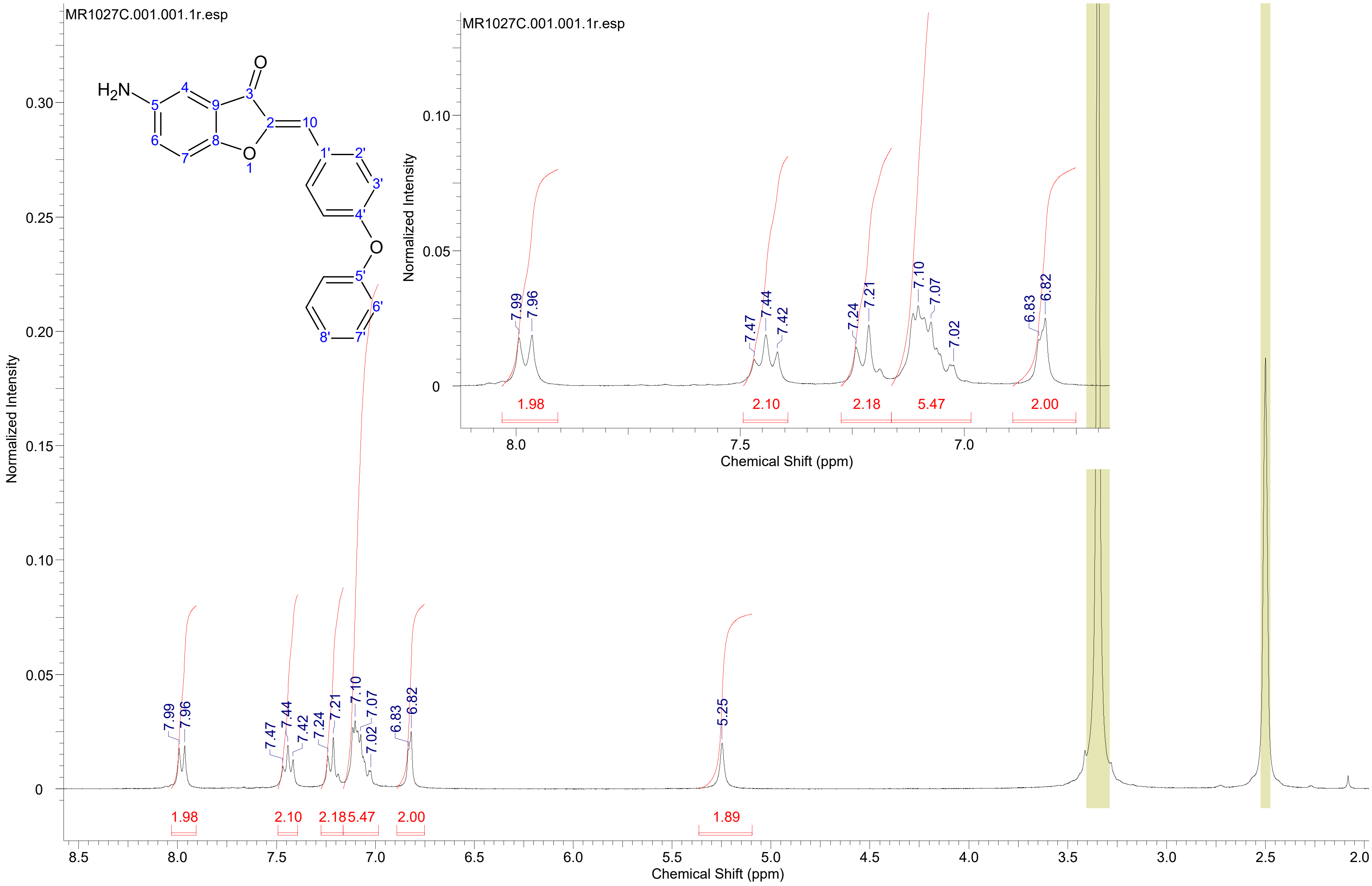

C:\Documents and Settings\RMN\MR9\MR1027C\MR1027C.001.001.1r.esp

MR1027C.002.001.1r.esp

184.11	C-3
158.16	C-5'
158.02	C-8
155.58	C-4'
146.56	C-2
145.6	C-5
133.27	C-7'
130.24	C-2'
127.21	C-1'
124.62	C-6
124.28	C-8'
121.15	C-9
119.41	C-3'
118.39	C-6'
113.17	C-4
110.62	C-10
105.45	C-7

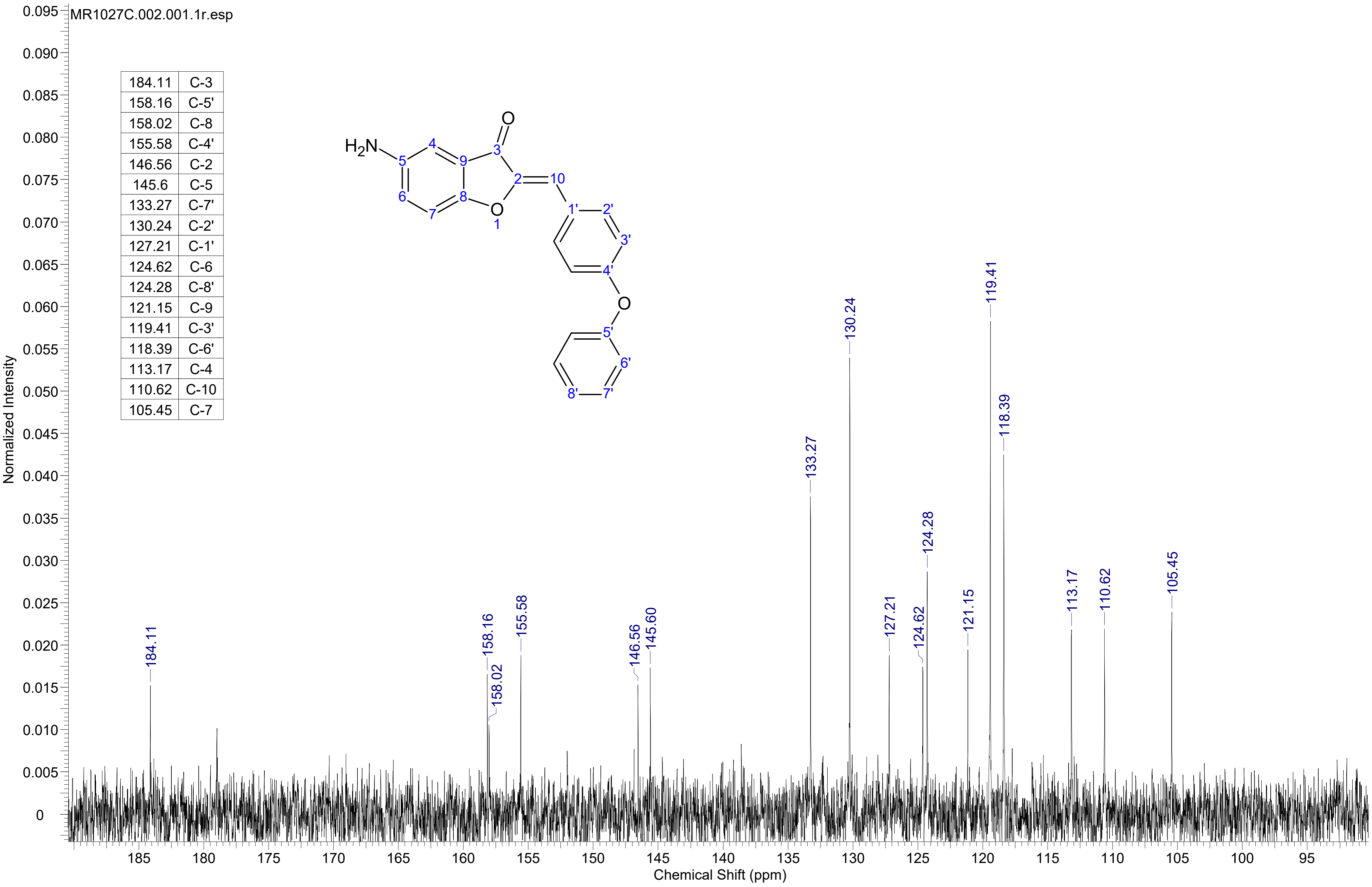

C:\Documents and Settings\RMN\MR9\MR1027C\MR1027C.002.001.1r.esp

N-{(2*Z*)-3-oxo-2-[2-(propan-2-yloxy)benzylidene]-2,3-dihydro-1-benzofuran-5-yl}acetamide

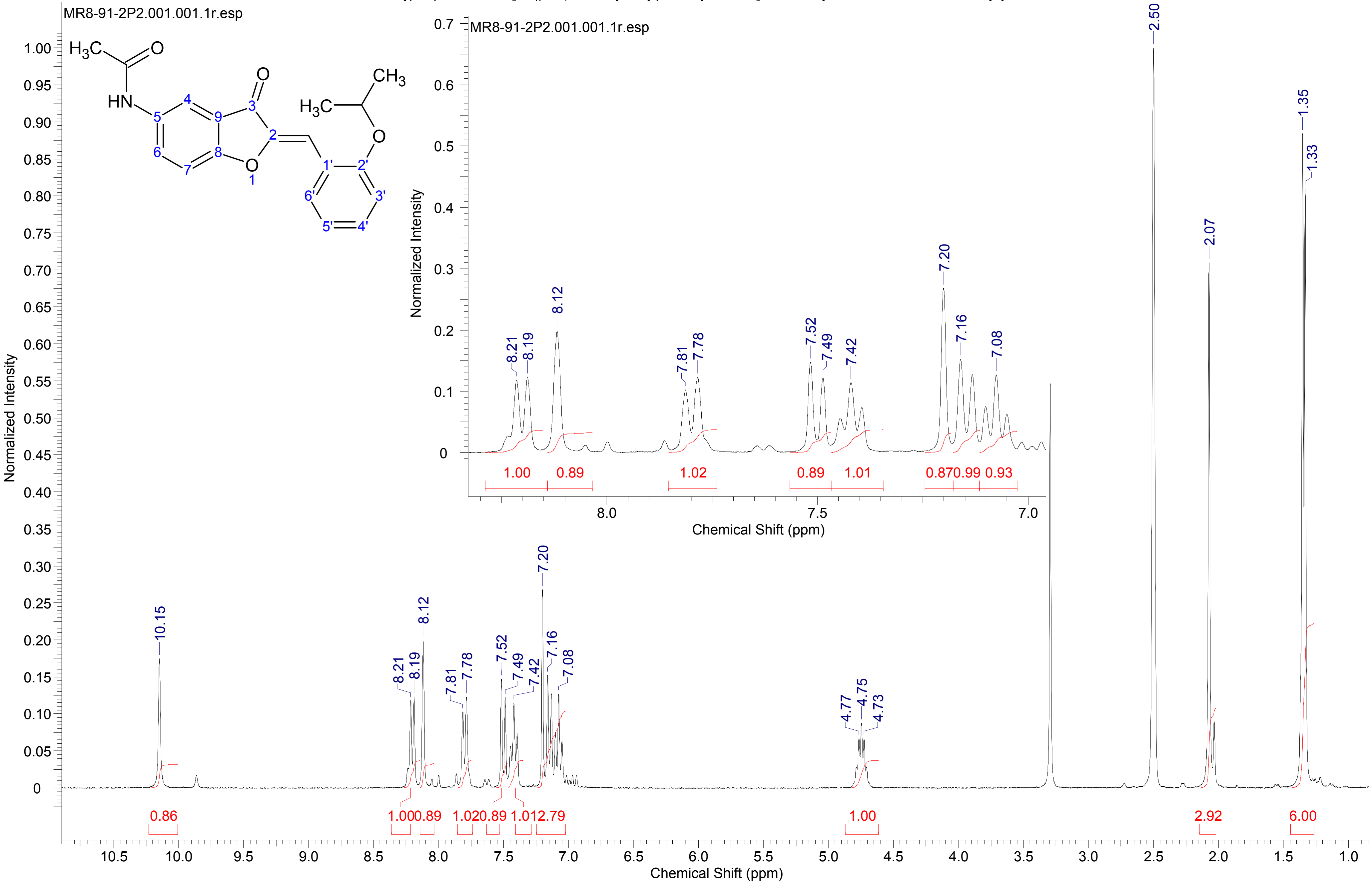

C:\Documents and Settings\RMN\2018\MR8-91-2P2\MR8-91-2P2.001.001.1r.esp

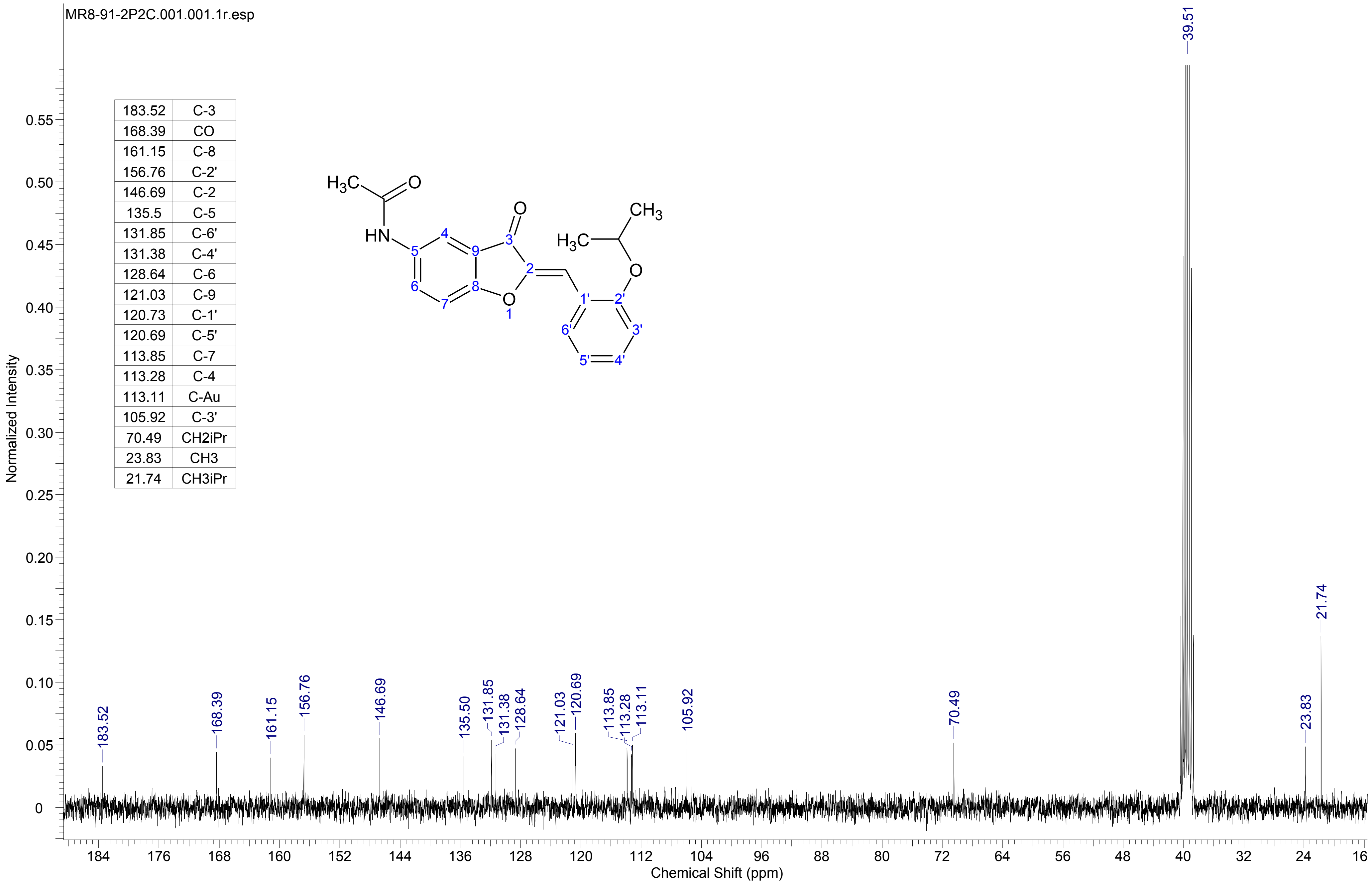

183.52	C-3
168.39	CO
161.15	C-8
156.76	C-2'
146.69	C-2
135.5	C-5
131.85	C-6'
131.38	C-4'
128.64	C-6
121.03	C-9
120.73	C-1'
120.69	C-5'
113.85	C-7
113.28	C-4
113.11	C-Au
105.92	C-3'
70.49	CH2iPr
23.83	CH3
21.74	CH3iPr

C:\Documents and Settings\RMN\2018\MR8-91-2P2C\MR8-91-2P2C.001.001.1r.esp

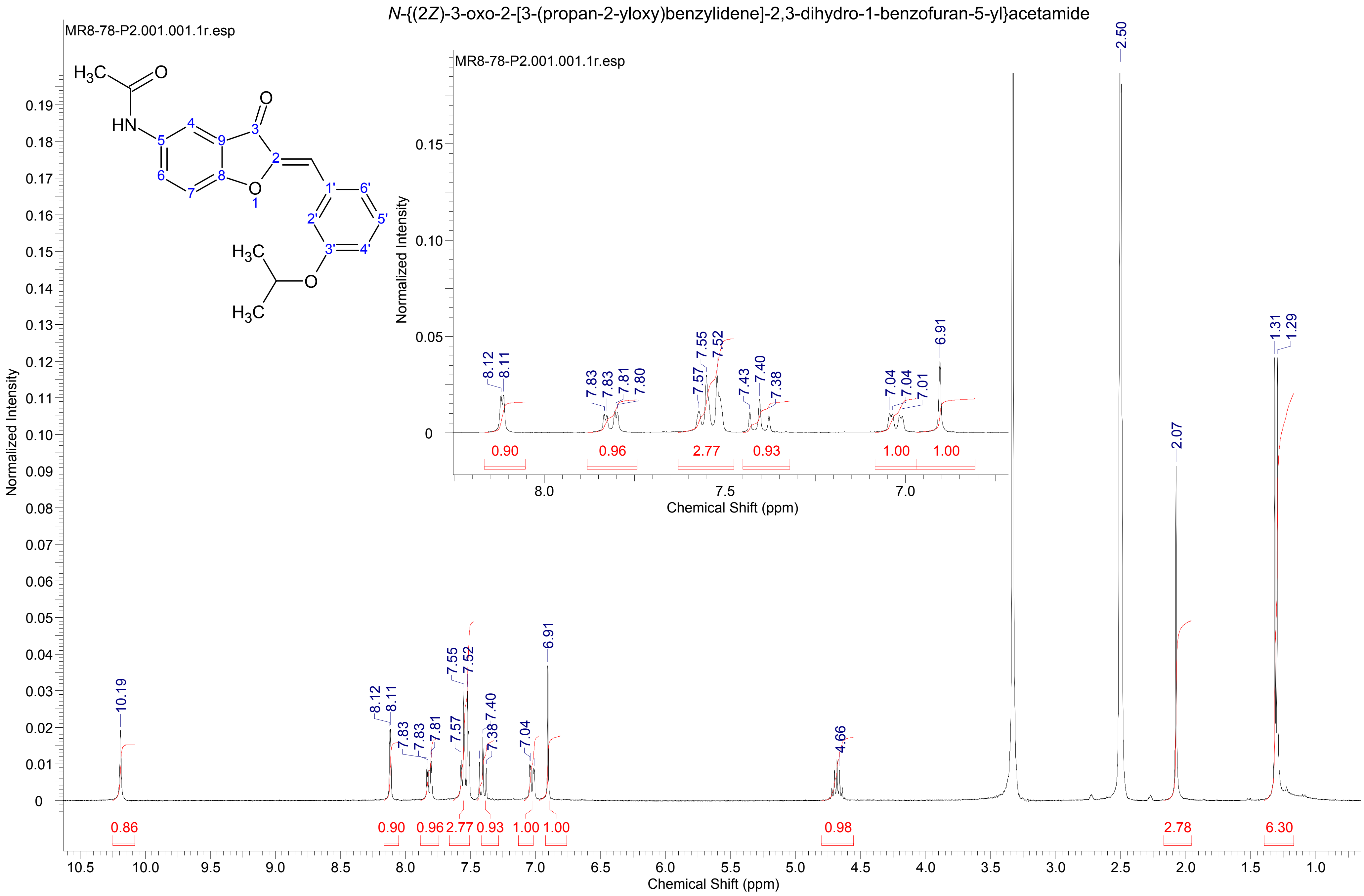

C:\Documents and Settings\RMN\2017\MR8-78-P2\MR8-78-P2.001.001.1r.esp

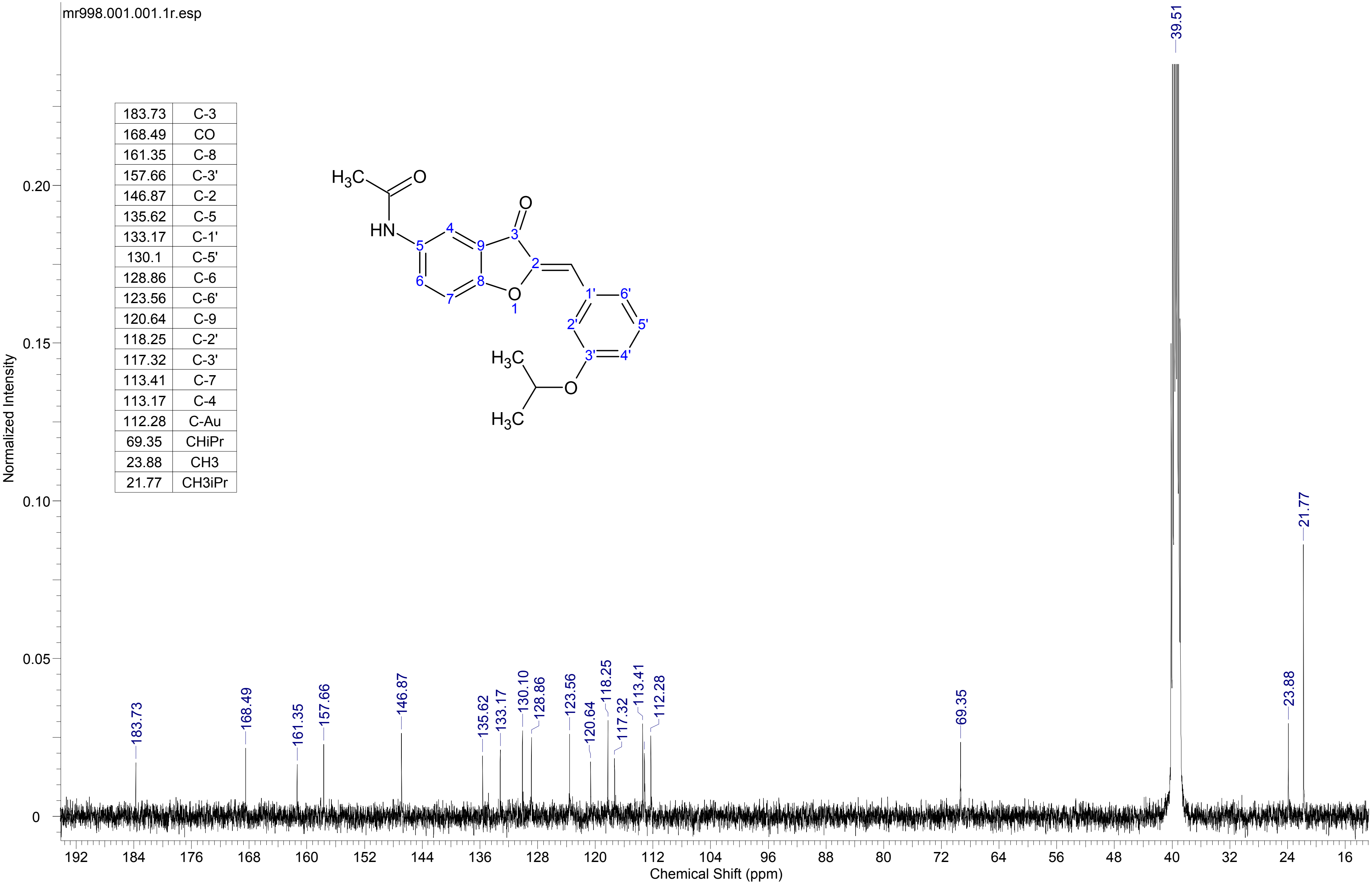

183.73	C-3
168.49	CO
161.35	C-8
157.66	C-3'
146.87	C-2
135.62	C-5
133.17	C-1'
130.1	C-5'
128.86	C-6
123.56	C-6'
120.64	C-9
118.25	C-2'
117.32	C-3'
113.41	C-7
113.17	C-4
112.28	C-Au
69.35	CHiPr
23.88	CH3
21.77	CH3iPr

C:\Documents and Settings\RMN\2017\mr998\mr998.001.001.1r.esp

N-{(2*Z*)-3-oxo-2-[4-(propan-2-yloxy)benzylidene]-2,3-dihydro-1-benzofuran-5-yl}acetamide

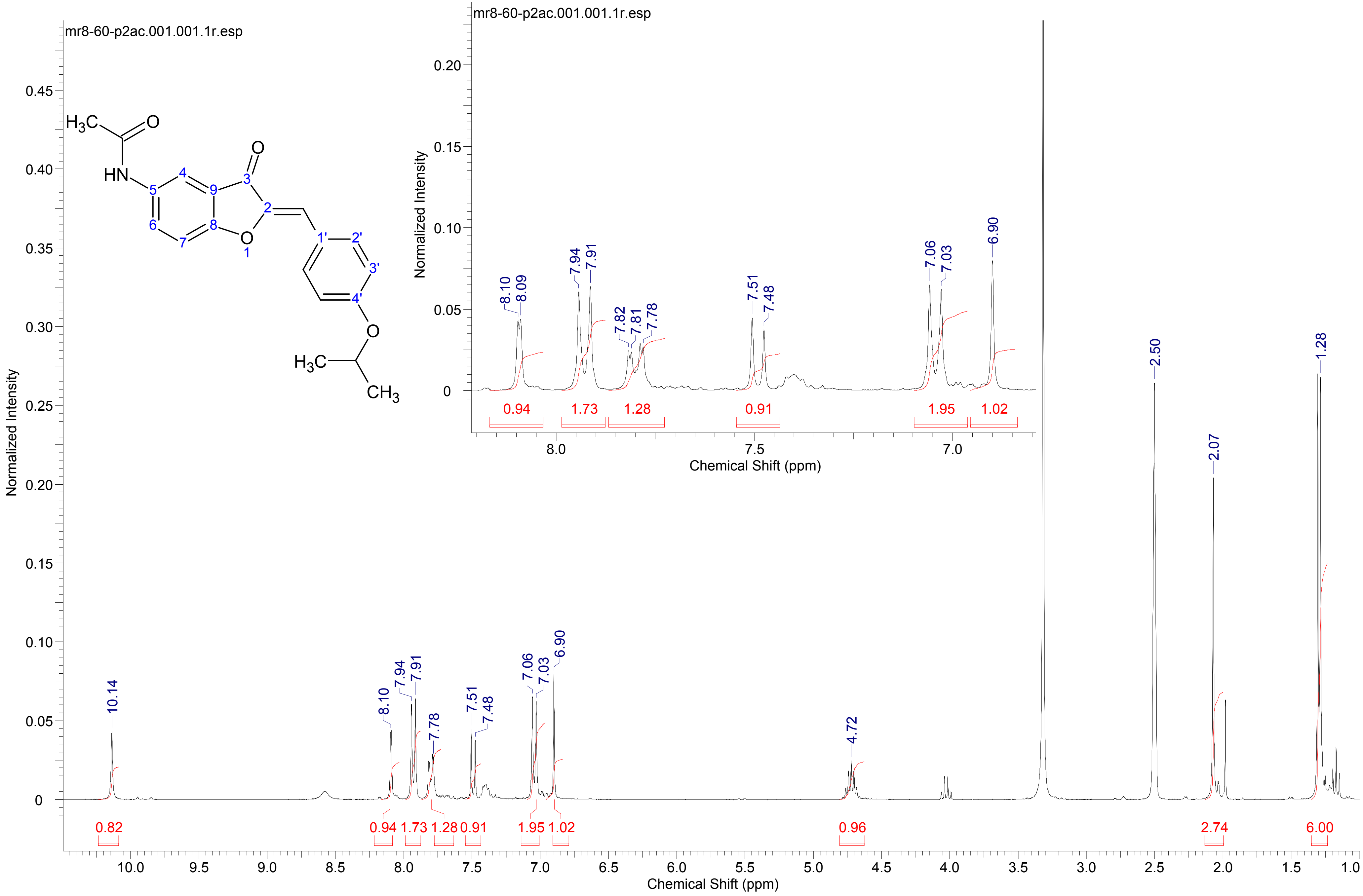

C:\Documents and Settings\RMN\2017\mr8-60-p2ac\mr8-60-p2ac.001.001.1r.esp

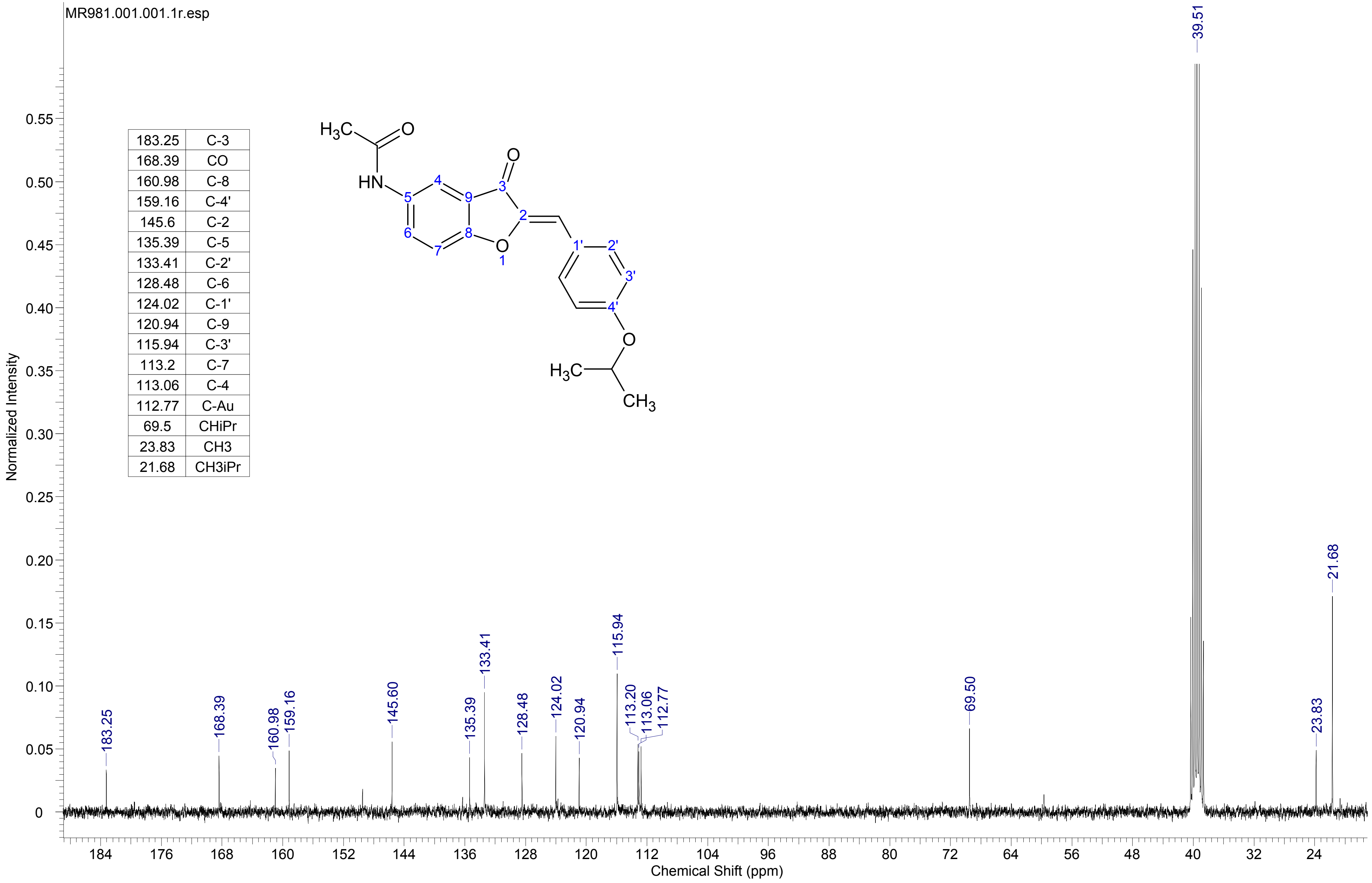

183.25	C-3
168.39	CO
160.98	C-8
159.16	C-4'
145.6	C-2
135.39	C-5
133.41	C-2'
128.48	C-6
124.02	C-1'
120.94	C-9
115.94	C-3'
113.2	C-7
113.06	C-4
112.77	C-Au
69.5	CHiPr
23.83	CH3
21.68	CH3iPr

C:\Documents and Settings\RMN\2017\MR981\MR981.001.001.1r.esp

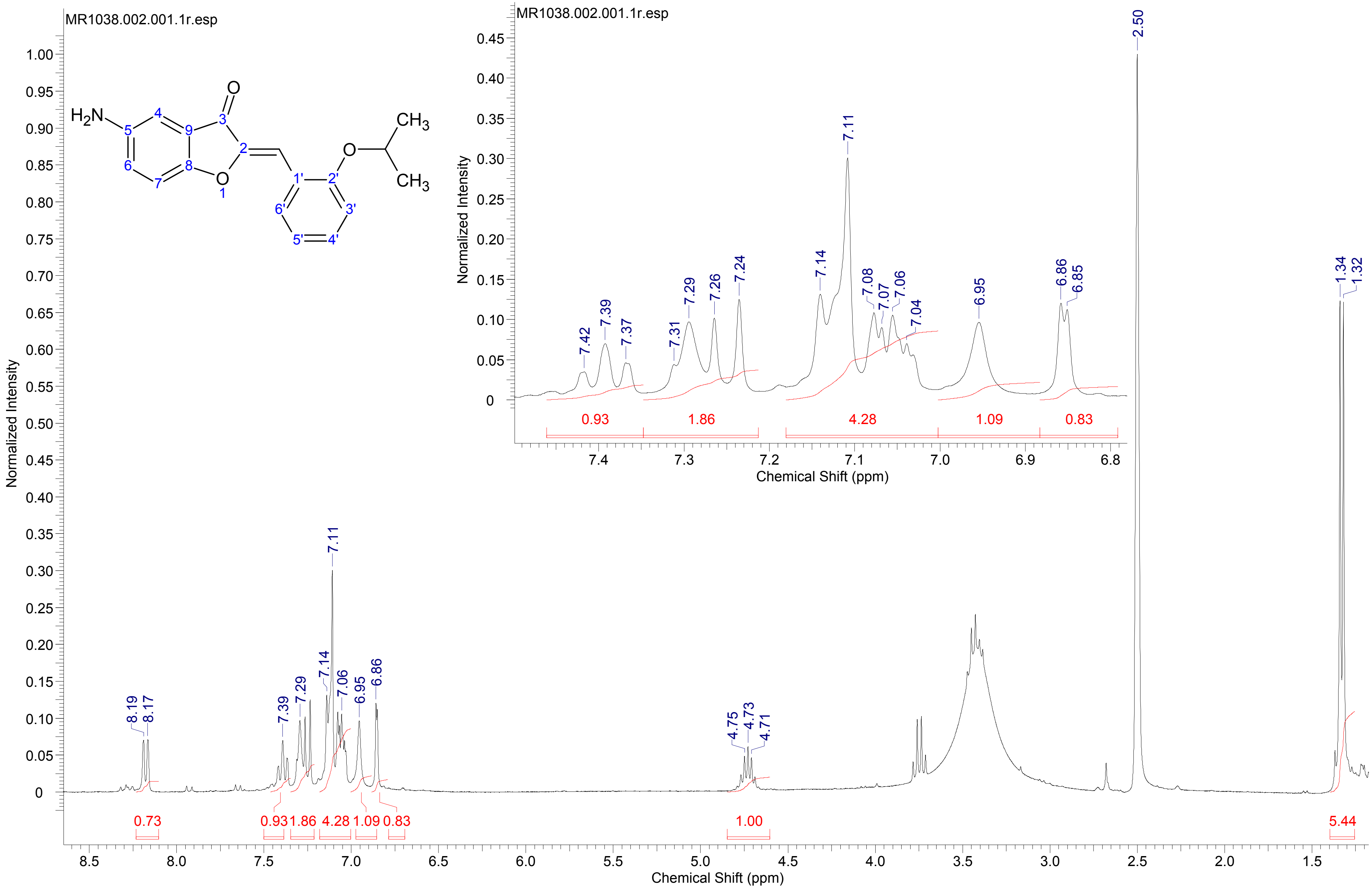

C:\Documents and Settings\RMN\2019\MR1038\MR1038.002.001.1r.esp

MR1038.001.001.1r.esp

184.16	CO
158.14	C-8
156.62	C-2'
147.02	C-2
145.24	C-5
131.55	C-6'
131.33	C-4'
124.76	C-6
121.4	C-9
121.11	C-5'
120.72	C-1'
113.85	C-7
113.23	C-Au
105.77	C-3'
104.8	C-4
70.46	CH
21.82	CH3

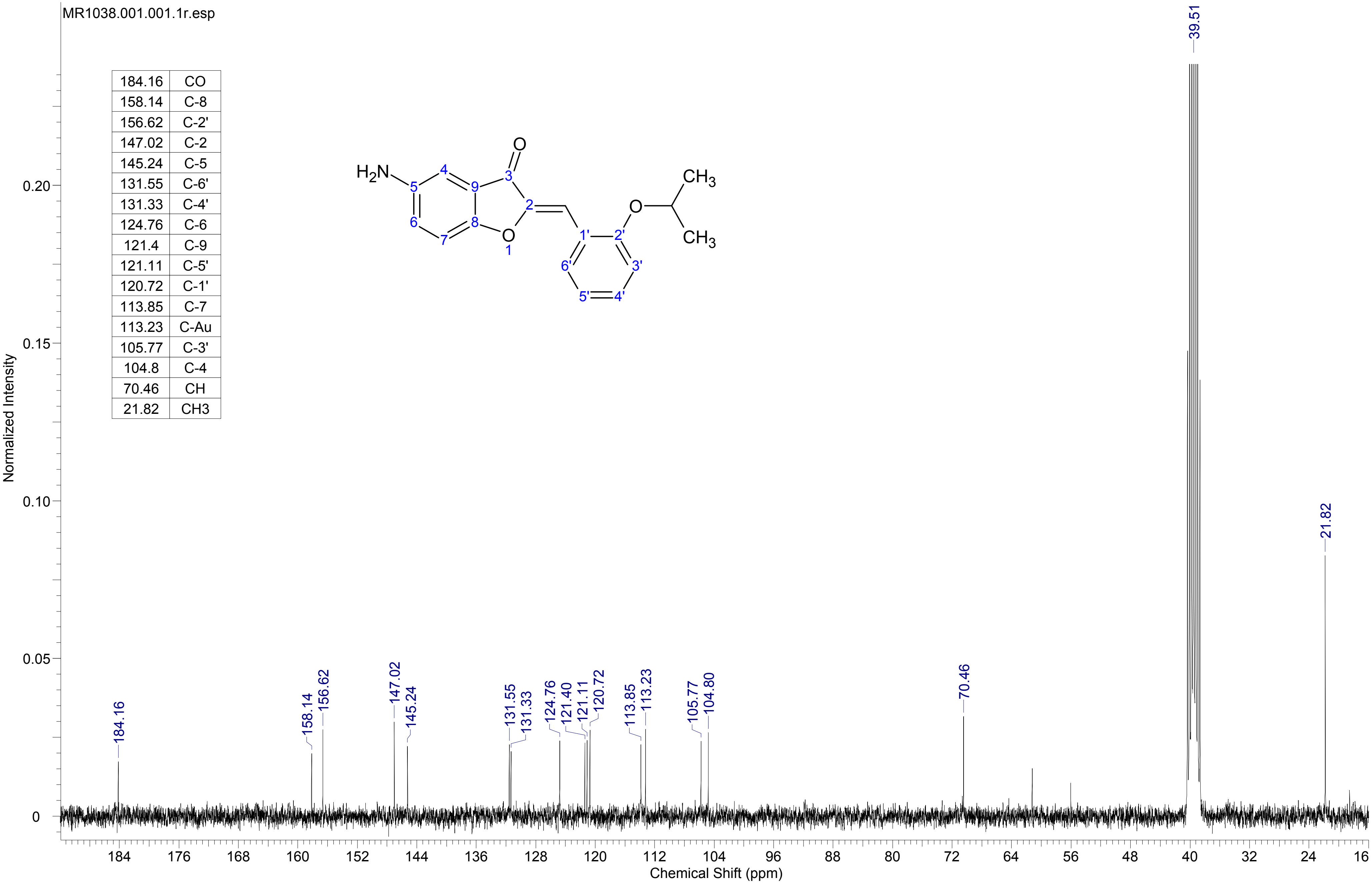

C:\Documents and Settings\RMN\2019\MR1038\MR1038.001.001.1r.esp

(2Z)-5-amino-2-[3-(propan-2-yloxy)benzylidene]-1-benzofuran-3(2H)-one

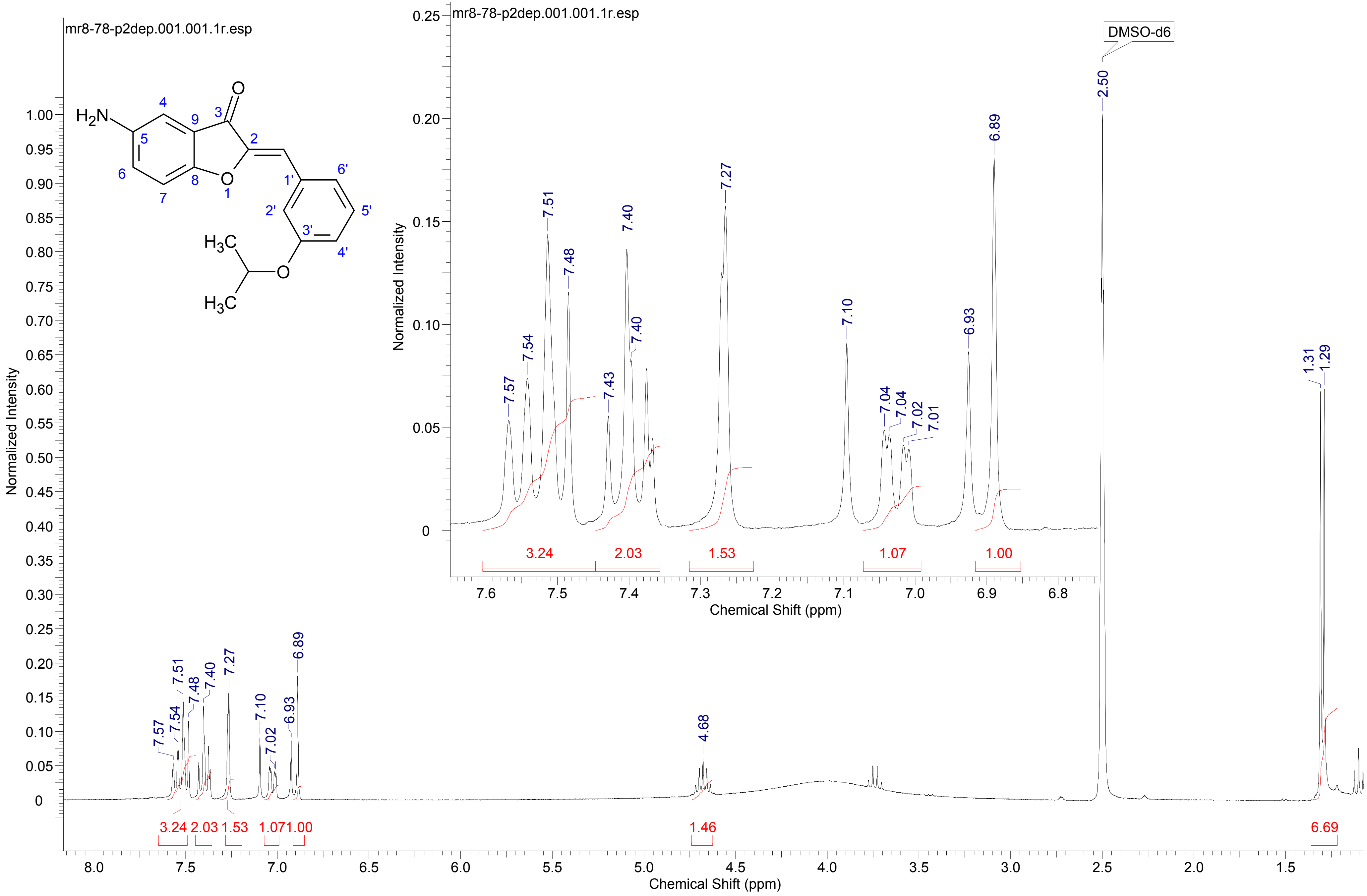

C:\Users\max\Documents\RMN\MR8-2017\mr8-78-p2dep\mr8-78-p2dep.001.001.1r.esp

MR1001C.001.001.1r.esp

183.65	C-3
160.93	C-8
157.67	C-3'
146.95	C-2
137.64	C-5
133.24	C-1'
130.11	C-5'
128.17	C-6
123.56	C-6'
121.25	C-9
118.24	C-4
117.28	C-7
113.96	C-2'
112.14	C-3'
111.46	C-Au
69.35	CHiPr
21.79	CH3iPr

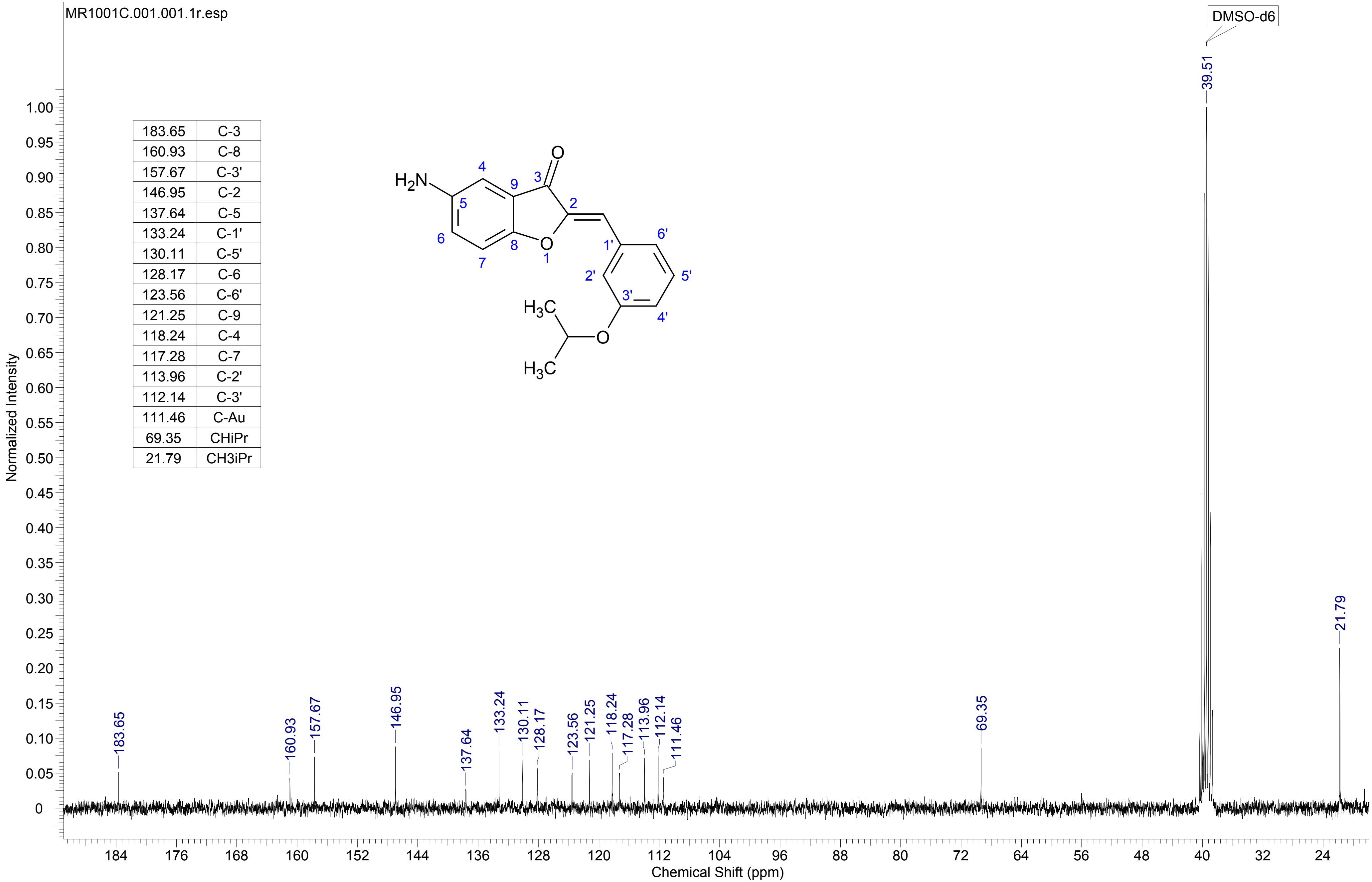

C:\Users\max\Documents\RMN\MR8-2017\MR1001C\MR1001C.001.001.1r.esp

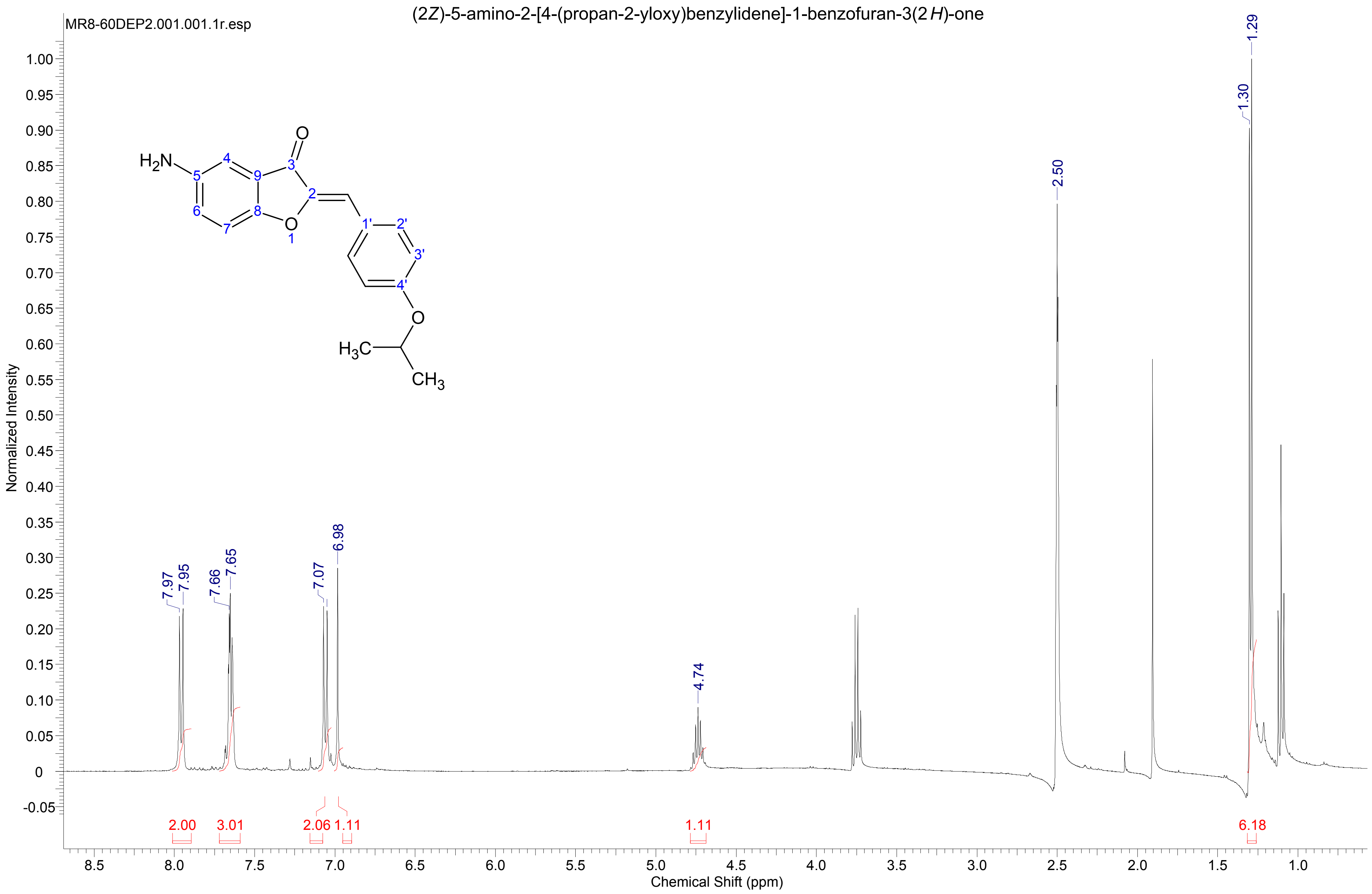

C:\Documents and Settings\RMN\2017\MR8-60DEP2\MR8-60DEP2.001.001.1r.esp

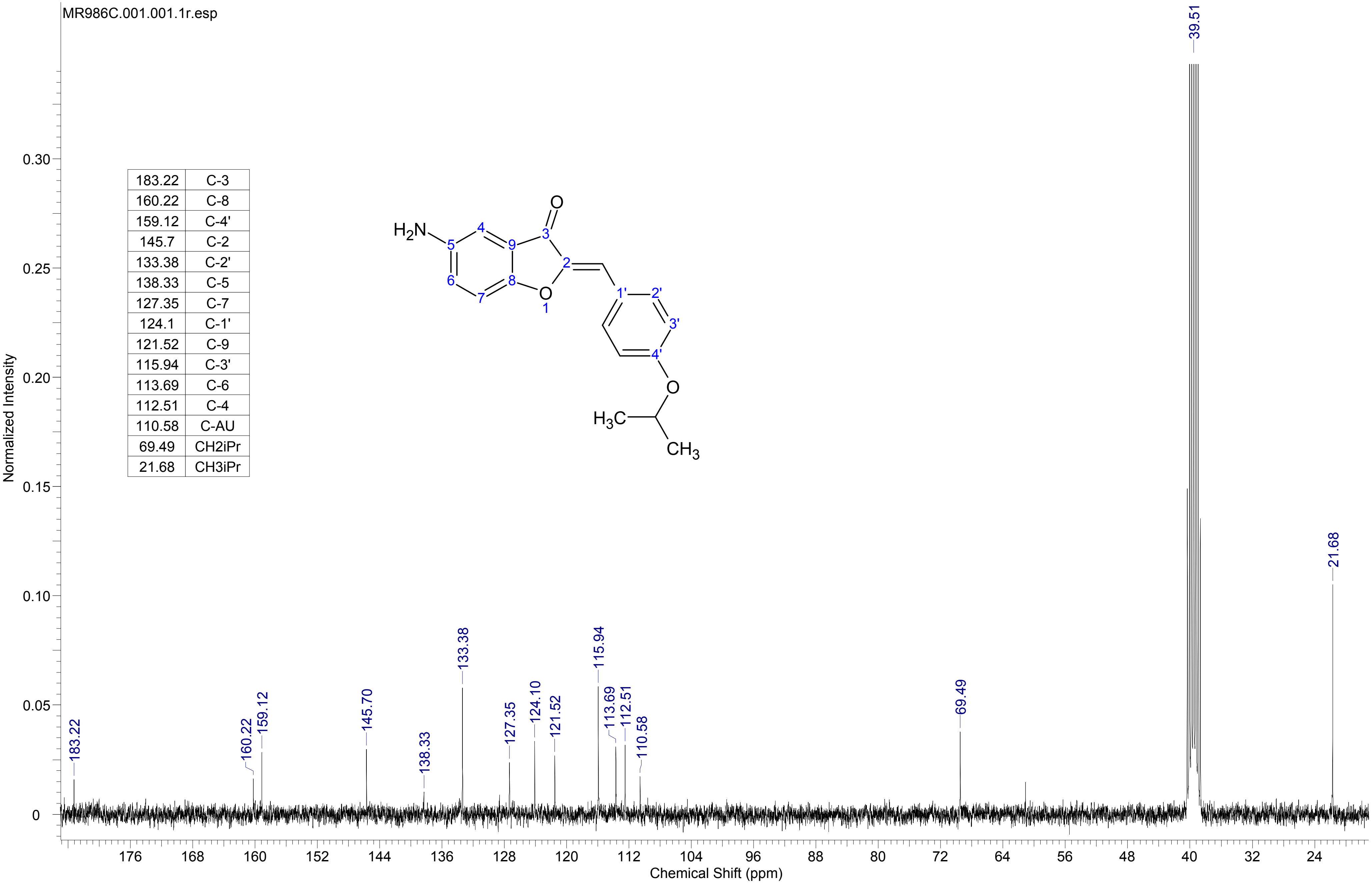

183.22	C-3
160.22	C-8
159.12	C-4'
145.7	C-2
133.38	C-2'
138.33	C-5
127.35	C-7
124.1	C-1'
121.52	C-9
115.94	C-3'
113.69	C-6
112.51	C-4
110.58	C-AU
69.49	CH2iPr
21.68	CH3iPr

C:\Documents and Settings\RMN\2017\MR986C\MR986C.001.001.1r.esp

N-[(2Z)-2-(2-fluorobenzylidene)-3-oxo-2,3-dihydro-1-benzofuran-5-yl]acetamide

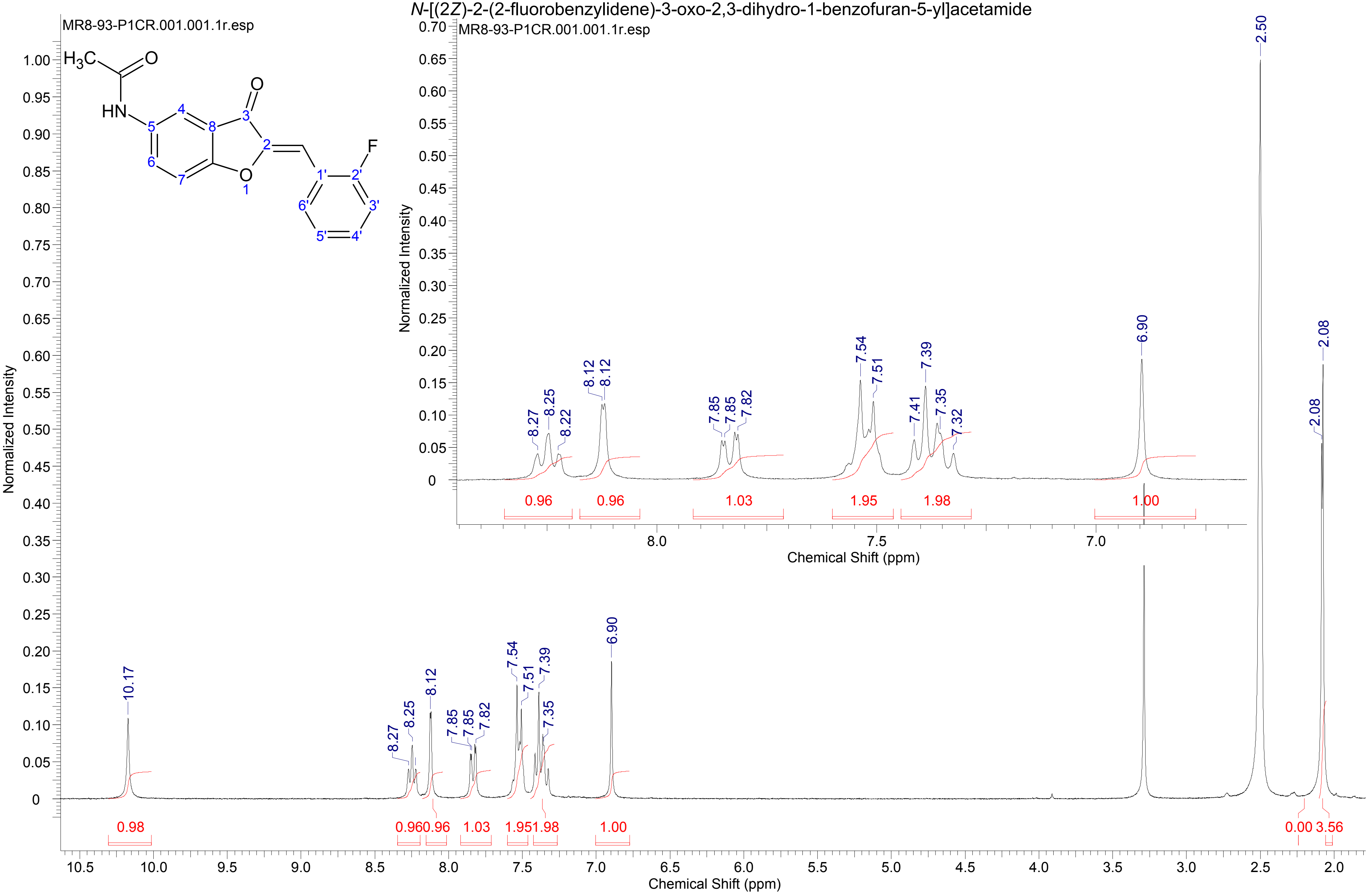

C:\Documents and Settings\RMN\2018\MR8-93-P1CR\MR8-93-P1CR.001.001.1r.esp

MR8-93-P1CRC.001.001.1r.esp

183.5	C-3	
168.44	CO	
164.9	C-2'	J=260Hz
161.44	C-2'	
161.41	C-8	
147.71	C-2	
135.78	C-5	
132.24	C-4'	J= 8.8 Hz
132.12	C-4'	
131.31	C-6'	
129.02	C-6	
125.11	C-5'	J=3.3 Hz
120.38	C-9	
119.67	C-1'	J= 11 Hz
119.52	C-1'	
115.93	C-3'	J= 22 Hz
115.64	C-3'	
113.38	C-7	
113.25	C-4	
102.1	C-Au	J=7.7 Hz
102	C-Au	
23.83	CH3	

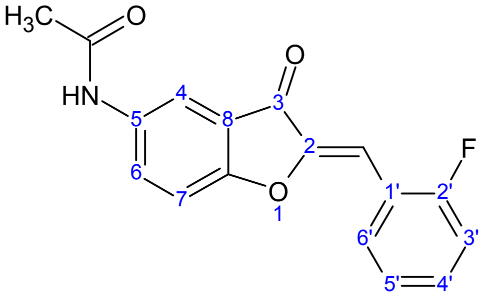

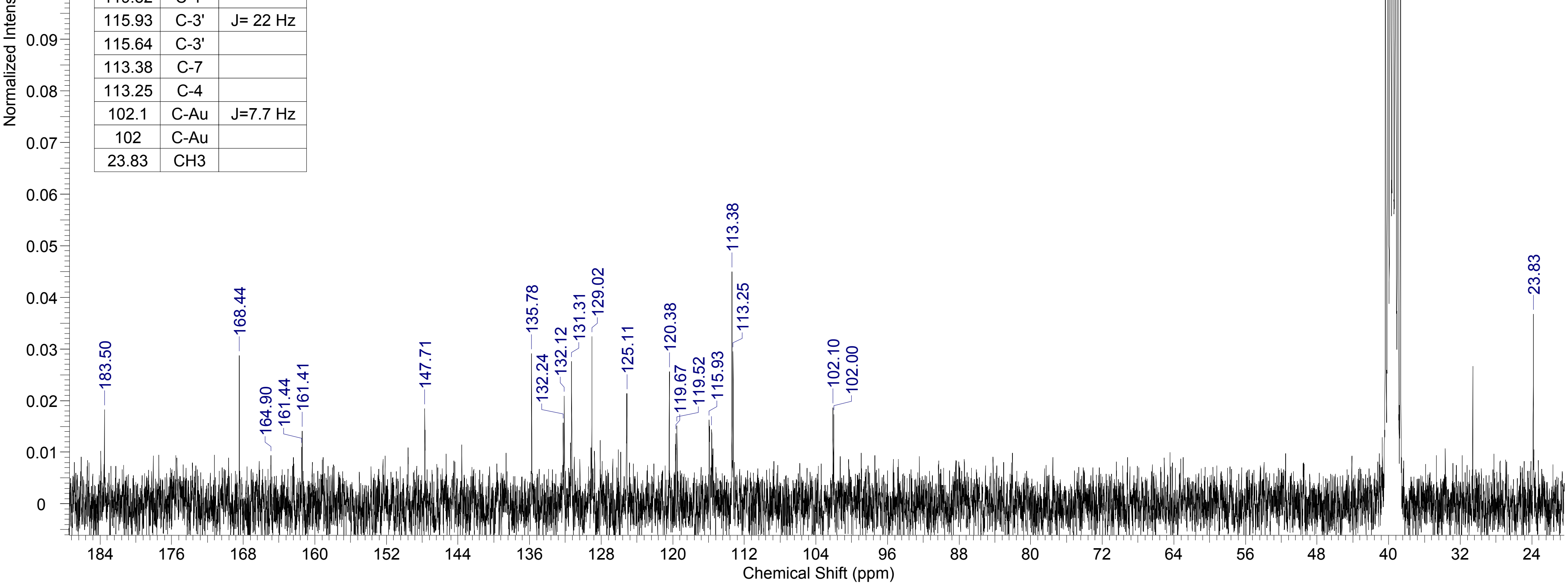

C:\Documents and Settings\RMN\2018\MR8-93-P1CRC\MR8-93-P1CRC.001.001.1r.esp

N-[(2*Z*)-2-(3-fluorobenzylidene)-3-oxo-2,3-dihydro-1-benzofuran-5-yl]acetamide

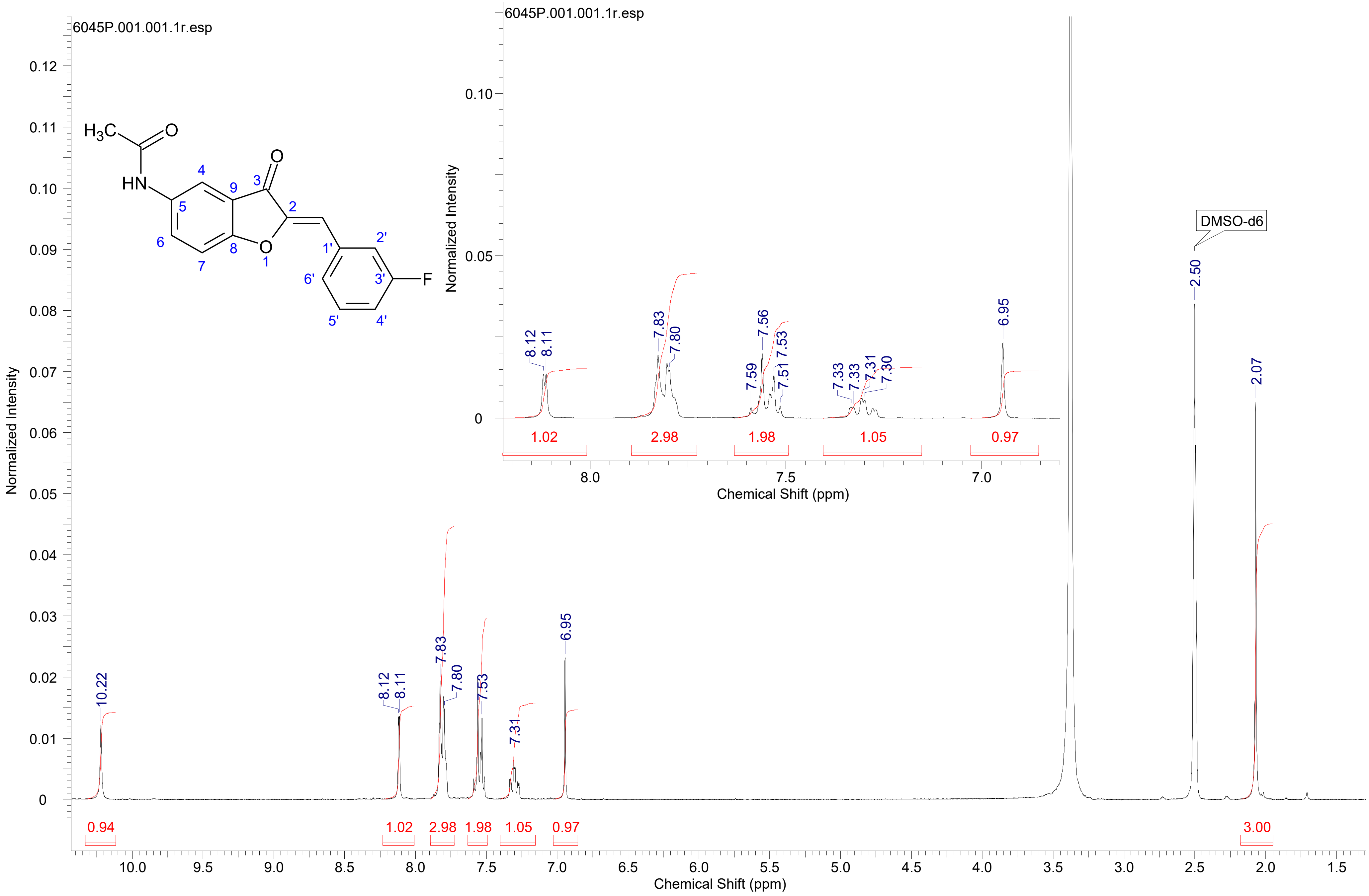

C:\Users\max\Documents\RMN\MR6\6045P\6045P.001.001.1r.esp

6045PC.001.001.1r.esp

	183.83	C-3
	168.60	CO
	163.85	C-3'
	161.48	C-8
	160.62	C-3'
	147.37	C-2
	135.77	C-5
	134.30	C-1'
	134.19	C-1'
*	131.08	C-5'
*	130.97	C-5'
*	129.05	C-6
*	127.58	C-6'
	120.53	C-9
*	117.45	
*	117.15	
*	117.02	
*	116.73	
*	113.53	CH
*	113.25	C-7
*	110.69	C-2'
*	110.66	C-2'
	23.92	CH3

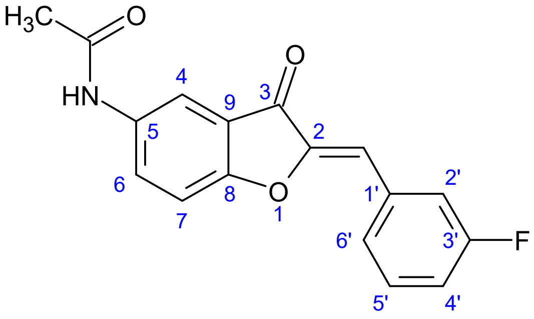

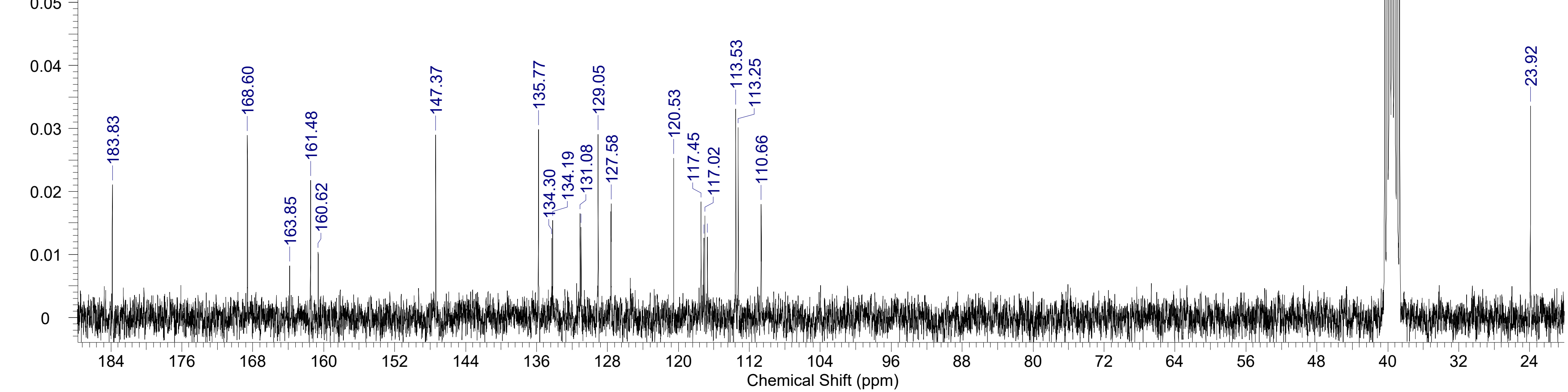

C:\Users\max\Documents\RMN\MR6\6045PC\6045PC.001.001.1r.esp

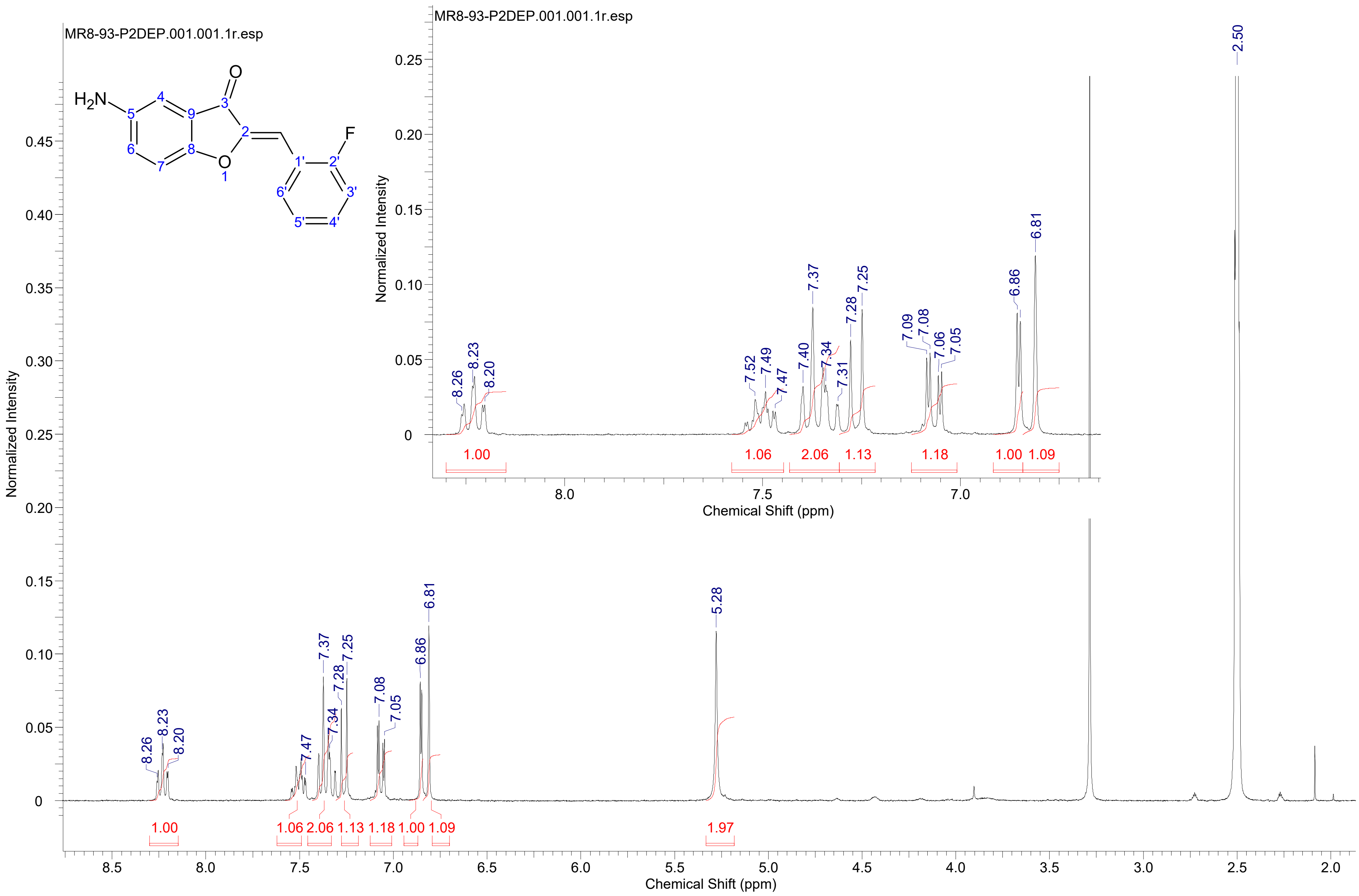

C:\Documents and Settings\RMN\2018\MR8-93-P2DEP\MR8-93-P2DEP.001.001.1r.esp

MR1016.001.001.1r.esp

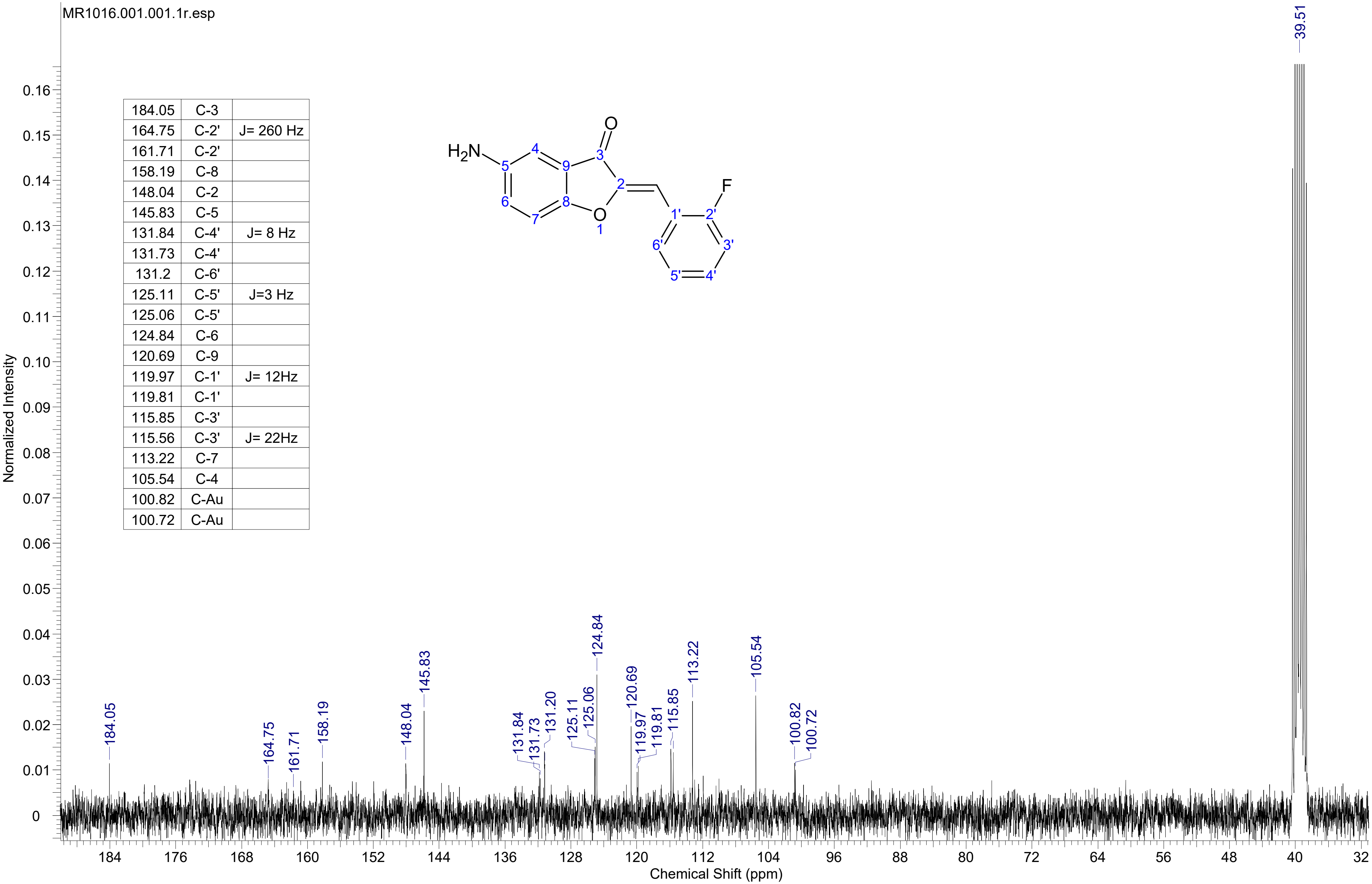

184.05	C-3	
164.75	C-2'	J= 260 Hz
161.71	C-2'	
158.19	C-8	
148.04	C-2	
145.83	C-5	
131.84	C-4'	J= 8 Hz
131.73	C-4'	
131.2	C-6'	
125.11	C-5'	J=3 Hz
125.06	C-5'	
124.84	C-6	
120.69	C-9	
119.97	C-1'	J= 12Hz
119.81	C-1'	
115.85	C-3'	
115.56	C-3'	J= 22Hz
113.22	C-7	
105.54	C-4	
100.82	C-Au	
100.72	C-Au	

C:\Documents and Settings\RMN\2018\MR1016\MR1016.001.001.1r.esp

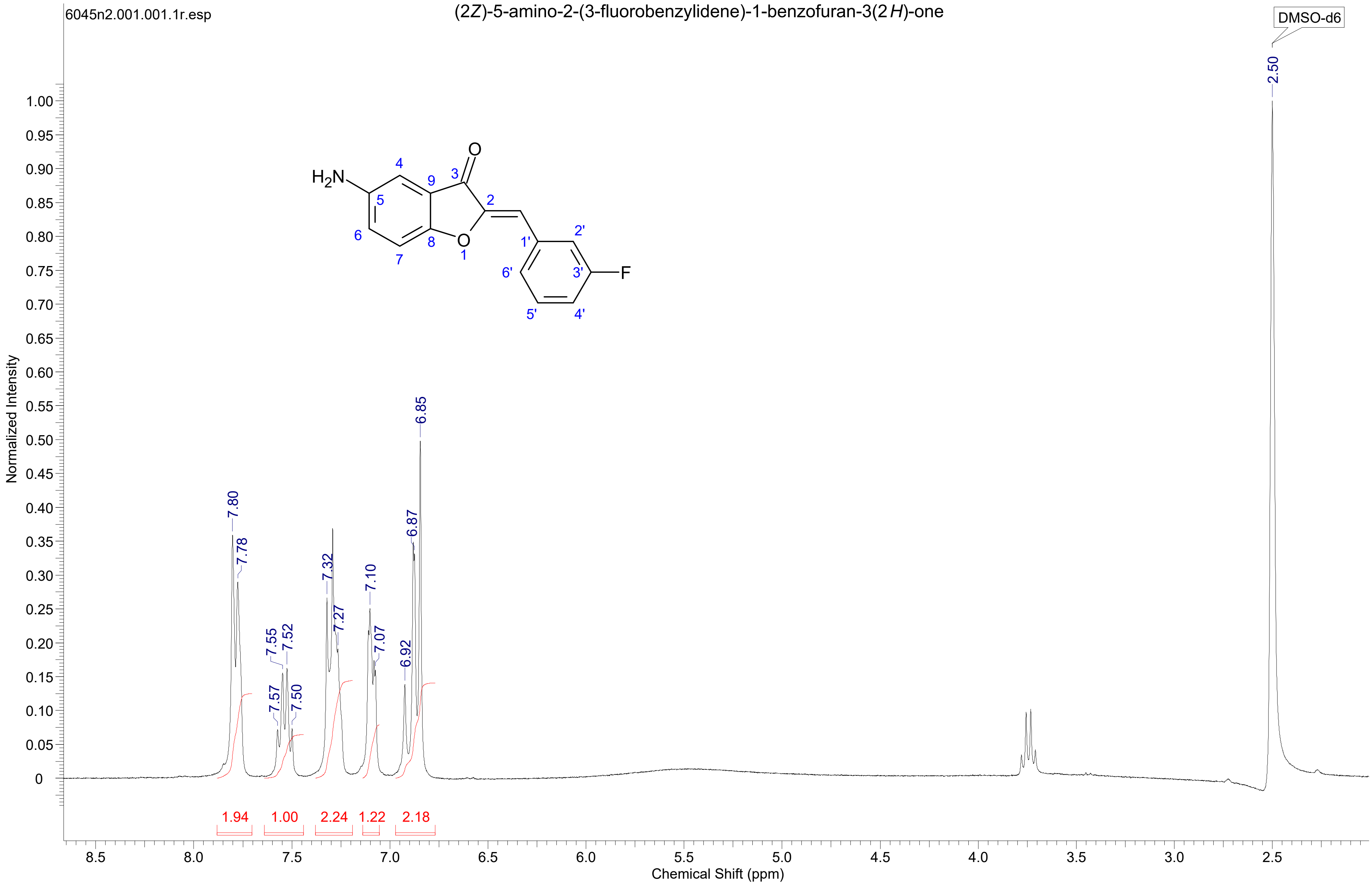

C:\Users\max\Documents\RMN\MR6\6045n2\6045n2.001.001.1r.esp

6045n2c.001.001.1r.esp

184.26	C-3
163.81	C-3'
160.58	C-3'
158.49	C-8
147.63	C-2
145.01	C-5
134.58	C-1'
134.47	C-1'
130.85	C-6
127.34	
125.18	
120.84	C-9
117.22	
116.92	
116.64	
116.36	
113.38	C-H
109.49	
109.45	
106.08	C-7

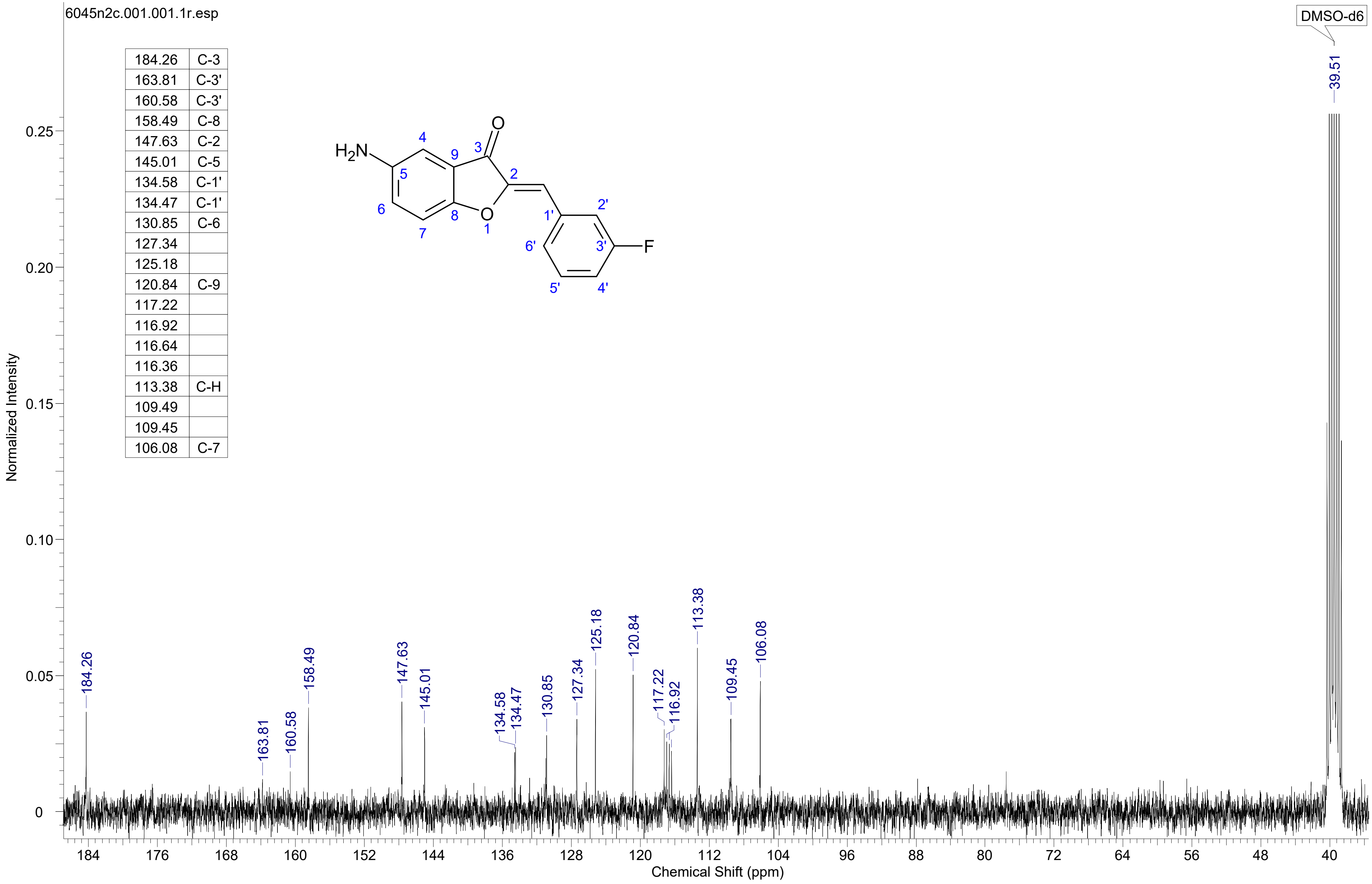

C:\Users\max\Documents\RMN\MR6\6045n2c\6045n2c.001.001.1r.esp

(2Z)-5-amino-2-(4-fluorobenzylidene)-1-benzofuran-3(2H)-one

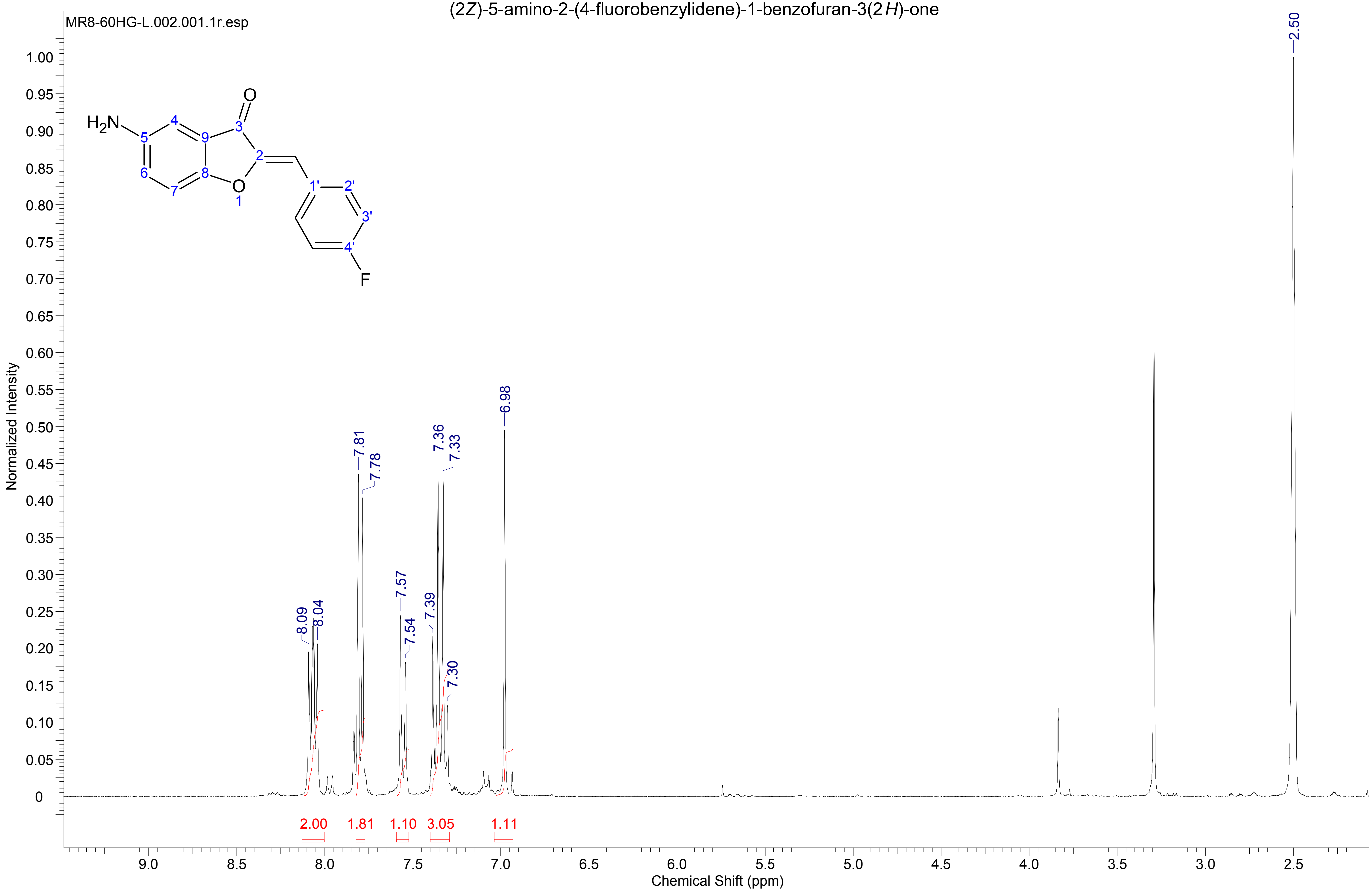

C:\Documents and Settings\RMN\2017\MR8-60HG-L\MR8-60HG-L.002.001.1r.esp

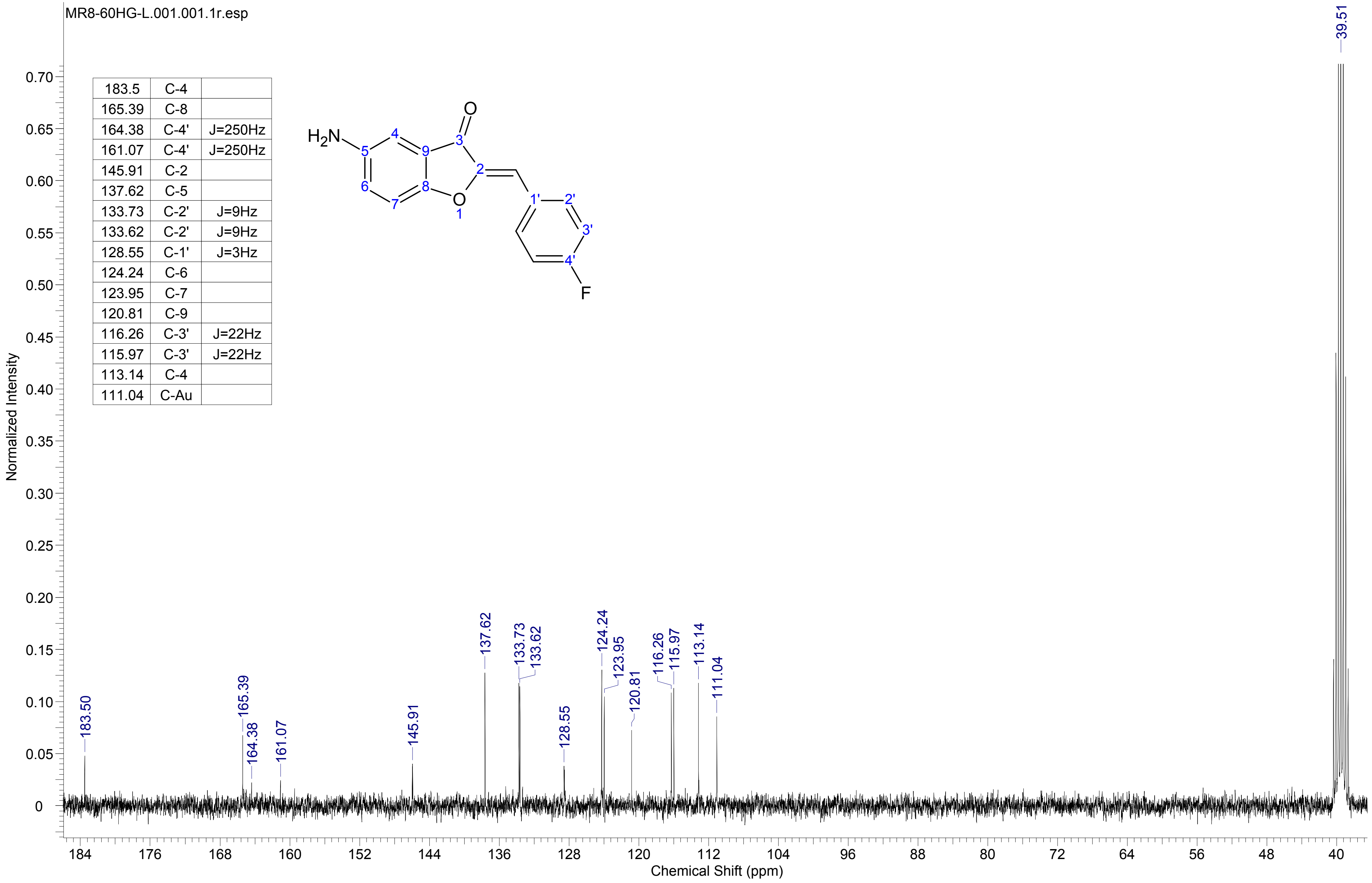

183.5	C-4	
165.39	C-8	
164.38	C-4'	J=250Hz
161.07	C-4'	J=250Hz
145.91	C-2	
137.62	C-5	
133.73	C-2'	J=9Hz
133.62	C-2'	J=9Hz
128.55	C-1'	J=3Hz
124.24	C-6	
123.95	C-7	
120.81	C-9	
116.26	C-3'	J=22Hz
115.97	C-3'	J=22Hz
113.14	C-4	
111.04	C-Au	

C:\Documents and Settings\RMN\2017\MR8-60HG-L\MR8-60HG-L.001.001.1r.esp

N-{(2*Z*)-3-oxo-2-[3-(trifluoromethyl)benzylidene]-2,3-dihydro-1-benzofuran-5-yl}acetamide

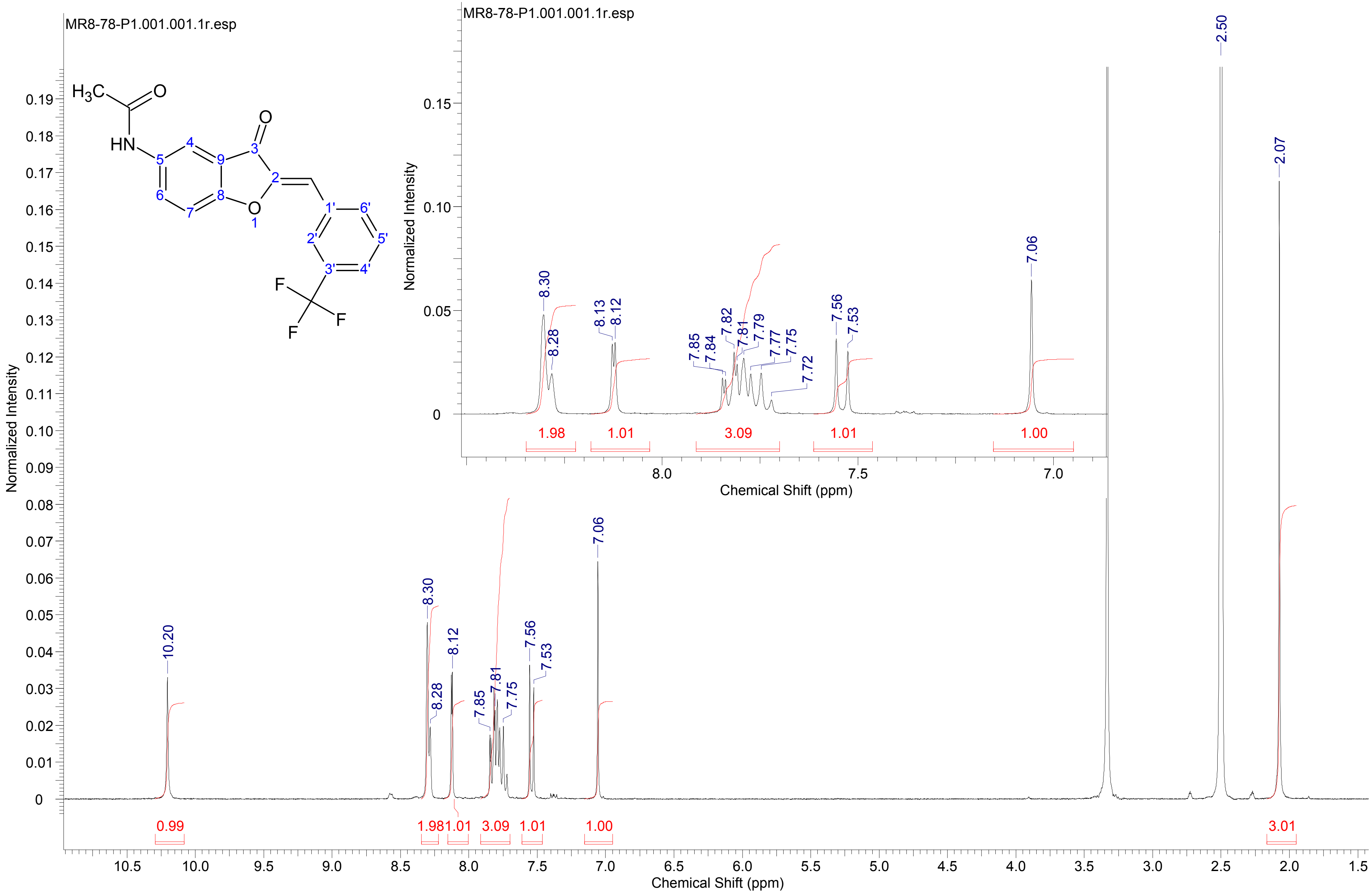

C:\Documents and Settings\RMN\2017\MR8-78-P1\MR8-78-P1.001.001.1r.esp

mr997.001.001.1r.esp

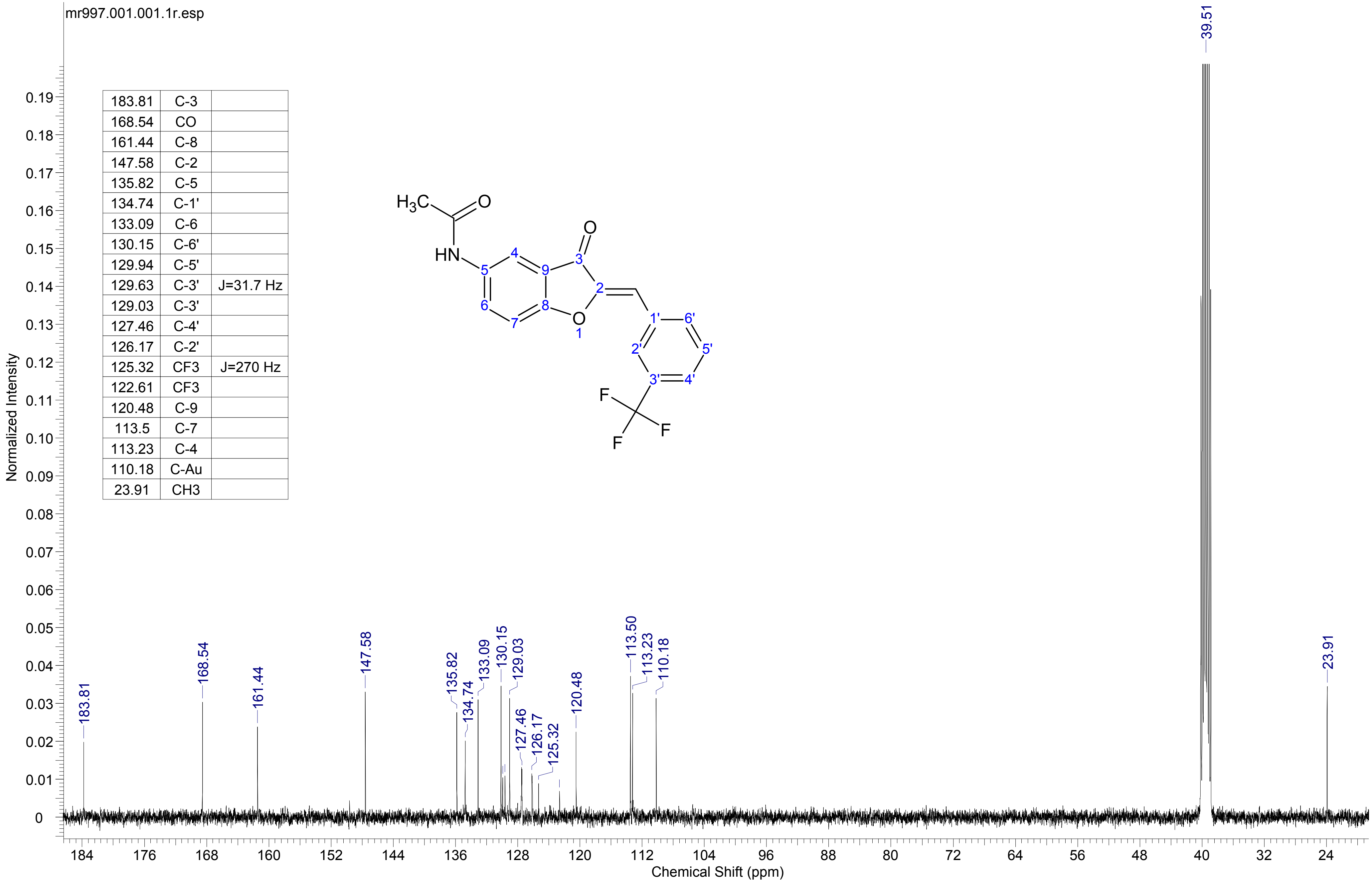

183.81	C-3	
168.54	CO	
161.44	C-8	
147.58	C-2	
135.82	C-5	
134.74	C-1'	
133.09	C-6	
130.15	C-6'	
129.94	C-5'	
129.63	C-3'	J=31.7 Hz
129.03	C-3'	
127.46	C-4'	
126.17	C-2'	
125.32	CF3	J=270 Hz
122.61	CF3	
120.48	C-9	
113.5	C-7	
113.23	C-4	
110.18	C-Au	
23.91	CH3	

C:\Documents and Settings\RMN\2017\mr997\mr997.001.001.1r.esp

(2Z)-5-amino-2-[3-(trifluoromethyl)benzylidene]-1-benzofuran-3(2 H)-one

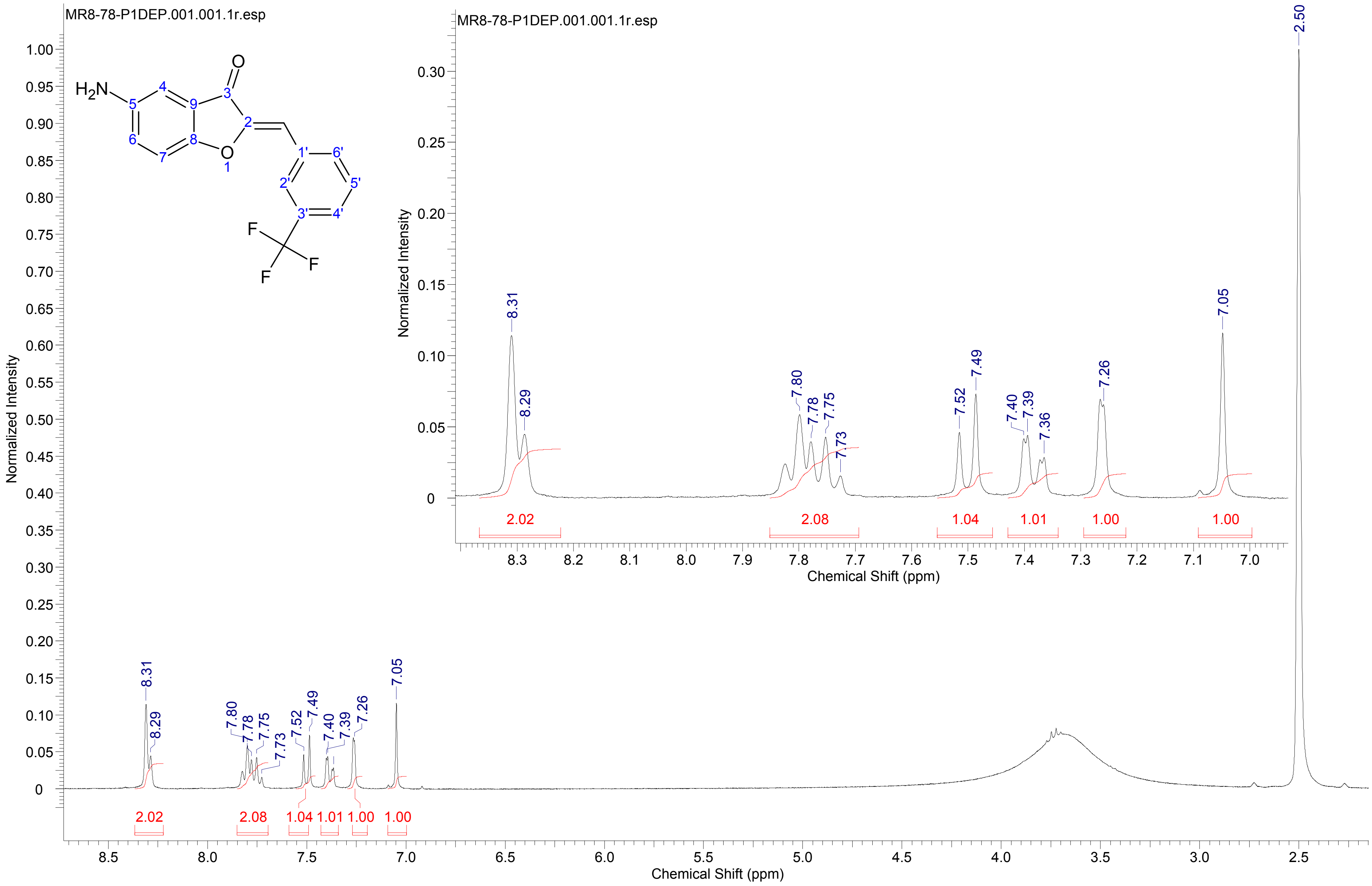

C:\Documents and Settings\RMN\2017\MR8-78-P1DEP\MR8-78-P1DEP.001.001.1r.esp

MR1000C.001.001.1r.esp

183.7	C-3	
161.09	C-8	
147.65	C-2	
134.75	C-1'	
133.13	C-5	
130.16	C-6'	
130.01	C-3'	J=31 Hz
129.59	C-3'	
128.44	C-7	
127.48	C-4'	
127.43	C-2'	
126.11	CF3	J=250Hz
122.78	CF3	
121.1	C-9	
114.05	C-6	
111.68	C-4	
110.05	C-Au	

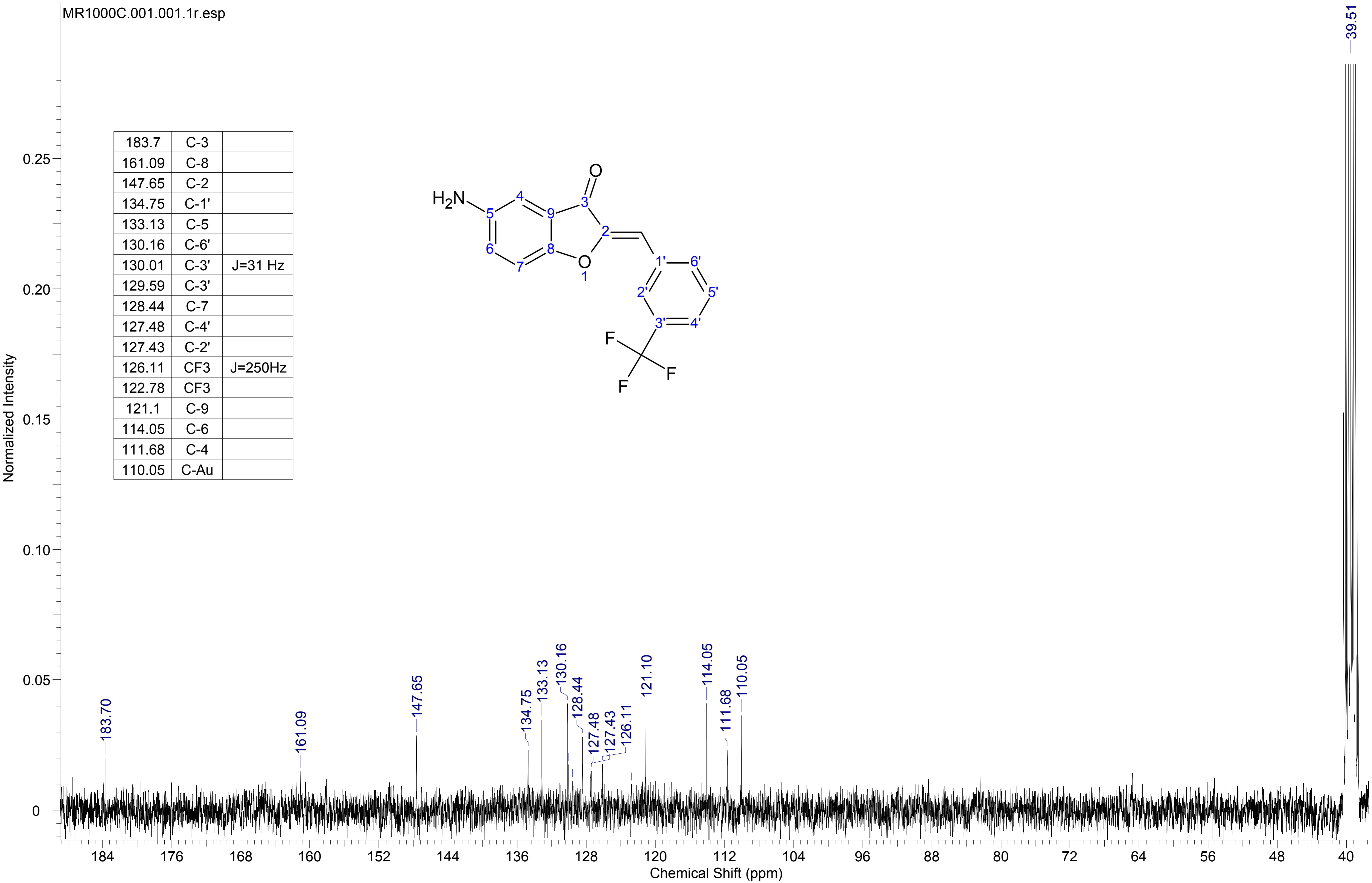

C:\Documents and Settings\RMN\2017\MR1000C\MR1000C.001.001.1r.esp

4-{(Z)-[5-(acetylamino)-3-oxo-1-benzofuran-2(3H)-ylidene]methyl}benzoic acid

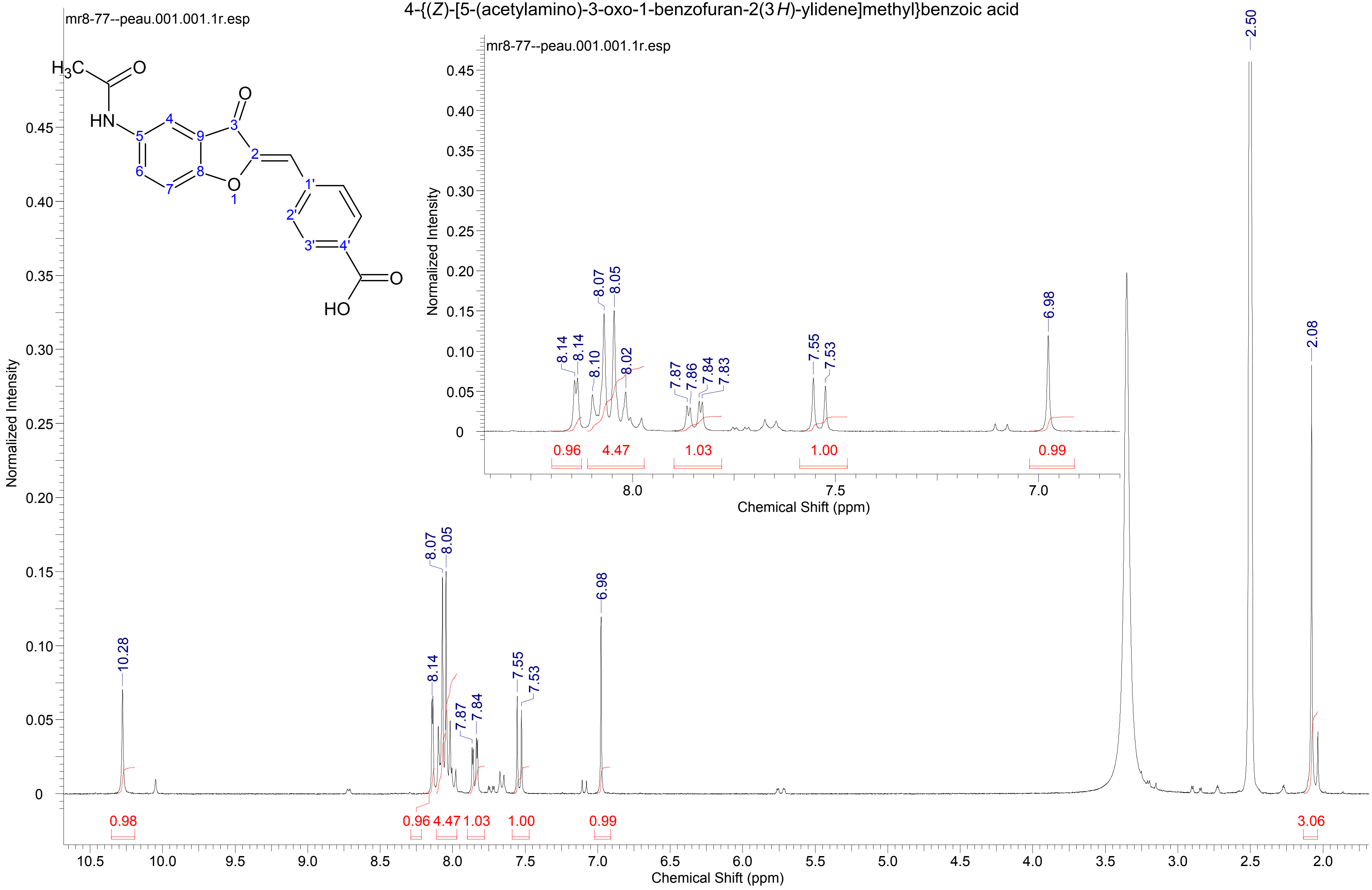

C:\Documents and Settings\RMN\2017\mr8-77--peau\mr8-77--peau.001.001.1r.esp

mr996.001.001.1r.esp

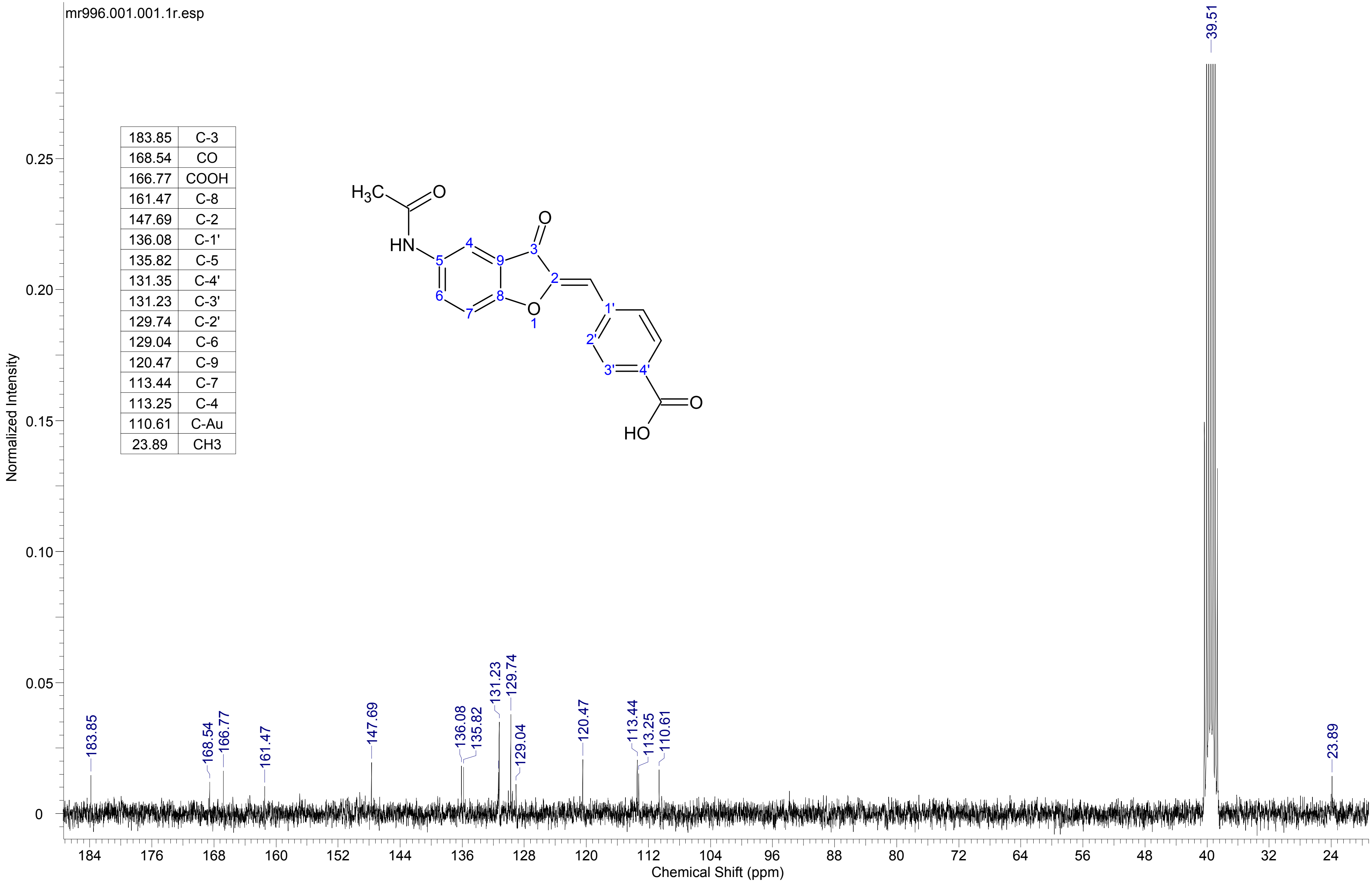

183.85	C-3
168.54	CO
166.77	COOH
161.47	C-8
147.69	C-2
136.08	C-1'
135.82	C-5
131.35	C-4'
131.23	C-3'
129.74	C-2'
129.04	C-6
120.47	C-9
113.44	C-7
113.25	C-4
110.61	C-Au
23.89	CH3

C:\Documents and Settings\RMN\2017\mr996\mr996.001.001.1r.esp

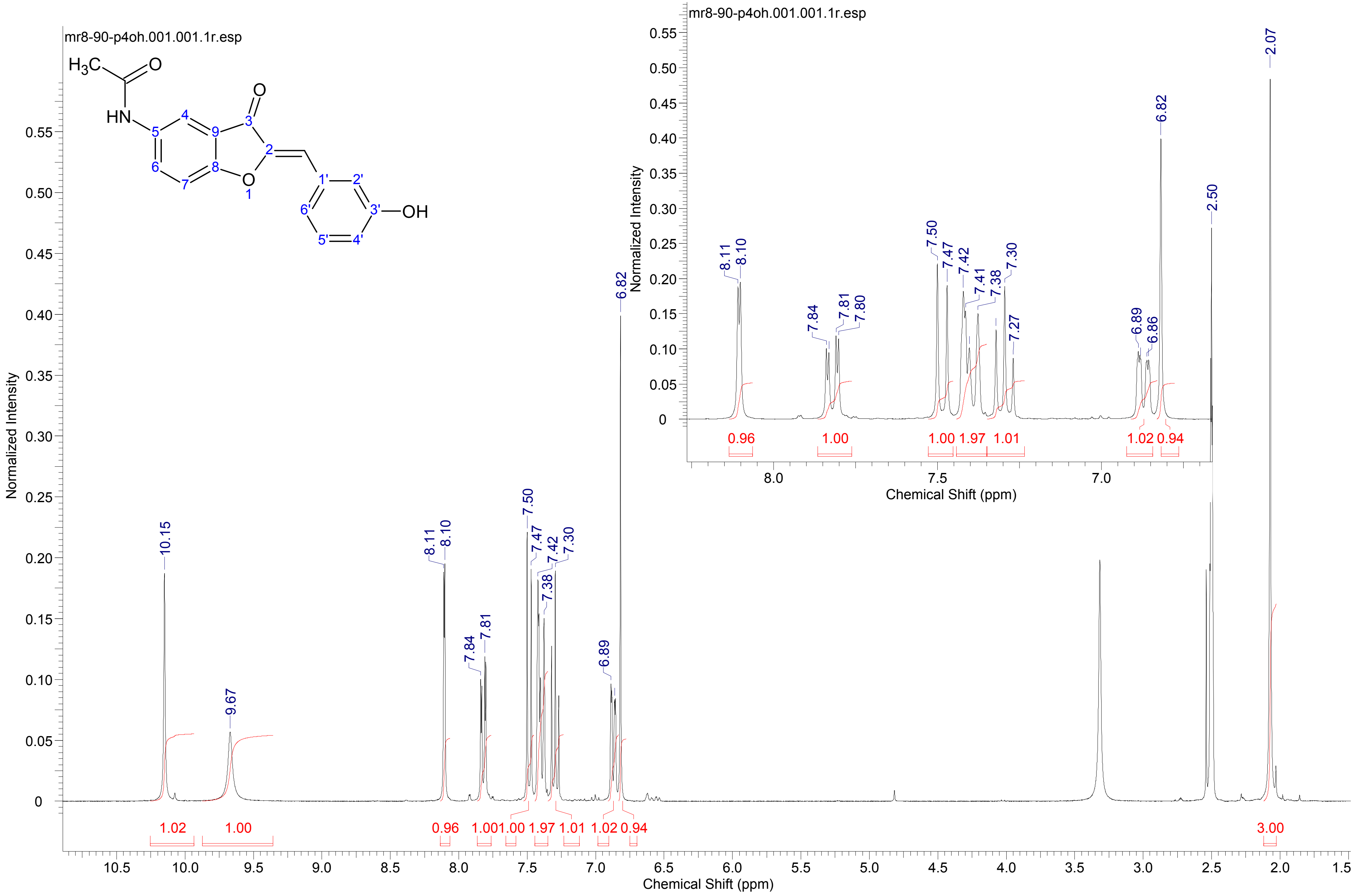

C:\Documents and Settings\RMN\2018\mr8-90-p4oh\mr8-90-p4oh.001.001.1r.esp

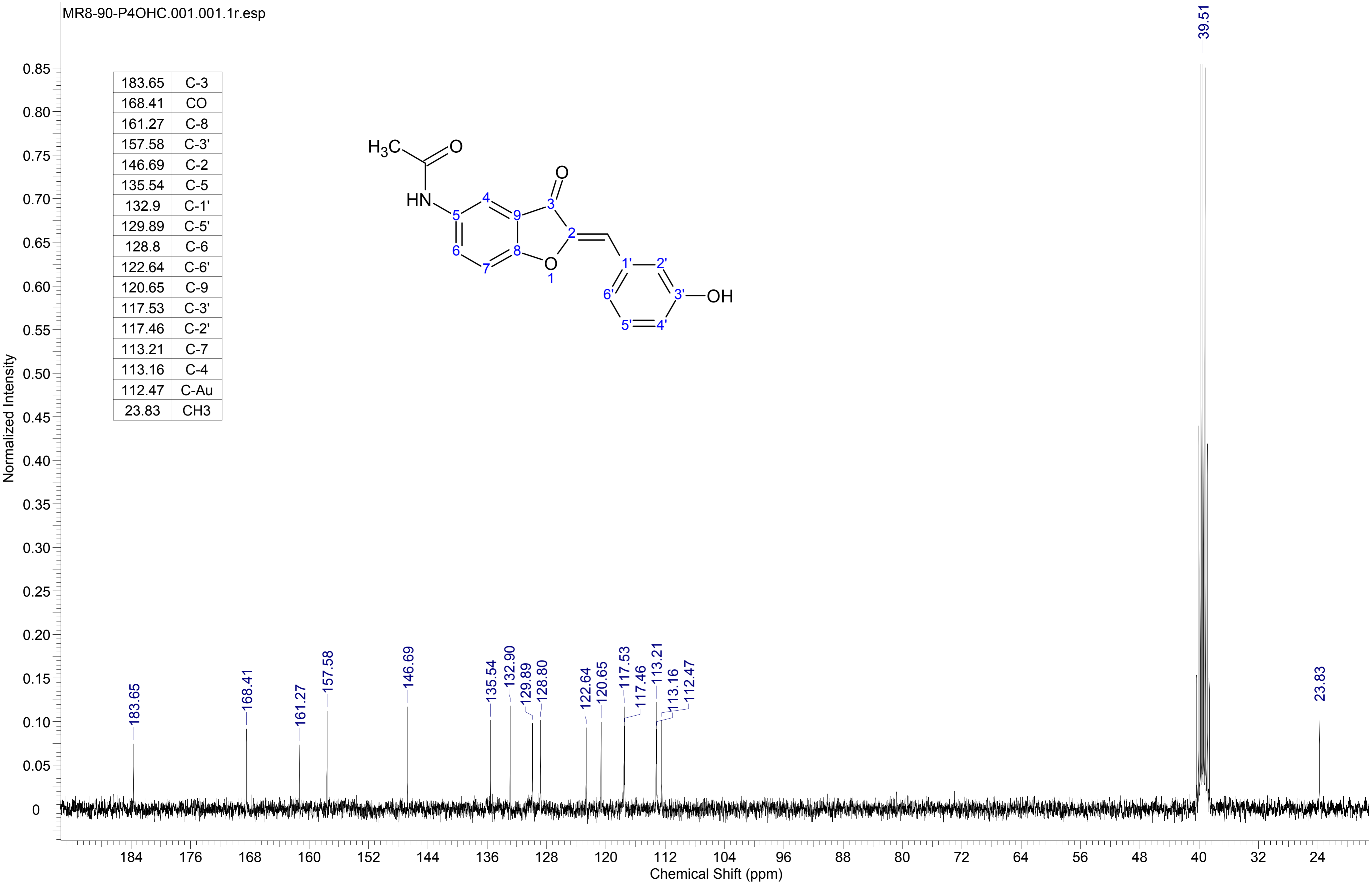

183.65	C-3
168.41	CO
161.27	C-8
157.58	C-3'
146.69	C-2
135.54	C-5
132.9	C-1'
129.89	C-5'
128.8	C-6
122.64	C-6'
120.65	C-9
117.53	C-3'
117.46	C-2'
113.21	C-7
113.16	C-4
112.47	C-Au
23.83	CH3

C:\Documents and Settings\RMN\2018\MR8-90-P4OHC\MR8-90-P4OHC.001.001.1r.esp

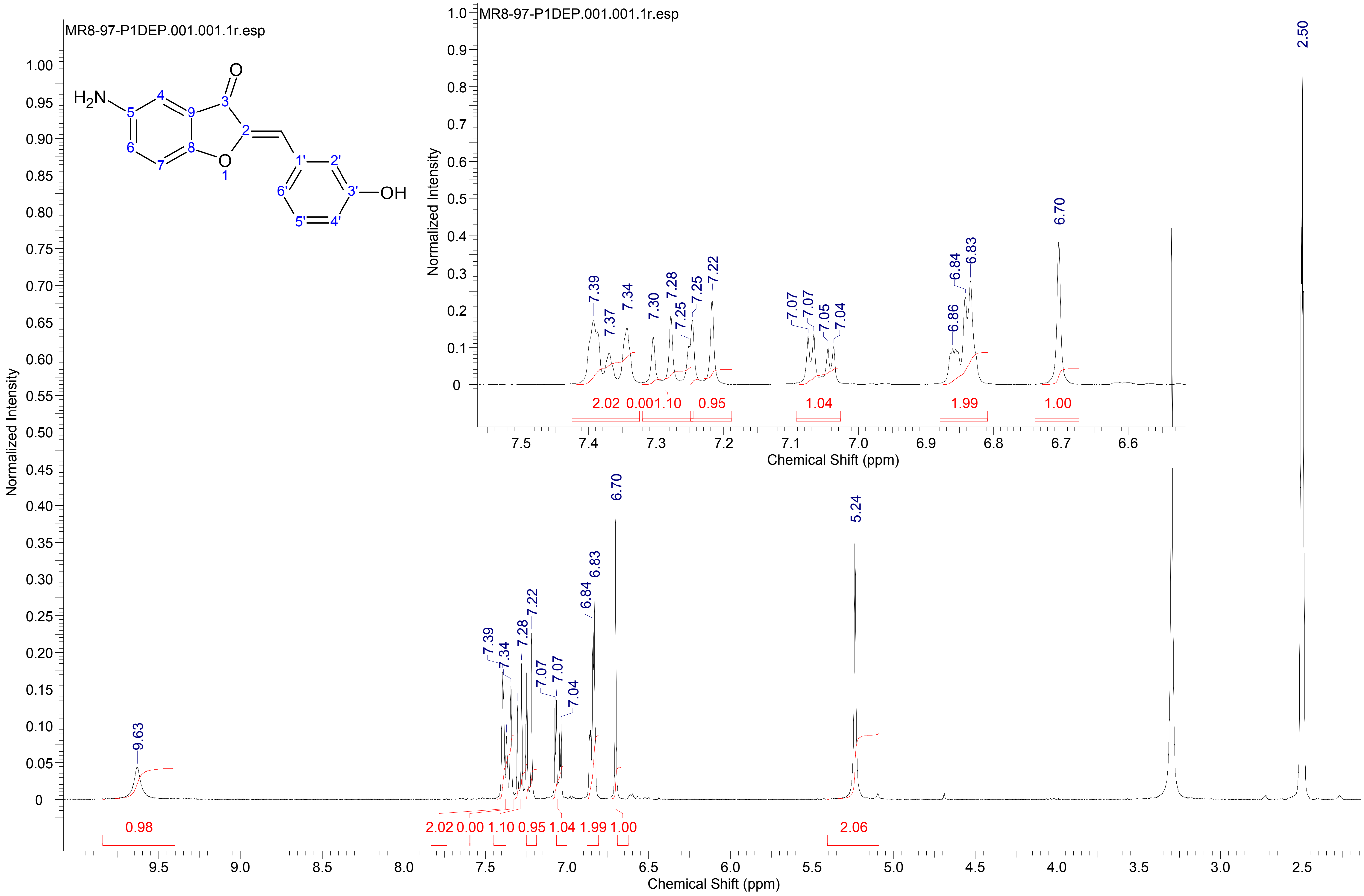

C:\Documents and Settings\RMN\2018\MR8-97-P1DEP\MR8-97-P1DEP.001.001.1r.esp

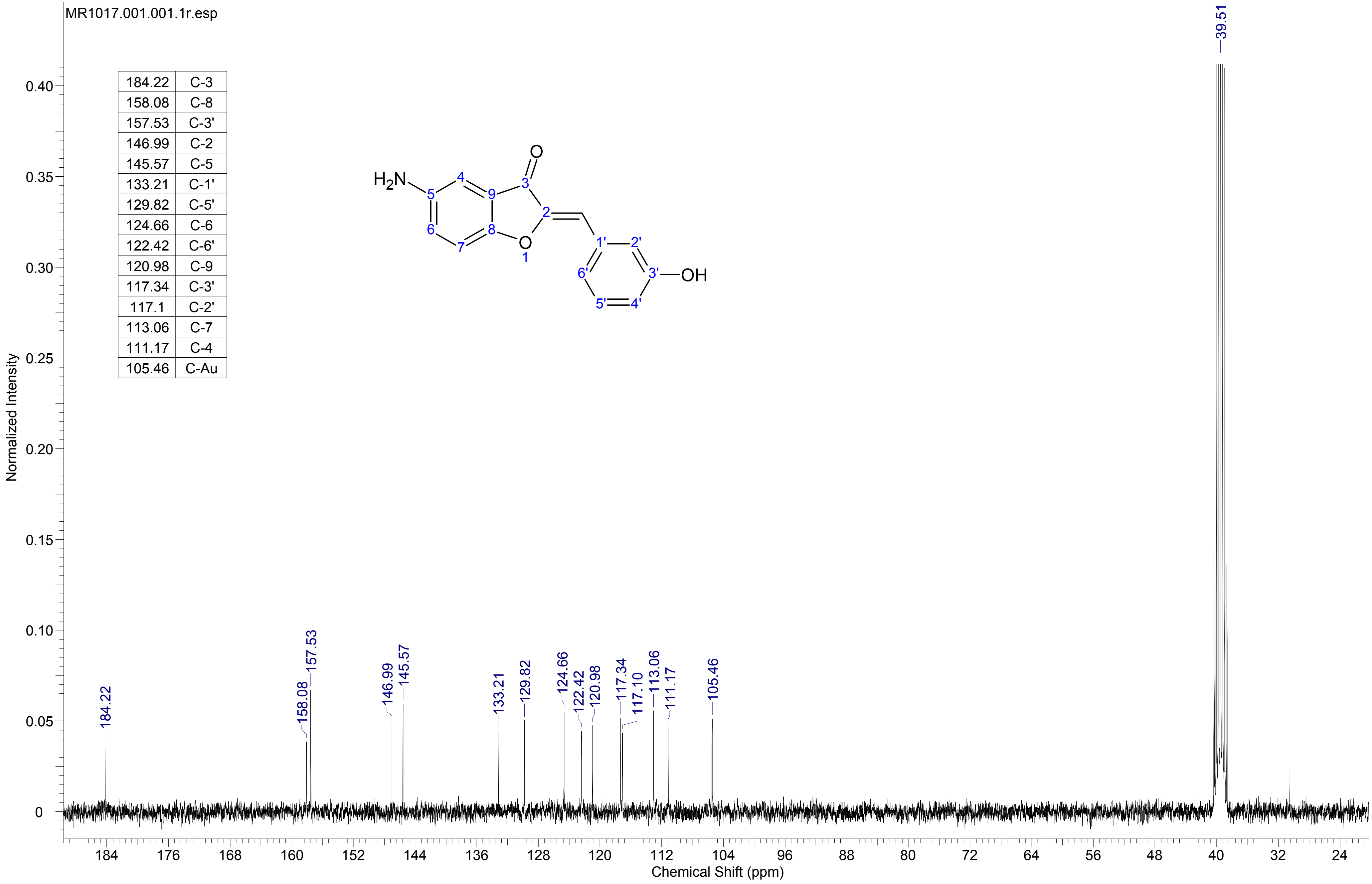

184.22	C-3
158.08	C-8
157.53	C-3'
146.99	C-2
145.57	C-5
133.21	C-1'
129.82	C-5'
124.66	C-6
122.42	C-6'
120.98	C-9
117.34	C-3'
117.1	C-2'
113.06	C-7
111.17	C-4
105.46	C-Au

C:\Documents and Settings\RMN\2018\MR1017\MR1017.001.001.1r.esp

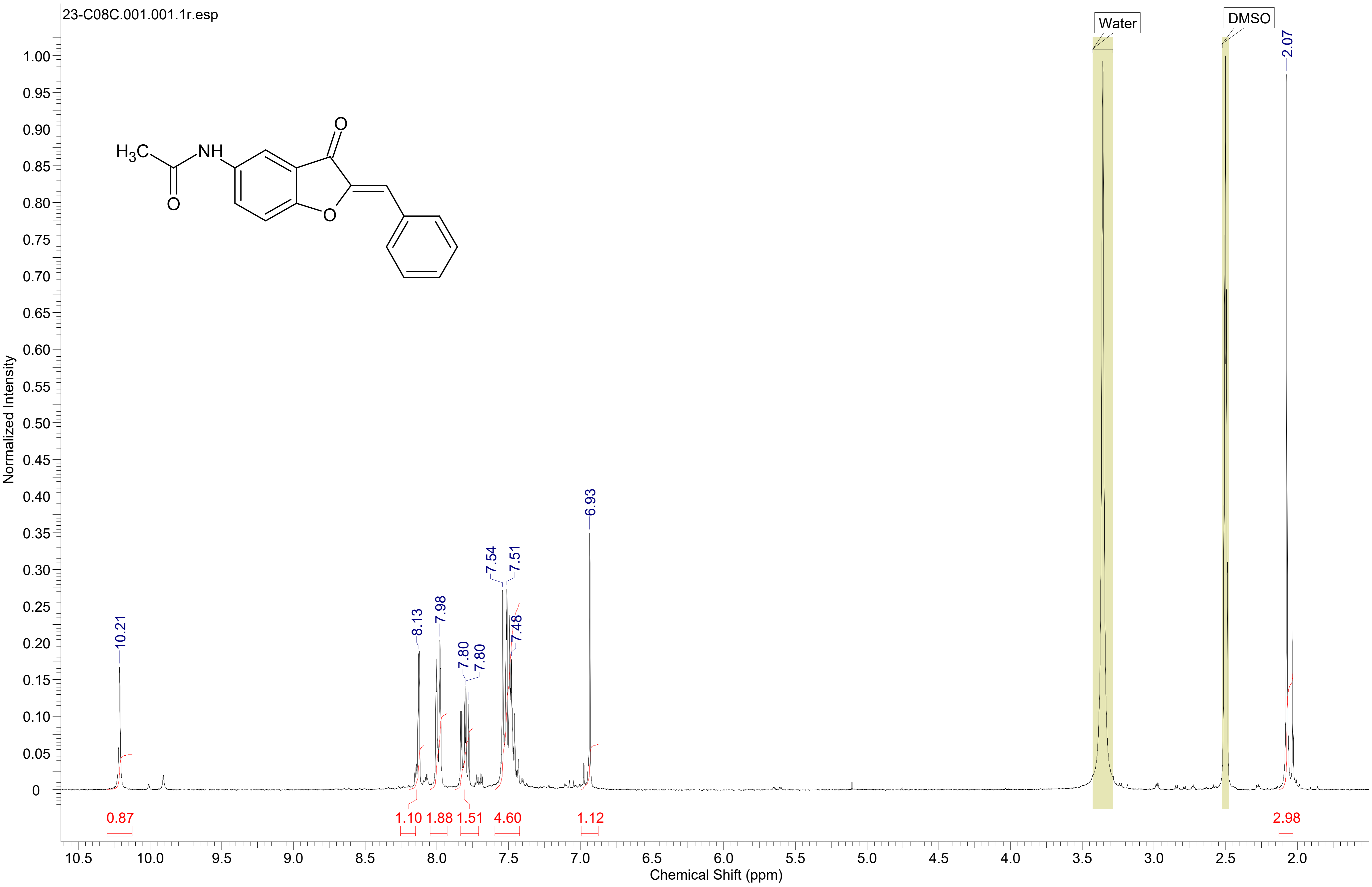

C:\Users\33674\Documents\RMN\MR12\23-C08C\23-C08C.001.001.1r.esp

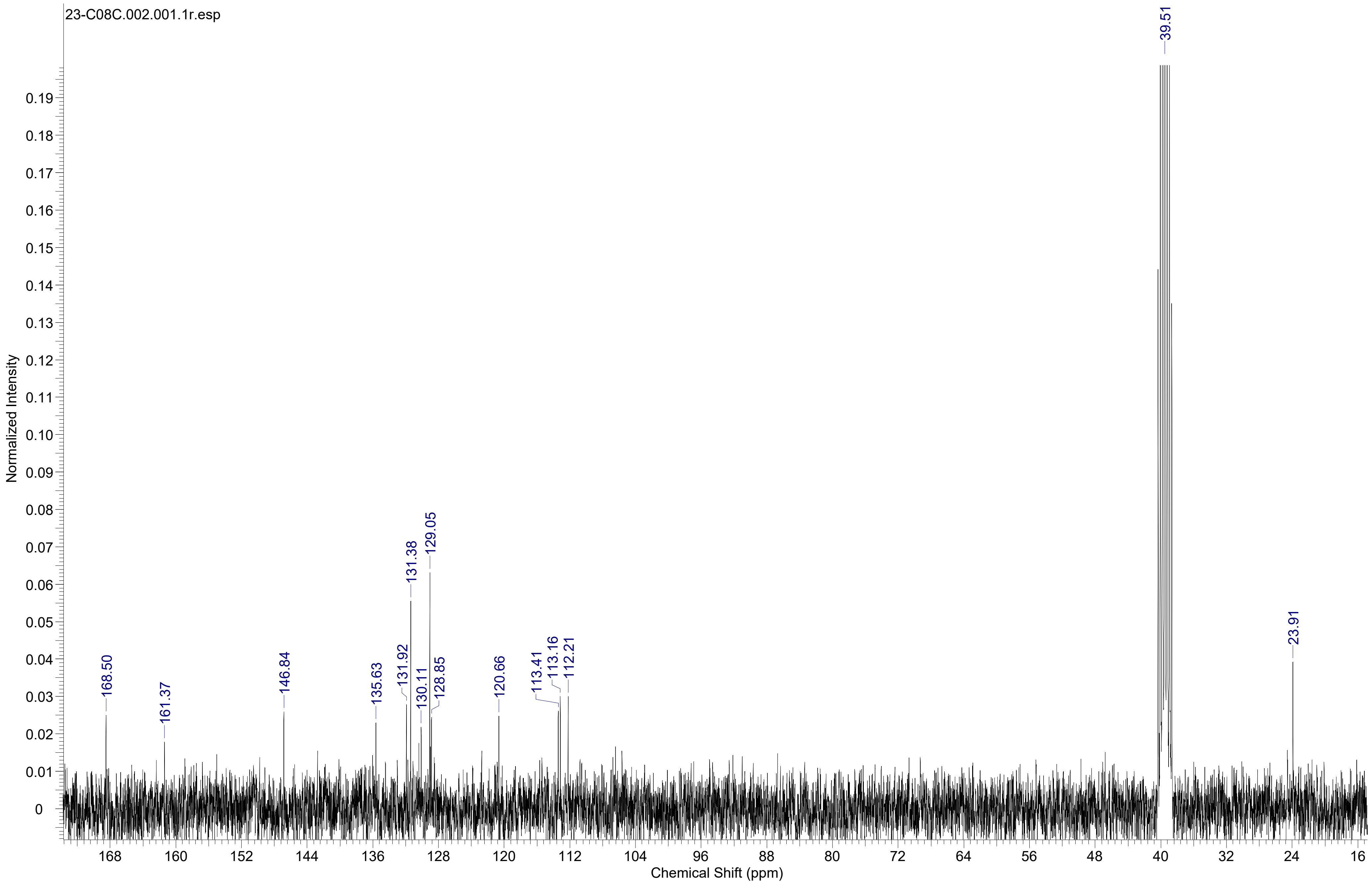

C:\Users\33674\Documents\RMN\MR12\23-C08C\23-C08C.002.001.1r.esp
